# Gold(I)-Catalyzed Domino Reaction for Furopyrans Synthesis

**DOI:** 10.3390/molecules25214976

**Published:** 2020-10-27

**Authors:** Marie Ruch, Nicolas Brach, Roméric Galéa, Patrick Wagner, Gaëlle Blond

**Affiliations:** Laboratoire d′Innovation Thérapeutique, Université de Strasbourg, CNRS, UMR 7200, 67000 Strasbourg, France; ruchm@unistra.fr (M.R.); nicolas.brach@etu.unistra.fr (N.B.); r.galea@unistra.fr (R.G.); pwagner@unistra.fr (P.W.)

**Keywords:** gold(I)-catalysis, domino reactions, electrocyclization, heterocycles, hetero Diels–Alder, furopyrans

## Abstract

We report herein an efficient synthesis of furopyran derivatives through a gold(I)-catalyzed domino reaction. The cascade reaction starts with two regioselective cyclizations, a 5-*endo*-dig and a 8-*endo*-dig, followed with a Grob-type fragmentation and a hetero Diels–Alder. The obtained furopyran derivatives contain fused and spiro-heterocycles. During this one-pot process, four bonds and four controlled stereogenic centers including a quaternary center are formed.

## 1. Introduction

**Figure 1:**
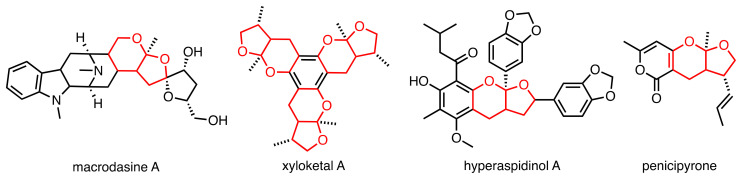
Representative natural products with 4*H*-furo[2,3-*b*]pyran scaffolds.

Furopyrans are an important heterocyclic motif. Among furopyran scaffolds, 4*H*-furo[2,3-*b*]pyrans bearing fused bicyclic *O*,*O*-acetals are present in a number of biologically active products such as macrodasine A, xyloketal A, hyperaspidinol A and penicipyrone (Figure 1) [1,2,3,4,5].

To access a furopyran core, the hetero Diels–Alder reaction (HDA) is one of the most efficient methods, coupling a dihydrofuran with α,β-unsaturated ketone [6,7,8,9,10,11]. Particularly, gold(I)-catalyzed reactions including HDA reactions have proven to be important tools for the construction of such scaffolds. Most of the gold(I)-triggered HDA reported so far rely on the in situ generation of the diene [12,13,14] or the dienophile [15,16,17,18,19,20,21,22]. However, a HDA between diene and dienophile generated in situ by gold-catalyzed transformations remains elusive [23]. 

**Scheme 1:**
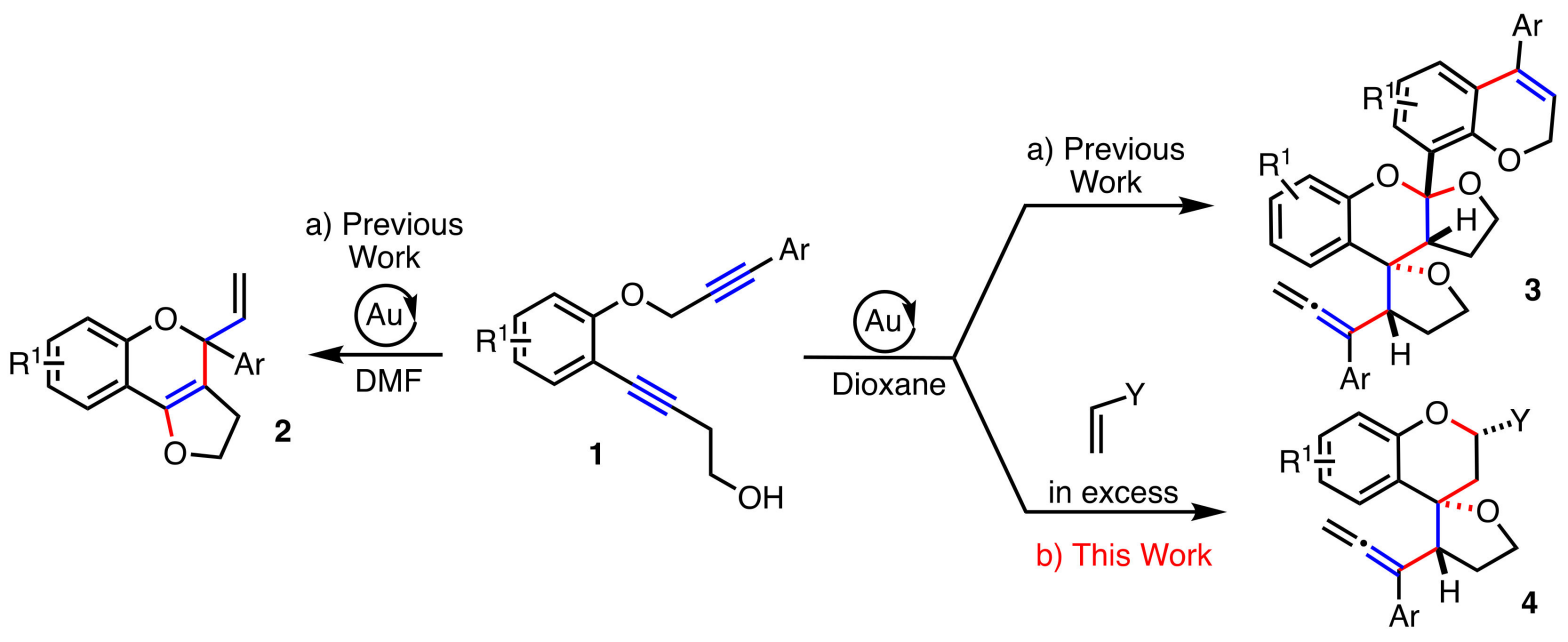
Gold(I)-catalyzed cascade reaction with **1**.

In this context, we have previously reported the synthesis of polycyclic molecules containing furopyran cores through a gold(I)-catalyzed domino reaction (Scheme 1) [24,25]. Particularly, we have described the synthesis of two classes of furopyrans, **2** and **3**, starting from the same source **1** and only changing the solvent of the reaction.

**Scheme 2:**
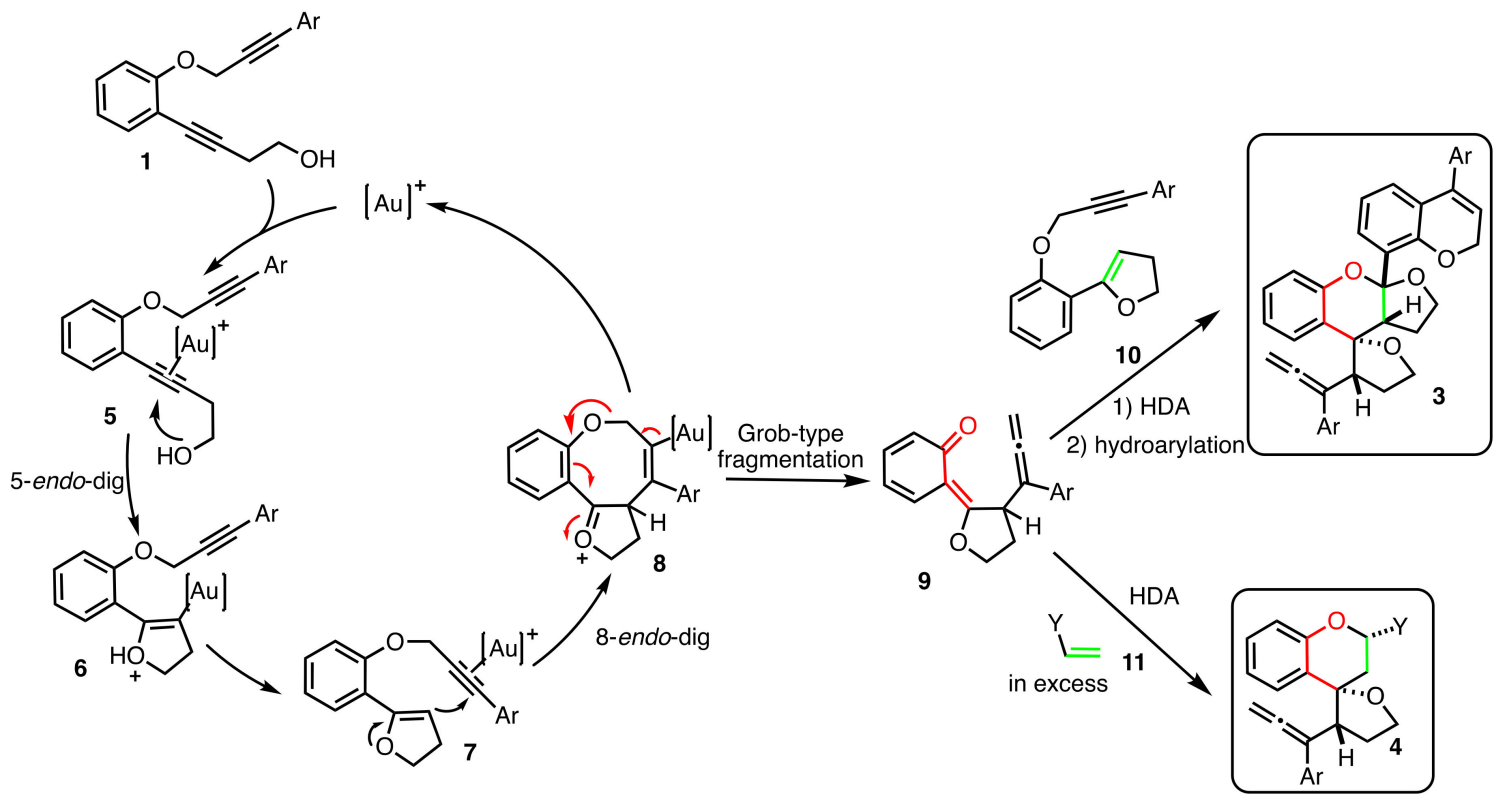
Proposed mechanism.

Indeed, in DMF, we have prepared a series of 4*H*-furo[3,2-*c*]pyrans **2**, while in 1,4-dioxane, complex *4H-*furo[2,3-*b*]pyrans **3** have been synthesized. Concerning the formation of **3** in 1,4-dioxane, the sequence includes two cyclizations: a 5-*endo*-dig and a regioselective 8-*endo*-dig cyclization giving the 8-membered ring **8** (Scheme 2). A Grob-type fragmentation allowed the formation of the diene **9**. The key step of this cascade reaction is a hetero Diels–Alder reaction (HDA) in which the formed diene **9** and the dienophile **10** are produced concomitantly. The formation of dienophile **10** comes from the protodeauration of intermediary **6**. The final gold-catalyzed hydroarylation produces **3** in good yield. Continuing these works, we have decided to introduce in the reaction mixture an excess of dienophile **11**. Indeed, starting with the same starting material **1** in 1,4-dioxane, intermediate **9** will react with the excess of dienophile **11** instead of dienophile **10** also present in the reaction mixture. We describe herein the synthesis of the resulting compound **4**.

## 2. Results

### 2.1. Dienophiles Screening

As previously described, the conditions to obtain **3** with good yield were the use of catalyst **A**, in 1,4-dioxane after 20 min at 60 °C under microwave irradiation. Our first investigation was the use of these reaction conditions in the presence of an excess of dihydrofuran; we obtained good yield of **4a** (64%, Entry 1, Table 1). We then decided to explore other common dienophiles [2] in order to increase the complexity of such scaffolds. The use of 4-phenylbut-3-yn-1-ol was motivated by obtaining the in situ formation of the 5-phenyl-2,3-dihydrofuran able to react as a dienophile in the reaction (entry 2) [20]. We obtained the formation of **5a** with 7% yield. With dihydropyran, the conversion was total, and the protected alcohol **6** was not isolated completely purely (around 20% yield) in the middle of an unidentified product (entry 3). Surprisingly, methyl or ethyl vinyl ether gave complete conversion into a complex mixture (entries 4 and 5). The same result was observed with dihydropyrrole (entry 6), phenyl vinyl thioether (entry 7) and furan-2,5-dione (entry 8). 

### 2.2. Scope of the Reaction

**Scheme 3:**
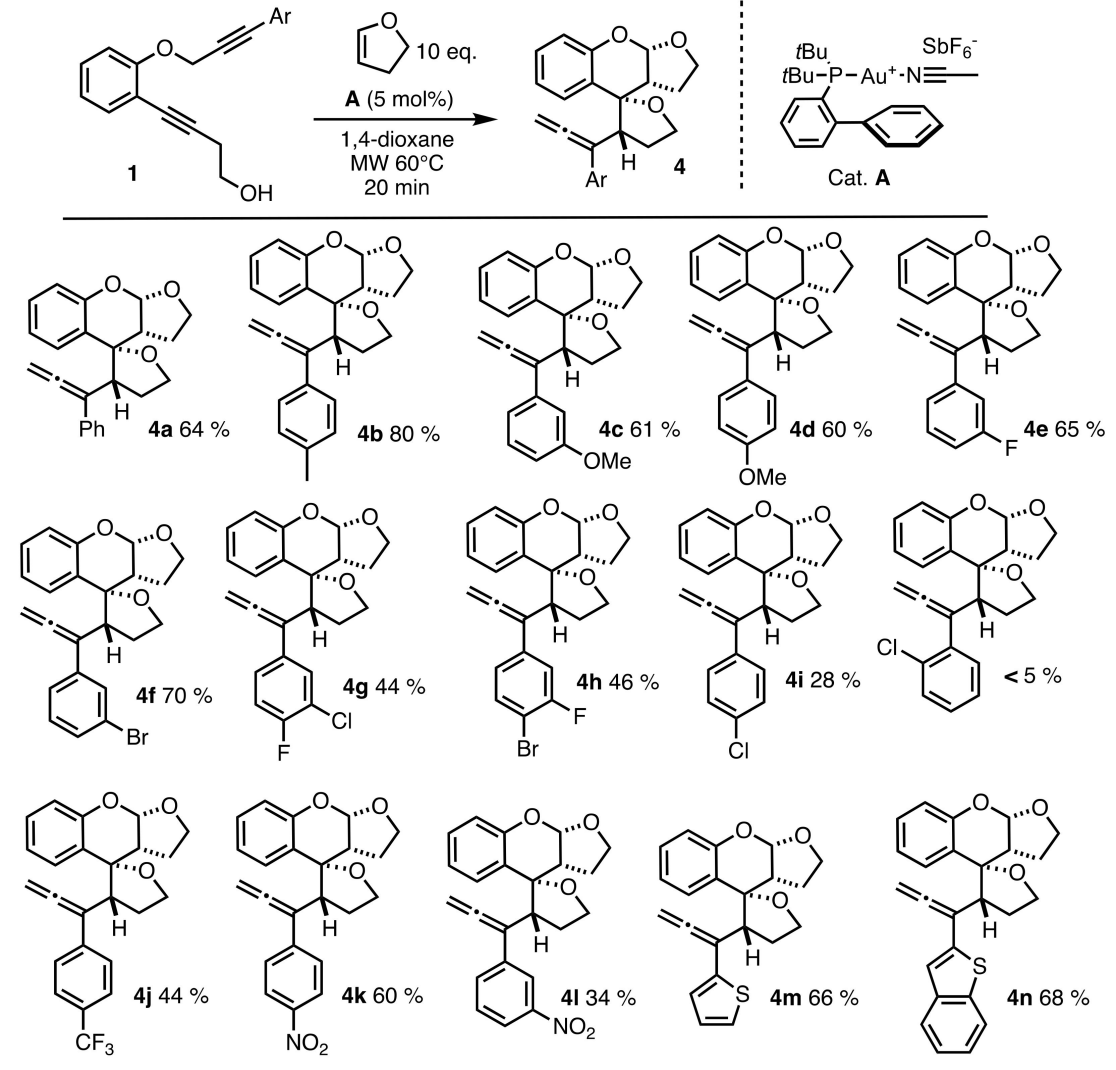
Scope of the reaction.

As only dihydrofuran gave a good result, we investigated the scope of the reaction using dihydrofuran with various compounds of type **1** with a range of aryls exhibiting electron-donating or -withdrawing groups at the meta or para positions (Scheme 3). Our best result was obtained with the tolyl group (**4b**, 80%). A para- or meta-methoxy group on the aryl ring made the reaction possible and **4c** and **4d** were isolated with 60% yield. Results are more disparate with halogen substitutions, where yields between 28 to 70% were obtained (**4e**–**4i**). As described previously, with ortho-substituted aryl groups, the steric hindrance hampers the reaction. Indeed, with an ortho-chloro substituted aryl, less than 5% of **4** was observed. With aryls exhibiting electron-withdrawing groups such as CF_3_ and NO_2_, yields between 34 and 60% (**4j**–**4l**) were obtained. Heteroaromatics such as thiophene and benzothiophene gave good results (**4m**–**4n**, more than 65%).

## 3. Conclusions

In conclusion, we have described the synthesis of new *4H-*furo[2,3-b]pyrans **4** via a gold-catalyzed domino reaction. The sequence includes two regioselective cyclizations, a 5-*endo*-dig and a 8-*endo*-dig, followed with a Grob-type fragmentation and a hetero Diels–Alder reaction. A total of 14 compounds have been described and isolated, with yields ranging between 28–80%.

## 4. Materials and Methods

### 4.1. General Information

All reagents, chemicals and dry solvents were purchased from commercial sources and used without purification. When mentioned that the reaction was conducted in dry media, glassware dried for several hours at 110 °C in an oven was used. Triethylamine (Et_3_N) and diisopropylamine (DIPA) were distilled from KOH in an S-tube prior to each experiment in which they were involved. Reactions were monitored by TLC (thin layer silica gel chromatography) using Merck silica gel 60 F254 on aluminum sheets. TLC plates were visualized under UV light and revealed with acidic *p*-anisaldehyde stain or KMnO_4_ stain. Crude products were purified by flash column chromatography on Merck silica gel Si 60 (40–63 μm). NMR spectra were recorded in CDCl_3_ on a Bruker Avance III BBFO+ probe spectrometer 400 MHz for ^1^H analyses and 100 MHz for ^13^C analyses. Proton chemical shifts are reported in ppm (δ), relatively to residual CHCl_3_ (δ 7.26 ppm). Multiplicities are reported as follows: singlet (s), doublet (d), triplet (t), quartet (q), quintet (quint), broad singlet (bs), broad doublet (bd) combinations or multiplet (m). Coupling constant values J are given in Hz. Carbon chemical shifts are reported in ppm (δ), relative to the internal standard CDCl_3_ (δ 77.23 ppm). ^1^H and ^13^C NMR signals were assigned mostly on the basis of 2D-NMR (COSY, HSQC, HMBC) experiments. High resolution mass spectral analyses (HRMS) were performed using an Agilent 1200 RRLC HPLC chain and an Agilent 6520 Accurate mass QToF. Infrared spectra (IR) were recorded on a FTIR Thermo Nicollet ATR 380, Diamant Spectrometer.

All compounds **1a**–1**e** and **1h**–1**n** have been already described [6].

### 4.2. Synthesis of **1f** and **1g**

Anhydrous THF and distilled Et_3_N were mixed in a 2-necked flask under argon. The iodoaryl (1.5 eq.), PdCl_2_(PPh_3_) (0.03 eq.) and CuI (0.06 eq.) were added to the flask and this mixture was degassed with argon for 15 min. The alkyne (1 eq.) was dissolved in THF degassed with argon for 15 min and added to the 2-necked flask. The mixture was stirred overnight at room temperature (20 °C) and monitored by TLC (9/1 pent/Et_2_O). Once the TLC showed complete conversion of the true alkyne, the reaction mixture was filtered through a pad of Celite with CH_2_Cl_2_ as eluent and concentrated to give the crude product as a dark-brown solid. The latter was purified by flash column chromatography (98/2 pent/Et_2_O) to afford the pure *tert*-butyldimethylsilyl-protected coupling product. TBAF (1 eq.) was added to a solution of this latter protected product in THF at 0 °C. This mixture was stirred at room temperature until the TLC (9/1 pent/Et_2_O) showed complete conversion of the starting material. The reaction mixture was dissolved in a saturated aqueous NH_4_Cl solution. The aqueous phase was extracted with CH_2_Cl_2_. The gathered organic layers were dried over MgSO_4_, filtered and concentrated to afford the crude as a yellowish oil. The latter was purified by flash column chromatography (6/4 pent/Et_2_O) to afford the pure deprotected compound (**1f** and **1g**).

#### 4.2.1. Synthesis of 4-(2-((3-(3-bromophenyl)prop-2-yn-1-yl)oxy)phenyl)but-3-yn-1-ol **1f**

Compound 1f was prepared following the general procedure using alkyne (500 mg, 1.6 mmol, 1 eq.), THF (10 mL), PdCl_2_(PPh_3_)_2_ (34 mg, 0.05 mmol, 0.03 eq.), CuI (18 mg, 0,1 mmol, 0.06 eq.), 3-bromoiodobenzene (679 mg, 2.4 mmol, 1.5 eq.), Et_3_N (4 mL) and TBAF (1.6 mL, 1.60 mmol, 1 eq.) in THF (10 mL) for the deprotection. Purification by chromatography on silica gel afforded compound 1f (81%, 499 mg, 1.405 mmol in two steps) as an orange oil. ^1^H NMR (400 MHz, CDCl_3_) δ 7.67 (t, *J* = 1.7 Hz, 1H), 7.55 (ddd, *J* = 8.0, 2.1, 1.1 Hz, 1H), 7.50 (dd, *J* = 7.6, 1.7 Hz, 1H), 7.45 (dt, *J* = 7.8, 1.3 Hz, 1H), 7.38 (ddd, *J* = 8.4, 7.5, 1.8 Hz, 1H), 7.26 (t, *J* = 7.9 Hz, 1H), 7.15 (dd, *J* = 8.4, 1.1 Hz, 1H), 7.05 (td, *J* = 7.5, 1.0 Hz, 1H), 5.07 (s, 2H), 3.92 (q, *J* = 5.2 Hz, 2H), 2.84 (t, *J* = 6.1 Hz, 2H), 2.35 (s, 1H). ^13^C NMR (101 MHz, CDCl_3_) δ 158.3, 134.6, 133.6, 132.0, 130.5, 129.9, 129.3, 124.3, 122.2, 121.6, 113.5, 112.9, 91.3, 86.1, 85.1, 78.9, 61.2, 57.4, 24.4. HRMS ESI: Calculated for C_19_H_15_BrO_2_ [M + H]^+^ 355.0334, found 355.0311 (Diff.: 4.58 ppm).

#### 4.2.2. 4-(2-((3-(3-chloro-4-fluorophenyl)prop-2-yn-1-yl)oxy)phenyl)but-3-yn-1-ol **1g**

Compound 1g was prepared following the general procedure using alkyne (500 mg, 1.6 mmol, 1 eq.), THF (10 mL), PdCl_2_(PPh_3_)_2_ (34 mg, 0.05 mmol, 0.03 eq.), CuI (18 mg, 0,1 mmol, 0.06 eq.), 3-chloro-4-chloroiodobenzene (615 mg, 2.4 mmol, 1.5 eq.), Et_3_N (4 mL) and TBAF (0.86 mL, 0.86 mmol, 1 eq.) in THF (10 mL) for the deprotection. Purification by chromatography on silica gel afforded compound 1g (72%, 380 mg, 1.15 mmol in two steps) of as an orange oil. ^1^H NMR (400 MHz, CDCl_3_) δ 7.67 (dd, *J* = 7.0, 2.1 Hz, 1H), 7.60 (dd, *J* = 7.6, 1.7 Hz, 1H), 7.54–7.44 (m, 2H), 7.30–7.20 (m, 2H), 7.15 (td, *J* = 7.5, 1.0 Hz, 1H), 5.15 (s, 2H), 4.02 (t, *J* = 6.1 Hz, 2H), 2.94 (t, *J* = 6.1 Hz, 2H), 2.49 (s, 1H).^13^C NMR (101 MHz, CDCl_3_) δ 158.4 (d, *J* = 252.5 Hz), 158.3, 134.1, 133.6, 131.9 (d, *J* = 7.0 Hz), 129.3, 121.6, 121.3 (d, *J* = 18.0 Hz), 119.5 (d, *J* = 4.0 Hz), 116.8 (d, *J* = 21.0 Hz), 113.5, 112.9, 91.3, 85.4, 84.6, 78.9, 61.1, 57.3, 24.3. HRMS ESI: Calculated for C_19_H_14_ClFO_2_ [M + H]^+^ 329.0745, found 329.0723 (Diff.: 4.91 ppm).

### 4.3. General Procedure for Gold(I) Catalyzed Cascade Reactions: Preparation of **4a**–**n**

Substrate 1 (1 eq.) and dihydrofuran (10 eq.) were placed in a 0.5–2 mL microwave reactor and dissolved in anhydrous 1,4-dioxane. Catalyst A (0.05 eq.) was added into the reactor. Once all the reagents were dissolved, the reactor was placed into the microwave for 20 min at 60 °C. The reaction mixture was filtered through a pad of Celite with CH_2_Cl_2_ as eluent. After solvent evaporation under reduced pressure, purification of the crude by flash column chromatography provided the furopyran adduct 4.

#### 4.3.1. Synthesis of (2S,3R,3a′R,9a′S)-3-(1-phenylpropa-1,2-dien-1-yl)-2′,3′,3a′,4,5,9a′-hexahydro-3H-spiro[furan-2,4′-furo[2,3-b]chromene] **4a**

Compound **4a** was prepared following the general procedure using compound **1a** (20 mg, 0.072 mmol, 1 eq.), dihydrofuran (51 mg, 0.72 mmol, 10 eq.) and catalyst **A** (3 mg, 0.004 mmol, 0.05 eq.) in 1,4-dioxane (0.4 mL). Purification by chromatography on silica gel (8/2 pentane/Et_2_O) afforded compound **4a** (64%, 16 mg, 0.046 mmol) as an amorphous white solid. ^1^H NMR (400 MHz, CDCl_3_) δ 7.28–7.24 (m, 1H), 7.23–7.11 (m, 5H), 7.11–7.05 (m, 1H), 6.89 (td, *J* = 7.5, 1.2 Hz, 1H), 6.62 (dd, *J* = 8.1, 1.2 Hz, 1H), 5.31 (d, *J* = 5.6 Hz, 1H), 4.81 (dd, *J* = 11.7, 1.3 Hz, 1H), 4.42 (d, *J* = 11.7 Hz, 1H), 4.33 (td, *J* = 8.4, 3.8 Hz, 1H), 4.10 (td, *J* = 8.4, 7.3 Hz, 1H), 3.87–3.73 (m, 2H), 3.48–3.37 (m, 1H), 2.75 (td, *J* = 9.4, 5.6 Hz, 1H), 2.48 (dtd, *J* = 12.8, 7.3, 3.8 Hz, 1H), 2.30 (dq, *J* = 12.7, 8.5 Hz, 1H), 2.13–1.99 (m, 1H), 1.70 (dtd, *J* = 12.9, 9.6, 8.4 Hz, 1H). ^13^C NMR (101 MHz, CDCl_3_) δ 209.3, 153.0, 137.0, 129.0, 128.4, 127.5, 126.8, 126.5, 125.2, 120.8, 116.0, 105.1, 101.6, 85.0, 78.3, 67.8, 67.0, 49.7, 49.6, 33.3, 27.0. HRMS ESI: Calculated for C_23_H_23_O_3_ [M + H]^+^ 347.1647, found 347.1642 (Diff.: 0.31 ppm).

#### 4.3.2. Synthesis of (2S,3R,3a′R,9a′S)-3-(1-(p-tolyl)propa-1,2-dien-1-yl)-2′,3′,3a′,4,5,9a′-hexahydro-3H-spiro[furan-2,4′-furo[2,3-b]chromene] **4b**

Compound **4b** was prepared following the general procedure using compound **1b** (50 mg, 0.172 mmol, 1 eq.), dihydrofuran (120 mg, 1.72 mmol, 10 eq.) and catalyst **A** (7 mg, 0.009 mmol, 0.05 eq.) in 1,4-dioxane (0.4 mL). Purification by chromatography on silica gel (8/2 pentane/Et_2_O) afforded compound **4b** (80%, 50 mg, 0.138 mmol) as an orange oil. ^1^H NMR (400 MHz, CDCl_3_) δ 7.24 (dd, *J* = 7.7, 1.6 Hz, 1H), 7.10 (td, *J* = 7.7, 1.7 Hz, 1H), 7.05 (d, *J* = 8.4 Hz, 2H), 7.01 (d, *J* = 8.4 Hz, 2H), 6.90 (dd, *J* = 7.5, 1.2 Hz, 1H), 6.64 (dd, *J* = 8.1, 1.2 Hz, 1H), 5.25 (d, *J* = 5.7 Hz, 1H), 4.76 (d, *J* = 11.5 Hz, 1H), 4.32 (td, *J* = 8.8, 3.6 Hz, 2H), 4.09 (td, *J* = 8.7, 7.2 Hz, 1H), 3.88–3.70 (m, 2H), 3.38 (dd, *J* = 8.9, 7.1 Hz, 1H), 2.72 (td, *J* = 9.5, 5.6 Hz, 1H), 2.44 (dtd, *J* = 12.7, 7.1, 3.5 Hz, 1H), 2.38–2.22 (m, 4H), 2.15–1.99 (m, 1H), 1.70 (dq, *J* = 12.8, 8.9 Hz, 1H). ^13^C NMR (101 MHz, CDCl_3_) δ 209.1, 153.0, 136.5, 134.1, 129.1, 129.0, 127.6, 126.3, 125.2, 120.7, 116.0, 104.6, 101.5, 85.0, 78.1, 67.7, 67.0, 49.9, 49.8, 33.3, 27.0, 21.1. HRMS ESI: Calculated for C_24_H_25_O_3_ [M + H]^+^ 361.1804, found 361.1793 (Diff.: 1.59 ppm).

#### 4.3.3. Synthesis of (2S,3R,3a′R,9a′S)-3-(1-(3-methoxyphenyl)propa-1,2-dien-1-yl)-2′,3′,3a′,4,5,9a′-hexahydro-3H-spiro[furan-2,4′-furo[2,3-b]chromene] **4c**

Compound **4c** was prepared following the general procedure using compound **1c** (51 mg, 0.167 mmol, 1 eq.), dihydrofuran (117 mg, 1.67 mmol, 10 eq.) and catalyst **A** (6 mg, 0.008 mmol, 0.05 eq.) in 1,4-dioxane (1 mL). Purification by chromatography on silica gel (8/2 pentane/Et_2_O) afforded compound **4c** (61%, 39 mg, 0.102 mmol) as an amorphous yellow solid. ^1^H NMR (400 MHz, CDCl_3_) δ 7.29–7.20 (m, 1H), 7.16–7.05 (m, 2H), 6.88 (td, *J* = 7.5, 1.2 Hz, 1H), 6.78 (dt, *J* = 7.9, 1.2 Hz, 1H), 6.70–6.59 (m, 3H), 5.35 (d, *J* = 5.7 Hz, 1H), 4.81 (dd, *J* = 11.7, 1.4 Hz, 1H), 4.52–4.37 (m, 1H), 4.31 (td, *J* = 8.4, 3.9 Hz, 1H), 4.08 (td, *J* = 8.4, 7.4 Hz, 1H), 3.94–3.65 (m, 5H), 3.47–3.26 (m, 1H), 2.75 (td, *J* = 9.5, 5.7 Hz, 1H), 2.46 (dtd, *J* = 12.7, 7.3, 3.9 Hz, 1H), 2.28 (dq, *J* = 12.6, 8.4 Hz, 1H), 2.05 (dddd, *J* = 12.9, 9.2, 7.2, 4.8 Hz, 1H), 1.69 (ddt, *J* = 12.9, 9.9, 8.7 Hz, 1H). ^13^C NMR (101 MHz, CDCl_3_) δ 209.2, 159.6, 153.0, 138.5, 129.2, 129.0, 127.5, 125.1, 120.7, 118.8, 116.0, 112.4, 112.3, 105.2, 101.6, 84.9, 78.3, 67.8, 66.9, 55.2, 49.6, 49.6, 33.3, 27.0. HRMS ESI: Calculated for C_24_H_25_O_4_ [M + H]^+^ 377.1753, found 377.1750 (Diff.: −0.74 ppm).

#### 4.3.4. Synthesis of (2S,3R,3a′R,9a′S)-3-(1-(4-methoxyphenyl)propa-1,2-dien-1-yl)-2′,3′,3a′,4,5,9a′-hexahydro-3H-spiro[furan-2,4′-furo[2,3-b]chromene] **4d**

Compound **4d** was prepared following the general procedure using compound **1d** (49 mg, 0.160 mmol, 1 eq.), dihydrofuran (112 mg, 1.60 mmol, 10 eq.) and catalyst **A** (7 mg, 0.009 mmol, 0.05 eq.) in 1,4-dioxane (1 mL). Purification by chromatography on silica gel (8/2 pentane/Et_2_O) afforded compound **4d** (60%, 37 mg, 0.098 mmol) as a yellow oil. ^1^H NMR (400 MHz, CDCl_3_) δ 7.24 (dd, *J* = 7.6, 1.7 Hz, 1H), 7.12–7.03 (m, 3H), 6.88 (td, *J* = 7.5, 1.2 Hz, 1H), 6.79–6.70 (m, 2H), 6.63 (dd, *J* = 8.0, 1.2 Hz, 1H), 5.30 (d, *J* = 5.7 Hz, 1H), 4.78 (dd, *J* = 11.4, 1.3 Hz, 1H), 4.38 (dd, *J* = 11.4, 1.0 Hz, 1H), 4.32 (td, *J* = 8.4, 3.8 Hz, 1H), 4.08 (td, *J* = 8.5, 7.3 Hz, 1H), 3.85–3.72 (m, 6H), 3.40–3.32 (m, 1H), 2.74 (td, *J* = 9.5, 5.7 Hz, 1H), 2.46 (dtd, *J* = 12.8, 7.2, 3.7 Hz, 1H), 2.28 (dq, *J* = 12.8, 8.5 Hz, 1H), 2.06 (dddd, *J* = 12.9, 9.3, 7.3, 4.7 Hz, 1H), 1.85–1.66 (m, 1H). ^13^C NMR (101 MHz, CDCl_3_) δ 208.9, 158.5, 152.9, 129.1, 128.9, 127.4, 127.4, 125.1, 120.6, 115.8, 113.7, 104.5, 101.4, 84.9, 78.0, 67.6, 66.8, 55.2, 49.8, 49.6, 33.1, 26.9. HRMS ESI: Calculated for C_24_H_24_NaO_4_ [M + Na]^+^ 399.1572, found 399.1551 (Diff.: 4.13 ppm).

#### 4.3.5. Synthesis of (2S,3R,3a′R,9a′S)-3-(1-(3-fluorophenyl)propa-1,2-dien-1-yl)-2′,3′,3a′,4,5,9a′-hexahydro-3H-spiro[furan-2,4′-furo[2,3-b]chromene] **4e**

Compound **4e** was prepared following the general procedure using compound **1e** (50 mg, 0.170 mmol, 1 eq.), dihydrofuran (119 mg, 1.70 mmol, 10 eq.) and catalyst **A** (7 mg, 0.009 mmol, 0.05 eq.) in 1,4-dioxane (1 mL). Purification by chromatography on silica gel (8/2 pentane/Et_2_O) afforded compound **4e** (65%, 41 mg, 0.11 mmol) as orange oil. ^1^H NMR (400 MHz, CDCl_3_) δ 7.28–7.22 (m, 1H), 7.18–7.05 (m, 2H), 6.96–6.86 (m, 2H), 6.85–6.75 (m, 2H), 6.60 (dd, *J* = 8.1, 1.2 Hz, 1H), 5.40 (d, *J* = 5.8 Hz, 1H), 4.87 (dd, *J* = 12.0, 1.3 Hz, 1H), 4.52 (d, *J* = 11.9 Hz, 1H), 4.31 (td, *J* = 8.4, 4.3 Hz, 1H), 4.09 (q, *J* = 8.1 Hz, 1H), 3.89–3.75 (m, 2H), 3.39 (dd, *J* = 8.1, 6.8 Hz, 1H), 2.79 (td, *J* = 9.4, 5.8 Hz, 1H), 2.50 (dtd, *J* = 12.8, 7.5, 4.2 Hz, 1H), 2.38–2.24 (m, 1H), 2.06 (dddd, *J* = 12.9, 9.3, 6.6, 5.5 Hz, 1H), 1.83–1.64 (m, 1H).^13^C NMR (101 MHz, CDCl_3_) δ 209.2, 162.8 (d, *J* = 244.9 Hz), 152.9, 139.4 (d, *J* = 7.6 Hz), 129.6 (d, *J* = 8.4 Hz), 129.1, 127.4, 125.2, 121.8 (d, *J* = 2.7 Hz), 120.9, 116.1, 113.6 (d, *J* = 21.2 Hz), 113.5 (d, *J* = 23.2 Hz), 104.9, 101.6, 84.9, 78.8, 67.8, 66.8, 49.5, 49.1, 33.1, 27.0. ^19^F NMR (376 MHz, CDCl_3_) δ −113.54–−113.72 (m). HRMS ESI: Calculated for C_23_H_22_FO_3_ [M + H]^+^ 365.1553, found 365.1546 (Diff.: 0.51 ppm).

#### 4.3.6. Synthesis of (2S,3R,3a′R,9a′S)-3-(1-(3-bromophenyl)propa-1,2-dien-1-yl)-2′,3′,3a′,4,5,9a′-hexahydro-3H-spiro[furan-2,4′-furo[2,3-b]chromene] **4f**

Compound **4f** was prepared following the general procedure using compound **1f** (53 mg, 0.149 mmol, 1 eq.), dihydrofuran (104 mg, 1.49 mmol, 10 eq.) and catalyst **A** (7 mg, 0.009 mmol, 0.05 eq.) in 1,4-dioxane (1 mL). Purification by chromatography on silica gel (8/2 pentane/Et_2_O) afforded compound **4f** (70%, 45 mg, 0.104 mmol) as an amorphous yellow solid. ^1^H NMR (400 MHz, CDCl_3_) δ 7.24 (dd, *J* = 7.6, 1.7 Hz, 1H), 7.22–7.18 (m, 1H), 7.12 (q, *J* = 1.4 Hz, 1H), 7.10–7.00 (m, 3H), 6.89 (td, *J* = 7.5, 1.2 Hz, 1H), 6.57 (dd, *J* = 8.1, 1.2 Hz, 1H), 5.43 (d, *J* = 5.8 Hz, 1H), 4.89 (dd, *J* = 11.9, 1.5 Hz, 1H), 4.58 (dd, *J* = 12.0, 1.1 Hz, 1H), 4.29 (td, *J* = 8.4, 4.6 Hz, 1H), 3.78 (dd, *J* = 8.4, 6.1 Hz, 2H), 3.37 (tt, *J* = 7.2, 1.4 Hz, 1H), 2.79 (td, *J* = 9.4, 5.8 Hz, 1H), 2.55–2.42 (m, 1H), 2.27 (dtd, *J* = 12.8, 8.2, 7.0 Hz, 1H), 2.12–1.98 (m, 1H), 1.69 (ddt, *J* = 12.9, 9.7, 8.4 Hz, 1H). ^13^C NMR (101 MHz, CDCl_3_) δ 209.1, 152.9, 139.1, 129.6 (3C), 129.1, 127.2, 125.1, 124.8, 122.4, 121.0, 116.1, 105.0, 101.6, 84.9, 78.9, 67.8, 66.7, 49.4, 48.9, 32.9, 26.9. HRMS ESI: Calculated for C_23_H_21_BrNaO_3_ [M + Na]^+^ 447.0572, found 447.0567 (Diff.: −0.49 ppm).

#### 4.3.7. Synthesis of (2S,3R,3a′R,9a′S)-3-(1-(3-chloro-4-fluorophenyl)propa-1,2-dien-1-yl)-2′,3′,3a′,4,5,9a′-hexahydro-3H-spiro[furan-2,4′-furo[2,3-b]chromene] **4g**

Compound **4g** was prepared following the general procedure using compound **1g** (52 mg, 0.16 mmol, 1 eq.), dihydrofuran (112 mg, 1.6 mmol, 10 eq.) and catalyst **A** (8 mg, 0.010 mmol, 0.05 eq.) in 1,4-dioxane (1 mL). Purification by chromatography on silica gel (8/2 pentane/Et_2_O) afforded compound **4g** (44%, 28 mg, 0.070 mmol) as a yellow oil. ^1^H NMR (400 MHz, CDCl_3_) δ 7.27–7.21 (m, 1H), 7.05 (ddd, *J* = 8.1, 7.3, 1.7 Hz, 1H), 7.00–6.85 (m, 4H), 6.55 (dd, *J* = 8.0, 1.2 Hz, 1H), 5.48 (d, *J* = 5.9 Hz, 1H), 4.92 (dt, *J* = 11.9, 1.2 Hz, 1H), 4.66 (dt, *J* = 11.8, 1.2 Hz, 1H), 4.28 (td, *J* = 8.4, 5.1 Hz, 1H), 4.07 (td, *J* = 8.2, 7.1 Hz, 1H), 3.82–3.75 (m, 2H), 3.35 (ddt, *J* = 7.7, 6.2, 1.5 Hz, 1H), 2.82 (td, *J* = 9.4, 5.9 Hz, 1H), 2.52 (dtd, *J* = 12.8, 7.8, 5.1 Hz, 1H), 2.28 (dddd, *J* = 13.2, 8.4, 7.2, 6.1 Hz, 1H), 2.11–1.98 (m, 1H), 1.68 (ddt, *J* = 12.9, 9.5, 8.3 Hz, 1H). ^13^C NMR (101 MHz, CDCl_3_) δ 208.9, 156.9 (d, *J* = 256.6 Hz), 152.8, 134.0 (d, *J* = 3.3 Hz), 129.1, 128.7, 127.1, 125.9 (d, *J* = 7.1 Hz), 125.3, 121.1, 120.6 (d, *J* = 17.8 Hz), 116.1, 116.0 (d, *J* = 21.1 Hz), 104.8, 101.7, 84.8, 79.1, 67.9, 66.5, 49.2, 48.9, 32.8, 26.9. HRMS ESI: Calculated for C_23_H_21_ClFO_3_ [M + H]^+^ 399.1163, found 399.1165 (Diff.: −0.61 ppm).

#### 4.3.8. Synthesis of (2S,3R,3a′R,9a′S)-3-(1-(4-bromo-3-fluorophenyl)propa-1,2-dien-1-yl)-2′,3′,3a′,4,5,9a′-hexahydro-3H-spiro[furan-2,4′-furo[2,3-b]chromene] **4h**

Compound **4h** was prepared following the general procedure using compound **1h** (50 mg, 0.152 mmol, 1 eq.), dihydrofuran (106 mg, 1.52 mmol, 10 eq.) and catalyst **A** (6 mg, 0.008 mmol, 0.05 eq.) in 1,4-dioxane (1 mL). Purification by chromatography on silica gel (8/2 pentane/Et_2_O) afforded compound **4h** (46%, 24 mg, 0.070 mmol) as a yellow oil. ^1^H NMR (400 MHz, CDCl_3_) δ 7.32 (dd, *J* = 8.6, 7.3 Hz, 1H), 7.24 (dd, *J* = 7.5, 1.8 Hz, 1H), 7.07 (ddd, *J* = 8.1, 7.3, 1.7 Hz, 1H), 6.88 (td, *J* = 7.5, 1.2 Hz, 1H), 6.84–6.75 (m, 2H), 6.58 (dd, *J* = 8.1, 1.2 Hz, 1H), 5.45 (d, *J* = 5.9 Hz, 1H), 4.91 (dd, *J* = 12.1, 1.4 Hz, 1H), 4.59 (dd, *J* = 12.2, 1.0 Hz, 1H), 4.30 (td, *J* = 8.4, 4.7 Hz, 1H), 4.08 (q, *J* = 7.9 Hz, 1H), 3.78 (dd, *J* = 8.4, 6.1 Hz, 2H), 3.35 (t, *J* = 7.1 Hz, 1H), 2.81 (td, *J* = 9.3, 5.9 Hz, 1H), 2.51 (dtd, *J* = 12.5, 7.7, 4.7 Hz, 1H), 2.27 (dq, *J* = 12.9, 7.6 Hz, 1H), 2.05 (ddt, *J* = 12.7, 9.4, 6.1 Hz, 1H), 1.69 (dq, *J* = 13.0, 8.5 Hz, 1H). ^13^C NMR (101 MHz, CDCl_3_) δ 209.1, 158.8 (d, *J* = 246.4 Hz), 152.9, 138.6 (d, *J* = 6.9 Hz), 132.9 (d, *J* = 1.0 Hz), 129.2, 127.3, 125.4, 122.9 (d, *J* = 3.2 Hz), 121.1, 116.2, 114.5 (d, *J* = 23.5 Hz), 106.8 (d, *J* = 21.2 Hz), 104.86 (d, *J* = 2.2 Hz), 101.7, 84.9, 79.3, 67.9, 66.6, 49.4, 48.7, 32.9, 27.0. HRMS ESI: Calculated for C_23_H_20_BrFNaO_3_ [M + Na]^+^ 465.0478, found 465.0464 (Diff.: 0.65 ppm).

#### 4.3.9. Synthesis of (2S,3R,3a′R,9a′S)-3-(1-(4-chlorophenyl)propa-1,2-dien-1-yl)-2′,3′,3a′,4,5,9a′-hexahydro-3H-spiro[furan-2,4′-furo[2,3-b]chromene] **4i**

Compound **4i** was prepared following the general procedure using compound **1i** (50 mg, 0.161 mmol, 1 eq.), dihydrofuran (113 mg, 1.61 mmol, 10 eq.) and catalyst **A** (8 mg, 0.010 mmol, 0.05 eq.) in 1,4-dioxane (1 mL). Purification by chromatography on silica gel (8/2 pentane/Et_2_O) afforded compound **4i** (28%, 17 mg, 0.045 mmol) as a colorless oil. ^1^H NMR (400 MHz, CDCl_3_) δ 7.25 (dd, *J* = 7.6, 1.7 Hz, 1H), 7.16–7.11 (m, 2H), 7.09–7.01 (m, 3H), 6.88 (td, *J* = 7.5, 1.2 Hz, 1H), 6.59 (dd, *J* = 8.1, 1.2 Hz, 1H), 5.38 (d, *J* = 5.8 Hz, 1H), 4.86 (dd, *J* = 11.9, 1.4 Hz, 1H), 4.51 (dd, *J* = 11.9, 1.1 Hz, 1H), 4.31 (td, *J* = 8.4, 4.3 Hz, 1H), 4.08 (q, *J* = 8.0 Hz, 1H), 3.84–3.73 (m, 2H), 3.37 (tt, *J* = 7.3, 1.3 Hz, 1H), 2.78 (td, *J* = 9.4, 5.8 Hz, 1H), 2.50 (dtd, *J* = 12.7, 7.5, 4.3 Hz, 1H), 2.28 (dtd, *J* = 12.8, 8.3, 7.4 Hz, 1H), 2.05 (dddd, *J* = 12.9, 9.3, 6.7, 5.4 Hz, 1H), 1.79–1.60 (m, 1H). ^13^C NMR (101 MHz, CDCl_3_) δ 209.1, 152.9, 135.4, 132.4, 129.1, 128.4, 127.7, 127.4, 125.3, 121.0, 116.2, 104.9, 101.6, 84.9, 78.7, 67.9, 66.8, 49.5, 49.1, 33.1, 27.0. HRMS ESI: Calculated for C_23_H_22_ClO_3_ [M + H]^+^ 381.1257, found 381.1255 (Diff.: −0.64 ppm).

#### 4.3.10. Synthesis of (2S,3R,3a′R,9a′S)-3-(1-(4-(trifluoromethyl)phenyl)propa-1,2-dien-1-yl)-2′,3′,3a′,4,5,9a′-hexahydro-3H-spiro[furan-2,4′-furo[2,3-b]chromene] **4j**

Compound **4j** was prepared following the general procedure using compound **1j** (50 mg, 0.145 mmol, 1 eq.), dihydrofuran (102 mg, 1.45 mmol, 10 eq.) and catalyst **A** (6 mg, 0.007 mmol, 0.05 eq.) in 1,4-dioxane (1 mL). Purification by chromatography on silica gel (8/2 pentane/Et_2_O) afforded compound **4j** (44%, 26 mg, 0.064 mmol) as an amorphous yellow solid. ^1^H NMR (400 MHz, CDCl_3_) δ 7.42–7.36 (m, 2H), 7.30–7.23 (m, 1H), 7.19 (d, *J* = 8.1 Hz, 2H), 7.03 (ddd, *J* = 8.0, 7.3, 1.7 Hz, 1H), 6.87 (td, *J* = 7.5, 1.2 Hz, 1H), 6.53 (dd, *J* = 8.1, 1.2 Hz, 1H), 5.43 (d, *J* = 6.0 Hz, 1H), 5.02–4.82 (m, 1H), 4.62 (d, *J* = 12.1 Hz, 1H), 4.31 (td, *J* = 8.4, 4.7 Hz, 1H), 4.10 (q, *J* = 8.0 Hz, 1H), 3.91–3.68 (m, 2H), 3.57–3.35 (m, 1H), 2.82 (td, *J* = 9.4, 5.9 Hz, 1H), 2.54 (dtd, *J* = 12.6, 7.7, 4.7 Hz, 1H), 2.44–2.23 (m, 1H), 2.13–1.97 (m, 1H), 1.69 (ddt, *J* = 12.9, 9.5, 8.4 Hz, 1H). ^13^C NMR (101 MHz, CDCl_3_) δ 209.6, 152.9, 140.7, 129.2, 128.7 (q, *J* = 32.4 Hz), 127.3, 126.6, 125.4, 125.1 (q, *J* = 3.8 Hz), 124.4 (q, *J* = 273.7 Hz), 121.1, 116.3, 105.3, 101.7, 85.0, 79.0, 67.9, 66.7, 49.4, 48.7, 33.0, 27.0. ^19^F NMR (376 MHz, CDCl_3_) δ–62.48 (s). HRMS ESI: Calculated for C_24_H_21_F_3_NaO_3_ [M + Na]^+^ 437.1340, found 437.1336 (Diff.: −0.31 ppm).

#### 4.3.11. Synthesis of (2S,3R,3a′R,9a′S)-3-(1-(4-nitrophenyl)propa-1,2-dien-1-yl)-2′,3′,3a′,4,5,9a′-hexahydro-3H-spiro[furan-2,4′-furo[2,3-b]chromene] **4k**

Compound **4k** was prepared following the general procedure using compound **1k** (50 mg, 0.205 mmol, 1 eq.), dihydrofuran (167 mg, 2.05 mmol, 10 eq.) and catalyst **A** (9 mg, 0.012 mmol, 0.05 eq.) in 1,4-dioxane (1 mL). Purification by chromatography on silica gel (8/2 pentane/Et_2_O) afforded compound **4k** (60%, 48 mg, 0.12 mmol) as an orange oil. ^1^H NMR (400 MHz, CDCl_3_) δ 7.98 (d, *J* = 9.0 Hz, 2H), 7.26 (dd, *J* = 7.5, 1.7 Hz, 1H), 7.20 (d, *J* = 8.9 Hz, 2H), 7.06–6.97 (m, 1H), 6.86 (td, *J* = 7.5, 1.2 Hz, 1H), 6.49 (dd, *J* = 8.0, 1.2 Hz, 1H), 5.52 (d, *J* = 6.1 Hz, 1H), 5.04 (dd, *J* = 12.7, 1.6 Hz, 1H), 4.79 (dd, *J* = 12.7, 1.2 Hz, 1H), 4.31 (td, *J* = 8.5, 5.3 Hz, 1H), 4.09 (td, *J* = 8.4, 6.9 Hz, 1H), 3.77 (ddd, *J* = 8.5, 6.2, 3.4 Hz, 2H), 3.63–3.45 (m, 1H), 2.88 (td, *J* = 9.3, 6.1 Hz, 1H), 2.59 (dtd, *J* = 13.1, 8.0, 5.3 Hz, 1H), 2.40–2.28 (m, 1H), 2.04 (dddd, *J* = 12.6, 9.4, 7.2, 5.2 Hz, 1H), 1.69 (dq, *J* = 13.1, 8.6 Hz, 1H). ^13^C NMR (101 MHz, CDCl_3_) δ 210.0, 152.7, 146.2, 143.7, 129.2, 127.2, 126.9, 125.6, 123.3, 121.3, 116.3, 105.8, 101.8, 84.8, 79.6, 67.9, 66.4, 49.1, 47.9, 32.7, 26.9. HRMS ESI: Calculated for C_23_H_21_NNaO_5_ [M + Na]^+^ 414.1317, found 414.1309 (Diff.: 0.35 ppm).

#### 4.3.12. Synthesis of (2S,3R,3a′R,9a′S)-3-(1-(3-nitrophenyl)propa-1,2-dien-1-yl)-2′,3′,3a′,4,5,9a′-hexahydro-3H-spiro[furan-2,4′-furo[2,3-b]chromene] **4l**

Compound **4l** was prepared following the general procedure using compound **1l** (51 mg, 0.159 mmol, 1 eq.), dihydrofuran (111 mg, 1.59 mmol, 10 eq.) and catalyst **A** (8 mg, 0.010 mmol, 0.05 eq.) in 1,4-dioxane (1 mL). Purification by chromatography on silica gel (8/2 pentane/Et_2_O) afforded compound **4l** (34%, 21 mg, 0.054 mmol) as an orange oil. ^1^H NMR (400 MHz, CDCl_3_) δ 7.90 (ddd, *J* = 8.0, 2.3, 1.2 Hz, 1H), 7.81 (t, *J* = 2.0 Hz, 1H), 7.34 (dt, *J* = 7.8, 1.5 Hz, 1H), 7.31–7.23 (m, 2H), 7.04–6.92 (m, 1H), 6.87 (td, *J* = 7.4, 1.3 Hz, 1H), 6.42 (dd, *J* = 8.0, 1.3 Hz, 1H), 5.53 (d, *J* = 6.0 Hz, 1H), 5.03 (dd, *J* = 12.2, 1.7 Hz, 1H), 4.83 (dd, *J* = 12.3, 1.4 Hz, 1H), 4.30 (td, *J* = 8.4, 5.5 Hz, 1H), 4.10 (td, *J* = 8.4, 6.8 Hz, 1H), 3.84–3.70 (m, 2H), 3.51 (ddt, *J* = 7.2, 5.4, 1.6 Hz, 1H), 2.88 (td, *J* = 9.3, 6.0 Hz, 1H), 2.68–2.50 (m, 1H), 2.43–2.28 (m, 1H), 2.04 (dddd, *J* = 12.7, 9.4, 7.3, 5.0 Hz, 1H), 1.76–1.60 (m, 1H). ^13^C NMR (101 MHz, CDCl_3_) δ 209.2, 152.8, 148.1, 138.7, 132.3, 129.1, 128.8, 127.1, 125.6, 125.5, 121.4, 121.3, 116.2, 105.5, 101.8, 84.9, 79.7, 68.0, 66.5, 49.1, 48.3, 32.6, 26.9. HRMS ESI: Calculated for C_23_H_21_NNaO_5_ [M + Na]^+^ 414.1317, found 414.1296 (Diff.: 4.40 ppm).

#### 4.3.13. Synthesis of (2S,3R,3a′R,9a′S)-3-(1-(thiophen-2-yl)propa-1,2-dien-1-yl)-2′,3′,3a′,4,5,9a′-hexahydro-3H-spiro[furan-2,4′-furo[2,3-b]chromene] **4m**

Compound **4m** was prepared following the general procedure using compound **1m** (50 mg, 0.177 mmol, 1 eq.), dihydrofuran (124 mg, 1.77 mmol, 10 eq.) and catalyst **A** (7 mg, 0.09 mmol, 0.05 eq.) in 1,4-dioxane (1 mL). Purification by chromatography on silica gel (8/2 pentane/Et_2_O) afforded compound **4m** (65%, 41 mg, 0.117 mmol) as an amorphous yellow solid. ^1^H NMR (400 MHz, CDCl_3_) δ 7.24 (dd, *J* = 7.6, 1.7 Hz, 1H), 7.15–7.05 (m, 2H), 6.93–6.79 (m, 3H), 6.66 (dd, *J* = 8.1, 1.2 Hz, 1H), 5.54 (d, *J* = 5.7 Hz, 1H), 4.89 (d, *J* = 12.3 Hz, 1H), 4.48 (dd, *J* = 12.3, 0.9 Hz, 1H), 4.30 (td, *J* = 8.5, 3.9 Hz, 1H), 4.14–4.00 (m, 1H), 3.92–3.75 (m, 2H), 3.42–3.25 (m, 1H), 2.83 (td, *J* = 9.5, 5.7 Hz, 1H), 2.47 (dtd, *J* = 12.8, 7.4, 3.9 Hz, 1H), 2.25 (dq, *J* = 12.8, 8.4 Hz, 1H), 2.08 (dddd, *J* = 12.8, 9.2, 7.2, 4.9 Hz, 1H), 1.72 (ddt, *J* = 12.9, 9.8, 8.5 Hz, 1H).^13^C NMR (101 MHz, CDCl_3_) δ 208.4, 153.0, 141.3, 129.1, 127.5, 127.3, 124.9, 124.5, 123.0, 120.8, 116.1, 101.7, 100.8, 84.9, 79.5, 67.8, 66.8, 51.1, 49.6, 32.9, 27.1. HRMS ESI: Calculated for C_21_H_21_SO_3_ [M + H]^+^ 353.1211, found 353.1196 (Diff.: 2.77 ppm).

#### 4.3.14. Synthesis of (2S,3R,3a′R,9a′S)-3-(1-(benzo[b]thiophen-2-yl)propa-1,2-dien-1-yl)-2′,3′,3a′,4,5,9a′-hexahydro-3H-spiro[furan-2,4′-furo[2,3-b]chromene] **4n**

Compound **4i** was prepared following the general procedure using compound **1i** (50 mg, 0.150 mmol, 1 eq.), dihydrofuran (105 mg, 1.50 mmol, 10 eq.) and catalyst **A** (6 mg, 0.008 mmol, 0.05 eq.) in 1,4-dioxane (1 mL). Purification by chromatography on silica gel (8/2 pentane/Et_2_O) afforded compound **4i** (68%, 41 mg, 0.102 mmol) as an orange oil. ^1^H NMR (400 MHz, CDCl_3_) δ 7.7–7.6 (m, 2H), 7.3–7.2 (m, 3H), 7.2–7.0 (m, 2H), 6.9 (td, *J* = 7.5, 1.2 Hz, 1H), 6.7 (dd, *J* = 8.0, 1.2 Hz, 1H), 5.6 (d, *J* = 5.8 Hz, 1H), 5.0 (dt, *J* = 12.8, 1.1 Hz, 1H), 4.5 (dt, *J* = 12.8, 1.0 Hz, 1H), 4.3 (td, *J* = 8.5, 3.6 Hz, 1H), 4.1 (td, *J* = 8.6, 7.5 Hz, 1H), 4.0–3.7 (m, 2H), 3.4 (dd, *J* = 8.4, 7.1 Hz, 1H), 2.9 (td, *J* = 9.4, 5.8 Hz, 1H), 2.5 (dtd, *J* = 12.8, 7.3, 3.6 Hz, 1H), 2.4–2.2 (m, 1H), 2.2–2.0 (m, 1H), 1.7 (ddt, *J* = 12.9, 9.7, 8.4 Hz, 1H). ^13^C NMR (101 MHz, CDCl_3_) δ 209.3, 153.0, 141.9, 140.4, 139.4, 129.2, 127.5, 125.2, 124.3, 124.2, 123.3, 122.0, 121.0, 119.2, 116.3, 101.9, 101.4, 85.0, 80.0, 67.8, 66.8, 50.1, 49.7, 33.0, 27.2. HRMS ESI: Calculated for C_25_H_23_SO_3_ [M + H]^+^ 403.1368, found 403.1354 (Diff.: 1.64 ppm).

Procedures for the synthesis of the substrates and the products and full characterization of final products can be found in the Supplementary Materials.

## Figures and Tables

**Table 1:** Optimization of the reaction conditions. ^1^

Entry	Dienophile	Product	Yield (%)
1	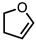	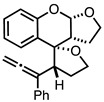	**4a** 64%
2	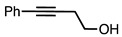	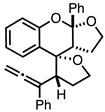	**5a** 7%
3	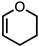	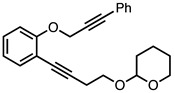	**6** 20%
4	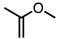	CM ^2^	-
5	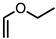	CM ^2^	-
6	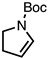	CM ^2^	-
7	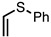	CM ^2^	-
8	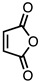	CM ^2^	-

^1^ The reactions were performed by adding **Cat. A** (5 mol%) to a solution of **1a** (1equiv.) and dienophile (10 equiv.) in 1,4-dioxane (0.145 M). ^2^ CM: Complex Mixture.

## Supplementary Materials

The supplementary materials are available online.
