# Supplementary material for: Gold(I)-Catalyzed Domino Reaction for Furopyrans Synthesis
Source: Molecules. 2020 Oct 27;25(21):4976. doi: 10.3390/molecules25214976 (PMC7663467; doi:10.3390/molecules25214976)

Gold(I)-Catalyzed Domino Reaction for Furopyrans Synthesis.

Marie Ruch, Nicolas Brach, Romeric Galea, Patrick Wagner, Gaëlle Blond*

Université de Strasbourg, CNRS, Laboratoire d'Innovation Thérapeutique, UMR 7200, 67000 Strasbourg, gaelle.blond@unistra.fr; Tel.: +33-368854165

Table of contents

1. General information **2**

2. Synthesis of 1f and 1g **2**
2.1 Synthesis of **1f** **3**
2.2 Synthesis of **1g** **3**

3. General procedure for gold(I) catalyzed cascade reactions: preparation of 4a-n **4**
3.1 Synthesis of **4a** **4**
3.2 Synthesis of **4b** **4**
3.3 Synthesis of **4c** **5**
3.4 Synthesis of **4d** **6**
3.5 Synthesis of **4e** **6**
3.6 Synthesis of **4f** **7**
3.7 Synthesis of **4g** **7**
3.8 Synthesis of **4h** **8**
3.9 Synthesis of **4i** **8**
3.10 Synthesis of **4j** **9**
3.11 Synthesis of **4k** **9**
3.12 Synthesis of **4l** **10**
3.13 Synthesis of **4m** **11**
3.14 Synthesis of **4n** **11**

4. 1H and 13C NMR spectra **12**

1. General information

All reagents, chemicals and dry solvents were purchased from commercial sources and used without purification. When mentioned that the reaction was conducted in dry media, glassware dried for several hours at 110 °C in an oven was used. Triethylamine (Et_3N) and diisopropylamine (DIPA) were distilled from KOH in an S-tube prior to each experiment in which they were involved. Reactions were monitored by TLC (Thin Layer silica gel Chromatography) using Merck silica gel 60 F254 on aluminum sheets. TLC plates were visualized under UV light and revealed with acidic *p*-anisaldehyde stain or $KMnO_4$ stain. Crude products were purified by flash column chromatography on Merck silica gel Si 60 (40-63 µm). NMR spectra were recorded in $CDCl_3$ on a Bruker Avance III BBFO+ probe spectrometer 400 MHz for ^1H analyses and 100 MHz for ^{13}C analyses. Proton chemical shifts are reported in ppm (δ), relatively to residual $CHCl_3$ (δ 7.26 ppm). Multiplicities are reported as follows: singlet (s), doublet (d), triplet (t), quartet (q), quintet (quint), broad singlet (bs), broad doublet (bd) combinations or multiplet (m). Coupling constants values J are given in Hz. Carbon chemical shifts are reported in ppm (δ), relatively to the internal standard $CDCl_3$ (δ 77.23 ppm). ^1H and ^{13}C NMR signals were assigned mostly on the basis of 2D-NMR (COSY, HSQC, HMBC) experiments. High Resolution Mass Spectral analyses (HRMS) were performed using an Agilent 1200 RRLC HPLC chain and an Agilent 6520 Accurate mass QToF. Infrared spectra (IR) were recorded on a FT IR Thermo Nicollet ATR 380, Diamant Spectrometer.
All compounds **1a-e** and **1h-n** have been already described.[1, 2]

2. Synthesis of 1f and 1g

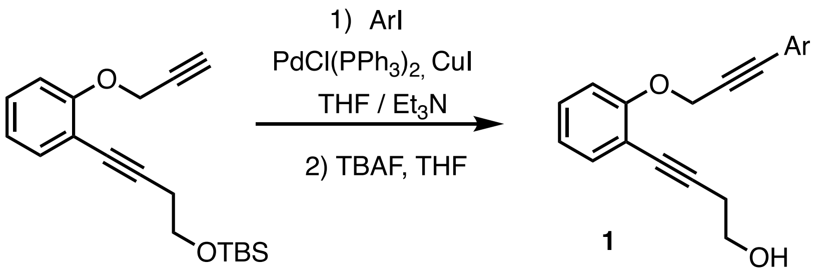

1

Anhydrous THF and distilled Et_3N are mixed in a 2-necked flask under argon. The iodoaryl (1.5 eq.), $PdCl_2(PPh_3)$ (0,03 eq.) and CuI (0,06 eq.) were added to the flask and this mixture was degassed with argon for 15 min. The alkyne (1 eq.) was dissolved in THF degassed with argon for 15 min and added to the 2-necked flask. The mixture was stirred overnight at room temperature (20 °C) and monitored by TLC (9/1 pent/Et_2O). Once the TLC showed complete conversion of the true alkyne, the reaction mixture was filtered through a pad of Celite with CH_2Cl_2 as eluent and concentrated to give the crude product as a dark-brown solid. The latter was purified by flash column chromatography (98/2 pent/Et_2O) to afford the pure TBS protected coupling product.
TBAF (1 eq.) was added to a solution of this latter protected product in THF at 0 °C. This mixture was stirred at room temperature until the TLC (9/1 pent/Et2O) showed complete conversion of the starting material. The reaction mixture was dissolved in a saturated aqueous NH_4Cl solution. The aqueous phase was extracted with CH_2Cl_2. The gathered organic layer

[1] Pertschi, R.; Wagner, P.; Ghosh, N.; Gandon, V.; Blond, G. *Org. Lett.* **2019**, *21*, 6084–6088
[2] Wagner, P.; Ghosh, N.; Gandon, V.; Blond, G. *Org. Lett.* **2020**, *22*, 7333-7337

were dried over $MgSO_4$, filtered and concentrated to afford the crude as a yellowish oil. The latter was purified by flash column chromatography (6/4 pent/Et_2O) to afford the pure deprotected compound (**1b-x**).

2.1 Synthesis of **1f**

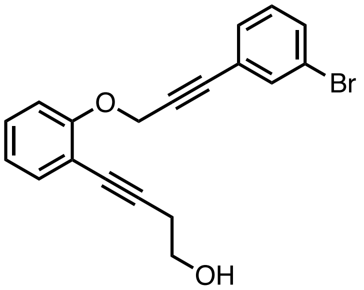

Compound **1f** was prepared following the general procedure using compound **15** (500 mg, 1.6 mmol, 1 eq.), THF (10 mL), $PdCl_2(PPh_3)_2$ (34 mg, 0.05 mmol, 0.03 eq.), CuI (18 mg, 0,1 mmol, 0.06 eq.), 3-bromoiodobenzene (679 mg, 2.4 mmol, 1.5 eq.), Et_3N (4 mL) and TBAF (1.6 mL, 1.60 mmol, 1 eq.) in THF (10 mL) for the deprotection. Purification by chromatography on silica gel afforded compound **1f** (81 %, 499 mg, 1.405 mmol in two steps) of as an orange oil.

^{1}H NMR (400 MHz, $CDCl_3$) δ 7.67 (t, *J* = 1.7 Hz, 1H), 7.55 (ddd, *J* = 8.0, 2.1, 1.1 Hz, 1H), 7.50 (dd, *J* = 7.6, 1.7 Hz, 1H), 7.45 (dt, *J* = 7.8, 1.3 Hz, 1H), 7.38 (ddd, *J* = 8.4, 7.5, 1.8 Hz, 1H), 7.26 (t, *J* = 7.9 Hz, 1H), 7.15 (dd, *J* = 8.4, 1.1 Hz, 1H), 7.05 (td, *J* = 7.5, 1.0 Hz, 1H), 5.07 (s, 2H), 3.92 (q, *J* = 5.2 Hz, 2H), 2.84 (t, *J* = 6.1 Hz, 2H), 2.35 (s, 1H). **^{13}C NMR** (101 MHz, $CDCl_3$) δ 158.3, 134.6, 133.6, 132.0, 130.5, 129.9, 129.3, 124.3, 122.2, 121.6, 113.5, 112.9, 91.3, 86.1, 85.1, 78.9, 61.2, 57.4, 24.4. **HRMS** ESI: Calculated for $C_{19}H_{15}BrO_2$ $[M+H]^+$ 355.0334, found 355.0311 (Diff.: 4.58 ppm).

2.2 Synthesis of **1g**

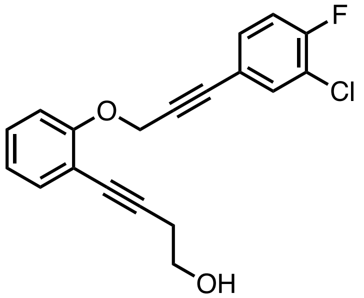

Compound **1g** was prepared following the general procedure using compound **15** (500 mg, 1.6 mmol, 1 eq.), THF (10 mL), $PdCl_2(PPh_3)_2$ (34 mg, 0.05 mmol, 0.03 eq.), CuI (18 mg, 0,1 mmol, 0.06 eq.), 3-chloro-4-chloroiodobenzene (615 mg, 2.4 mmol, 1.5 eq.), Et_3N (4 mL) and TBAF (0.86 mL, 0.86 mmol, 1 eq.) in THF (10 mL) for the deprotection. Purification by chromatography on silica gel afforded compound **1g** (72 %, 380 mg, 1.15 mmol in two steps) of as an orange oil.

^{1}H NMR (400 MHz, $CDCl_3$) δ 7.67 (dd, *J* = 7.0, 2.1 Hz, 1H), 7.60 (dd, *J* = 7.6, 1.7 Hz, 1H), 7.54 – 7.44 (m, 2H), 7.30 – 7.20 (m, 2H), 7.15 (td, *J* = 7.5, 1.0 Hz, 1H), 5.15 (s, 2H), 4.02 (t, *J* = 6.1 Hz,

2H), 2.94 (t, *J* = 6.1 Hz, 2H), 2.49 (s, 1H). **^{13}C NMR** (101 MHz, $CDCl_3$) δ 158.4 (d, *J* = 252.5 Hz), 158.3, 134.1, 133.6, 131.9 (d, *J* = 7.0 Hz), 129.3, 121.6, 121.3 (d, *J* = 18.0 Hz), 119.5 (d, *J* = 4.0 Hz), 116.8 (d, *J* = 21.0 Hz), 113.5, 112.9, 91.3, 85.4, 84.6, 78.9, 61.1, 57.3, 24.3. **HRMS** ESI: Calculated for $C_{19}H_{14}ClFO_2$ $[M+H]^+$ 329.0745, found 329.0723 (Diff.: 4.91 ppm).

3. General procedure for gold(I) catalyzed cascade reactions: preparation of 4a-n

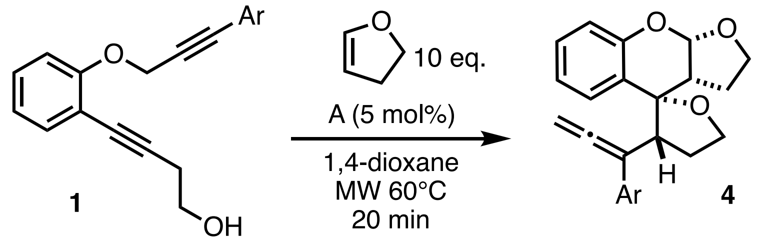

Substrate **1** (1 eq.) and dihydrofuran (10 eq.) was placed in a 0.5-2 mL microwave reactor and dissolved in anhydrous 1,4-dioxane. Catalyst **A** (0.05 eq.) was added into the reactor. Once all the reagents were dissolved, the reactor was placed into the microwave for 20 min at 60 °C. The reaction mixture was filtered through a pad of Celite with CH_2Cl_2 as eluent. After solvent evaporation under reduced pressure, purification of the crude by flash column chromatography provided the furopyran adduct **4**.

3.1. Synthesis of **4a**

(2*S*,3*R*,3a'*R*,9a'*S*)-3-(1-phenylpropa-1,2-dien-1-yl)-2',3',3a',4,5,9a'-hexahydro-3*H*-spiro[furan-2,4'-furo[2,3-*b*]chromene]

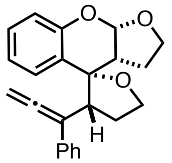

Compound **4a** was prepared following the general procedure using compound **1a** (20 mg, 0.072 mmol, 1 eq.), dihydrofuran (51 mg, 0.72 mmol, 10 eq.) and catalyst **A** (3 mg, 0,004 mmol, 0.05 eq.) in 1,4-dioxane (0.4 mL). Purification by chromatography on silica gel (8/2 pentane/ Et_2O) afforded compound **4a** (64 %, 16 mg, 0.046 mmol) as an amorphous white solid.

^{1}H NMR (400 MHz, $CDCl_3$) δ 7.28 – 7.24 (m, 1H), 7.23 – 7.11 (m, 5H), 7.11 – 7.05 (m, 1H), 6.89 (td, *J* = 7.5, 1.2 Hz, 1H), 6.62 (dd, *J* = 8.1, 1.2 Hz, 1H), 5.31 (d, *J* = 5.6 Hz, 1H), 4.81 (dd, *J* = 11.7, 1.3 Hz, 1H), 4.42 (d, *J* = 11.7 Hz, 1H), 4.33 (td, *J* = 8.4, 3.8 Hz, 1H), 4.10 (td, *J* = 8.4, 7.3 Hz, 1H), 3.87 – 3.73 (m, 2H), 3.48 – 3.37 (m, 1H), 2.75 (td, *J* = 9.4, 5.6 Hz, 1H), 2.48 (dtd, *J* = 12.8, 7.3, 3.8 Hz, 1H), 2.30 (dq, *J* = 12.7, 8.5 Hz, 1H), 2.13 – 1.99 (m, 1H), 1.70 (dtd, *J* = 12.9, 9.6, 8.4 Hz, 1H). **^{13}C NMR** (101 MHz, $CDCl_3$) δ 209.3, 153.0, 137.0, 129.0, 128.4, 127.5, 126.8, 126.5, 125.2, 120.8, 116.0, 105.1, 101.6, 85.0, 78.3, 67.8, 67.0, 49.7, 49.6, 33.3, 27.0. **HRMS** ESI: Calculated for $C_{23}H_{23}O_3$ $[M+H]^+$ 347.1647, found 347.1642 (Diff.: 0.31 ppm).

3.2 Synthesis of **4b**

(2*S*,3*R*,3a'*R*,9a'*S*)-3-(1-(*p*-tolyl)propa-1,2-dien-1-yl)-2',3',3a',4,5,9a'-hexahydro-3*H*-spiro[furan-2,4'-furo[2,3-*b*]chromene]

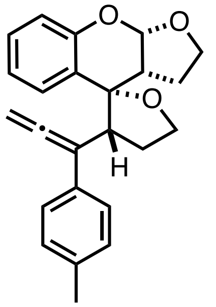

Compound **4b** was prepared following the general procedure using compound **1b** (50 mg, 0.172 mmol, 1 eq.), dihydrofuran (120 mg, 1.72 mmol, 10 eq.) and catalyst **A** (7 mg, 0,009 mmol, 0.05 eq.) in 1,4-dioxane (0.4 mL). Purification by chromatography on silica gel (8/2 pentane/ Et_2O) afforded compound **4b** (80 %, 50 mg, 0.138 mmol) as an orange oil.

^{1}H NMR (400 MHz, $CDCl_3$) δ 7.24 (dd, *J* = 7.7, 1.6 Hz, 1H), 7.10 (td, *J* = 7.7, 1.7 Hz, 1H), 7.05 (d, *J* = 8.4 Hz, 2H), 7.01 (d, *J* = 8.4 Hz, 2H), 6.90 (dd, *J* = 7.5, 1.2 Hz, 1H), 6.64 (dd, *J* = 8.1, 1.2 Hz, 1H), 5.25 (d, *J* = 5.7 Hz, 1H), 4.76 (d, *J* = 11.5 Hz, 1H), 4.32 (td, *J* = 8.8, 3.6 Hz, 2H), 4.09 (td, *J* = 8.7, 7.2 Hz, 1H), 3.88 – 3.70 (m, 2H), 3.38 (dd, *J* = 8.9, 7.1 Hz, 1H), 2.72 (td, *J* = 9.5, 5.6 Hz, 1H), 2.44 (dtd, *J* = 12.7, 7.1, 3.5 Hz, 1H), 2.38 – 2.22 (m, 4H), 2.15 – 1.99 (m, 1H), 1.70 (dq, *J* = 12.8, 8.9 Hz, 1H). **^{13}C NMR** (101 MHz, $CDCl_3$) δ 209.1, 153.0, 136.5, 134.1, 129.1, 129.0, 127.6, 126.3, 125.2, 120.7, 116.0, 104.6, 101.5, 85.0, 78.1, 67.7, 67.0, 49.9, 49.8, 33.3, 27.0, 21.1. **HRMS** ESI: Calculated for $C_{24}H_{25}O_3$ $[M+H]^+$ 361.1804, found 361.1793 (Diff.: 1.59 ppm).

3.3 Synthesis of **4c**

(2*S*,3*R*,3a'*R*,9a'*S*)-3-(1-(3-methoxyphenyl)propa-1,2-dien-1-yl)-2',3',3a',4,5,9a'-hexahydro-3*H*-spiro[furan-2,4'-furo[2,3-*b*]chromene]

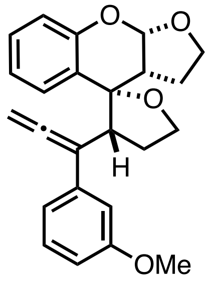

Compound **4c** was prepared following the general procedure using compound **1c** (51 mg, 0.167 mmol, 1 eq.), dihydrofuran (117 mg, 1.67 mmol, 10 eq.) and catalyst **A** (6 mg, 0,008 mmol, 0.05 eq.) in 1,4-dioxane (1 mL). Purification by chromatography on silica gel (8/2 pentane/ Et_2O) afforded compound **4c** (61 %, 39 mg, 0.102 mmol) as an amorphous yellow solid.

^{1}H NMR (400 MHz, $CDCl_3$) δ 7.29 – 7.20 (m, 1H), 7.16 – 7.05 (m, 2H), 6.88 (td, *J* = 7.5, 1.2 Hz, 1H), 6.78 (dt, *J* = 7.9, 1.2 Hz, 1H), 6.70 – 6.59 (m, 3H), 5.35 (d, *J* = 5.7 Hz, 1H), 4.81 (dd, *J* = 11.7, 1.4 Hz, 1H), 4.52 – 4.37 (m, 1H), 4.31 (td, *J* = 8.4, 3.9 Hz, 1H), 4.08 (td, *J* = 8.4, 7.4 Hz, 1H), 3.94 – 3.65 (m, 5H), 3.47 – 3.26 (m, 1H), 2.75 (td, *J* = 9.5, 5.7 Hz, 1H), 2.46 (dtd, *J* = 12.7, 7.3, 3.9 Hz, 1H), 2.28 (dq, *J* = 12.6, 8.4 Hz, 1H), 2.05 (dddd, *J* = 12.9, 9.2, 7.2, 4.8 Hz, 1H), 1.69 (ddt, *J* = 12.9, 9.9, 8.7 Hz, 1H). **^{13}C NMR** (101 MHz, $CDCl_3$) δ 209.2, 159.6, 153.0, 138.5, 129.2, 129.0, 127.5, 125.1, 120.7, 118.8, 116.0, 112.4, 112.3, 105.2, 101.6, 84.9, 78.3, 67.8, 66.9, 55.2, 49.6, 49.6, 33.3, 27.0. **HRMS** ESI: Calculated for $C_{24}H_{25}O_4$ $[M+H]^+$ 377.1753, found 377.1750 (Diff.: -0.74 ppm).

3.4 Synthesis of **4d**

(2*S*,3*R*,3a'*R*,9a'*S*)-3-(1-(4-methoxyphenyl)propa-1,2-dien-1-yl)-2',3',3a',4,5,9a'-hexahydro-3*H*-spiro[furan-2,4'-furo[2,3-*b*]chromene]

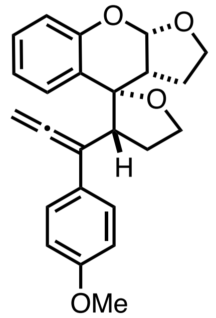

Compound **4d** was prepared following the general procedure using compound **1d** (49 mg, 0.160 mmol, 1 eq.), dihydrofuran (112 mg, 1.60 mmol, 10 eq.) and catalyst **A** (7 mg, 0,009 mmol, 0.05 eq.) in 1,4-dioxane (1 mL). Purification by chromatography on silica gel (8/2 pentane/ Et_2O) afforded compound **4d** (60 %, 37 mg, 0.098 mmol) as a yellow oil.

^{1}H NMR (400 MHz, $CDCl_3$) δ 7.24 (dd, *J* = 7.6, 1.7 Hz, 1H), 7.12 – 7.03 (m, 3H), 6.88 (td, *J* = 7.5, 1.2 Hz, 1H), 6.79 – 6.70 (m, 2H), 6.63 (dd, *J* = 8.0, 1.2 Hz, 1H), 5.30 (d, *J* = 5.7 Hz, 1H), 4.78 (dd, *J* = 11.4, 1.3 Hz, 1H), 4.38 (dd, *J* = 11.4, 1.0 Hz, 1H), 4.32 (td, *J* = 8.4, 3.8 Hz, 1H), 4.08 (td, *J* = 8.5, 7.3 Hz, 1H), 3.85 – 3.72 (m, 6H), 3.40 – 3.32 (m, 1H), 2.74 (td, *J* = 9.5, 5.7 Hz, 1H), 2.46 (dtd, *J* = 12.8, 7.2, 3.7 Hz, 1H), 2.28 (dq, *J* = 12.8, 8.5 Hz, 1H), 2.06 (dddd, *J* = 12.9, 9.3, 7.3, 4.7 Hz, 1H), 1.85 – 1.66 (m, 1H). **^{13}C NMR** (101 MHz, $CDCl_3$) δ 208.9, 158.5, 152.9, 129.1, 128.9, 127.4, 127.4, 125.1, 120.6, 115.8, 113.7, 104.5, 101.4, 84.9, 78.0, 67.6, 66.8, 55.2, 49.8, 49.6, 33.1, 26.9. **HRMS** ESI: Calculated for $C_{24}H_{24}NaO_4$ $[M+Na]^+$ 399.1572, found 399.1551 (Diff.: 4.13 ppm).

3.5 Synthesis of **4e**

(2*S*,3*R*,3a'*R*,9a'*S*)-3-(1-(3-fluorophenyl)propa-1,2-dien-1-yl)-2',3',3a',4,5,9a'-hexahydro-3*H*-spiro[furan-2,4'-furo[2,3-*b*]chromene]

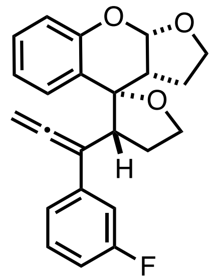

Compound **4e** was prepared following the general procedure using compound **1e** (50 mg, 0.170 mmol, 1 eq.), dihydrofuran (119 mg, 1.70 mmol, 10 eq.) and catalyst **A** (7 mg, 0,009 mmol, 0.05 eq.) in 1,4-dioxane (1 mL). Purification by chromatography on silica gel (8/2 pentane/ Et_2O) afforded compound **4e** (65 %, 41 mg, 0.11 mmol) as orange oil.

^{1}H NMR (400 MHz, $CDCl_3$) δ 7.28 – 7.22 (m, 1H), 7.18 – 7.05 (m, 2H), 6.96 – 6.86 (m, 2H), 6.85 – 6.75 (m, 2H), 6.60 (dd, *J* = 8.1, 1.2 Hz, 1H), 5.40 (d, *J* = 5.8 Hz, 1H), 4.87 (dd, *J* = 12.0, 1.3 Hz, 1H), 4.52 (d, *J* = 11.9 Hz, 1H), 4.31 (td, *J* = 8.4, 4.3 Hz, 1H), 4.09 (q, *J* = 8.1 Hz, 1H), 3.89 – 3.75 (m, 2H), 3.39 (dd, *J* = 8.1, 6.8 Hz, 1H), 2.79 (td, *J* = 9.4, 5.8 Hz, 1H), 2.50 (dtd, *J* = 12.8, 7.5, 4.2 Hz, 1H), 2.38 – 2.24 (m, 1H), 2.06 (dddd, *J* = 12.9, 9.3, 6.6, 5.5 Hz, 1H), 1.83 – 1.64 (m, 1H). **^{13}C**

NMR (101 MHz, $CDCl_3$) δ 209.2, 162.8 (d, J = 244.9 Hz), 152.9, 139.4 (d, J = 7.6 Hz), 129.6 (d, J = 8.4 Hz), 129.1, 127.4, 125.2, 121.8 (d, J = 2.7 Hz), 120.9, 116.1, 113.6 (d, J = 21.2 Hz), 113.5 (d, J = 23.2 Hz), 104.9, 101.6, 84.9, 78.8, 67.8, 66.8, 49.5, 49.1, 33.1, 27.0. ^{19}F NMR (376 MHz, $CDCl_3$) δ -113.54 – -113.72 (m). **HRMS** ESI: Calculated for $C_{23}H_{22}FO_3$ $[M+H]^+$ 365.1553, found 365.1546 (Diff.: 0.51 ppm).

3.6 Synthesis of **4f**

(2*S*,3*R*,3a'*R*,9a'*S*)-3-(1-(3-bromophenyl)propa-1,2-dien-1-yl)-2',3',3a',4,5,9a'-hexahydro-3*H*-spiro[furan-2,4'-furo[2,3-*b*]chromene]

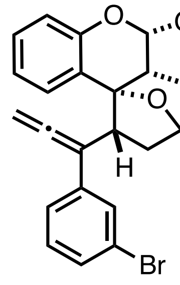

Compound **4f** was prepared following the general procedure using compound **1f** (53 mg, 0.149 mmol, 1 eq.), dihydrofuran (104 mg, 1.49 mmol, 10 eq.) and catalyst **A** (7 mg, 0,009 mmol, 0.05 eq.) in 1,4-dioxane (1 mL). Purification by chromatography on silica gel (8/2 pentane/ Et_2O) afforded compound **4f** (70 %, 45 mg, 0.104 mmol) as an amorphous yellow solid.

^{1}H NMR (400 MHz, $CDCl_3$) δ 7.24 (dd, J = 7.6, 1.7 Hz, 1H), 7.22 – 7.18 (m, 1H), 7.12 (q, J = 1.4 Hz, 1H), 7.10 – 7.00 (m, 3H), 6.89 (td, J = 7.5, 1.2 Hz, 1H), 6.57 (dd, J = 8.1, 1.2 Hz, 1H), 5.43 (d, J = 5.8 Hz, 1H), 4.89 (dd, J = 11.9, 1.5 Hz, 1H), 4.58 (dd, J = 12.0, 1.1 Hz, 1H), 4.29 (td, J = 8.4, 4.6 Hz, 1H), 3.78 (dd, J = 8.4, 6.1 Hz, 2H), 3.37 (tt, J = 7.2, 1.4 Hz, 1H), 2.79 (td, J = 9.4, 5.8 Hz, 1H), 2.55 – 2.42 (m, 1H), 2.27 (dtd, J = 12.8, 8.2, 7.0 Hz, 1H), 2.12 – 1.98 (m, 1H), 1.69 (ddt, J = 12.9, 9.7, 8.4 Hz, 1H). **^{13}C NMR** (101 MHz, $CDCl_3$) δ 209.1, 152.9, 139.1, 129.6 (3C), 129.1, 127.2, 125.1, 124.8, 122.4, 121.0, 116.1, 105.0, 101.6, 84.9, 78.9, 67.8, 66.7, 49.4, 48.9, 32.9, 26.9. **HRMS** ESI: Calculated for $C_{23}H_{21}BrNaO_3$ $[M+Na]^+$ 447.0572, found 447.0567 (Diff.: -0.49 ppm).

3.7 Synthesis of **4g**

(2*S*,3*R*,3a'*R*,9a'*S*)-3-(1-(3-chloro-4-fluorophenyl)propa-1,2-dien-1-yl)-2',3',3a',4,5,9a'-hexahydro-3*H*-spiro[furan-2,4'-furo[2,3-*b*]chromene]

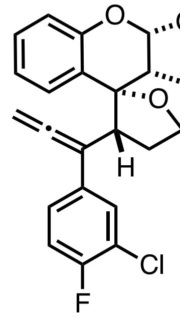

Compound **4g** was prepared following the general procedure using compound **1g** (52 mg, 0.16 mmol, 1 eq.), dihydrofuran (112 mg, 1.6 mmol, 10 eq.) and catalyst **A** (8 mg, 0,010 mmol, 0.05 eq.) in 1,4-dioxane (1 mL). Purification by chromatography on silica gel (8/2 pentane/ Et_2O) afforded compound **4g** (44 %, 28 mg, 0.070 mmol) as a yellow oil.

^{1}**H NMR** (400 MHz, $CDCl_3$) δ 7.27 – 7.21 (m, 1H), 7.05 (ddd, *J* = 8.1, 7.3, 1.7 Hz, 1H), 7.00 – 6.85 (m, 4H), 6.55 (dd, *J* = 8.0, 1.2 Hz, 1H), 5.48 (d, *J* = 5.9 Hz, 1H), 4.92 (dt, *J* = 11.9, 1.2 Hz, 1H), 4.66 (dt, *J* = 11.8, 1.2 Hz, 1H), 4.28 (td, *J* = 8.4, 5.1 Hz, 1H), 4.07 (td, *J* = 8.2, 7.1 Hz, 1H), 3.82 – 3.75 (m, 2H), 3.35 (ddt, *J* = 7.7, 6.2, 1.5 Hz, 1H), 2.82 (td, *J* = 9.4, 5.9 Hz, 1H), 2.52 (dtd, *J* = 12.8, 7.8, 5.1 Hz, 1H), 2.28 (dddd, *J* = 13.2, 8.4, 7.2, 6.1 Hz, 1H), 2.11 – 1.98 (m, 1H), 1.68 (ddt, *J* = 12.9, 9.5, 8.3 Hz, 1H). ^{13}**C NMR** (101 MHz, $CDCl_3$) δ 208.9, 156.9 (d, *J* = 256.6 Hz), 152.8, 134.0 (d, *J* = 3.3 Hz), 129.1, 128.7, 127.1, 125.9 (d, *J* = 7.1 Hz), 125.3, 121.1, 120.6 (d, *J* = 17.8 Hz), 116.1, 116.0 (d, *J* = 21.1 Hz), 104.8, 101.7, 84.8, 79.1, 67.9, 66.5, 49.2, 48.9, 32.8, 26.9. **HRMS** ESI: Calculated for $C_{23}H_{21}ClFO_3$ $[M+H]^+$ 399.1163, found 399.1165 (Diff.: -0.61 ppm).

3.8 Synthesis of **4h**

(2*S*,3*R*,3a'*R*,9a'*S*)-3-(1-(4-bromo-3-fluorophenyl)propa-1,2-dien-1-yl)-2',3',3a',4,5,9a'-hexahydro-3*H*-spiro[furan-2,4'-furo[2,3-*b*]chromene]

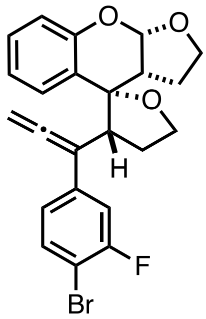

Compound **4h** was prepared following the general procedure using compound **1h** (50 mg, 0.152 mmol, 1 eq.), dihydrofuran (106 mg, 1.52 mmol, 10 eq.) and catalyst **A** (6 mg, 0,008 mmol, 0.05 eq.) in 1,4-dioxane (1 mL). Purification by chromatography on silica gel (8/2 pentane/ Et_2O) afforded compound **4h** (46 %, 24 mg, 0.070 mmol) as a yellow oil.

^{1}**H NMR** (400 MHz, $CDCl_3$) δ 7.32 (dd, *J* = 8.6, 7.3 Hz, 1H), 7.24 (dd, *J* = 7.5, 1.8 Hz, 1H), 7.07 (ddd, *J* = 8.1, 7.3, 1.7 Hz, 1H), 6.88 (td, *J* = 7.5, 1.2 Hz, 1H), 6.84 – 6.75 (m, 2H), 6.58 (dd, *J* = 8.1, 1.2 Hz, 1H), 5.45 (d, *J* = 5.9 Hz, 1H), 4.91 (dd, *J* = 12.1, 1.4 Hz, 1H), 4.59 (dd, *J* = 12.2, 1.0 Hz, 1H), 4.30 (td, *J* = 8.4, 4.7 Hz, 1H), 4.08 (q, *J* = 7.9 Hz, 1H), 3.78 (dd, *J* = 8.4, 6.1 Hz, 2H), 3.35 (t, *J* = 7.1 Hz, 1H), 2.81 (td, *J* = 9.3, 5.9 Hz, 1H), 2.51 (dtd, *J* = 12.5, 7.7, 4.7 Hz, 1H), 2.27 (dq, *J* = 12.9, 7.6 Hz, 1H), 2.05 (ddt, *J* = 12.7, 9.4, 6.1 Hz, 1H), 1.69 (dq, *J* = 13.0, 8.5 Hz, 1H). ^{13}**C NMR** (101 MHz, $CDCl_3$) δ 209.1, 158.8 (d, *J* = 246.4 Hz), 152.9, 138.6 (d, *J* = 6.9 Hz), 132.9 (d, *J* = 1.0 Hz), 129.2, 127.3, 125.4, 122.9 (d, *J* = 3.2 Hz), 121.1, 116.2, 114.5 (d, *J* = 23.5 Hz), 106.8 (d, *J* = 21.2 Hz), 104.86 (d, *J* = 2.2 Hz), 101.7, 84.9, 79.3, 67.9, 66.6, 49.4, 48.7, 32.9, 27.0. **HRMS** ESI: Calculated for $C_{23}H_{20}BrFNaO_3$ $[M+Na]^+$ 465.0478, found 465.0464 (Diff.: 0.65 ppm).

3.9 Synthesis of **4i**

(2*S*,3*R*,3a'*R*,9a'*S*)-3-(1-(4-chlorophenyl)propa-1,2-dien-1-yl)-2',3',3a',4,5,9a'-hexahydro-3*H*-spiro[furan-2,4'-furo[2,3-*b*]chromene]

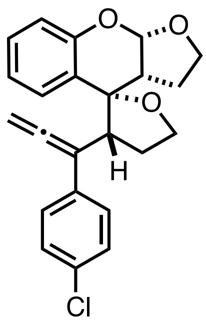

Compound **4i** was prepared following the general procedure using compound **1i** (50 mg, 0.161 mmol, 1 eq.), dihydrofuran (113 mg, 1.61 mmol, 10 eq.) and catalyst **A** (8 mg, 0,010 mmol, 0.05 eq.) in 1,4-dioxane (1 mL). Purification by chromatography on silica gel (8/2 pentane/ Et_2O) afforded compound **4i** (28 %, 17 mg, 0.045 mmol) as a colorless oil.

^{1}H NMR (400 MHz, $CDCl_3$) δ 7.25 (dd, *J* = 7.6, 1.7 Hz, 1H), 7.16 – 7.11 (m, 2H), 7.09 – 7.01 (m, 3H), 6.88 (td, *J* = 7.5, 1.2 Hz, 1H), 6.59 (dd, *J* = 8.1, 1.2 Hz, 1H), 5.38 (d, *J* = 5.8 Hz, 1H), 4.86 (dd, *J* = 11.9, 1.4 Hz, 1H), 4.51 (dd, *J* = 11.9, 1.1 Hz, 1H), 4.31 (td, *J* = 8.4, 4.3 Hz, 1H), 4.08 (q, *J* = 8.0 Hz, 1H), 3.84 – 3.73 (m, 2H), 3.37 (tt, *J* = 7.3, 1.3 Hz, 1H), 2.78 (td, *J* = 9.4, 5.8 Hz, 1H), 2.50 (dtd, *J* = 12.7, 7.5, 4.3 Hz, 1H), 2.28 (dtd, *J* = 12.8, 8.3, 7.4 Hz, 1H), 2.05 (dddd, *J* = 12.9, 9.3, 6.7, 5.4 Hz, 1H), 1.79 – 1.60 (m, 1H). **^{13}C NMR** (101 MHz, $CDCl_3$) δ 209.1, 152.9, 135.4, 132.4, 129.1, 128.4, 127.7, 127.4, 125.3, 121.0, 116.2, 104.9, 101.6, 84.9, 78.7, 67.9, 66.8, 49.5, 49.1, 33.1, 27.0. **HRMS** ESI: Calculated for $C_{23}H_{22}ClO_3$ $[M+H]^+$ 381.1257, found 381.1255 (Diff.: -0.64 ppm).

3.10 Synthesis of **4j**

(2*S*,3*R*,3a'*R*,9a'*S*)-3-(1-(4-(trifluoromethyl)phenyl)propa-1,2-dien-1-yl)-2',3',3a',4,5,9a'-hexahydro-3*H*-spiro[furan-2,4'-furo[2,3-*b*]chromene]

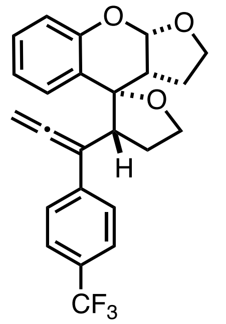

Compound **4j** was prepared following the general procedure using compound **1j** (50 mg, 0.145 mmol, 1 eq.), dihydrofuran (102 mg, 1.45 mmol, 10 eq.) and catalyst **A** (6 mg, 0,007 mmol, 0.05 eq.) in 1,4-dioxane (1 mL). Purification by chromatography on silica gel (8/2 pentane/ Et_2O) afforded compound **4j** (44 %, 26 mg, 0.064 mmol) as an amorphous yellow solid.

^{1}H NMR (400 MHz, $CDCl_3$) δ 7.42 – 7.36 (m, 2H), 7.30 – 7.23 (m, 1H), 7.19 (d, *J* = 8.1 Hz, 2H), 7.03 (ddd, *J* = 8.0, 7.3, 1.7 Hz, 1H), 6.87 (td, *J* = 7.5, 1.2 Hz, 1H), 6.53 (dd, *J* = 8.1, 1.2 Hz, 1H), 5.43 (d, *J* = 6.0 Hz, 1H), 5.02 – 4.82 (m, 1H), 4.62 (d, *J* = 12.1 Hz, 1H), 4.31 (td, *J* = 8.4, 4.7 Hz, 1H), 4.10 (q, *J* = 8.0 Hz, 1H), 3.91 – 3.68 (m, 2H), 3.57 – 3.35 (m, 1H), 2.82 (td, *J* = 9.4, 5.9 Hz, 1H), 2.54 (dtd, *J* = 12.6, 7.7, 4.7 Hz, 1H), 2.44 – 2.23 (m, 1H), 2.13 – 1.97 (m, 1H), 1.69 (ddt, *J* = 12.9, 9.5, 8.4 Hz, 1H). **^{13}C NMR** (101 MHz, $CDCl_3$) δ 209.6, 152.9, 140.7, 129.2, 128.7 (q, *J* = 32.4 Hz), 127.3, 126.6, 125.4, 125.1 (q, *J* = 3.8 Hz), 124.4 (q, *J* = 273.7 Hz), 121.1, 116.3, 105.3, 101.7, 85.0, 79.0, 67.9, 66.7, 49.4, 48.7, 33.0, 27.0. ^{19}F NMR (376 MHz, $CDCl_3$) δ – 62.48 (s). **HRMS** ESI: Calculated for $C_{24}H_{21}F_3NaO_3$ $[M+Na]^+$ 437.1340, found 437.1336 (Diff.: -0.31 ppm).

3.11 Synthesis of **4k**

(2*S*,3*R*,3a'*R*,9a'*S*)-3-(1-(4-nitrophenyl)propa-1,2-dien-1-yl)-2',3',3a',4,5,9a'-hexahydro-3*H*-spiro[furan-2,4'-furo[2,3-*b*]chromene]

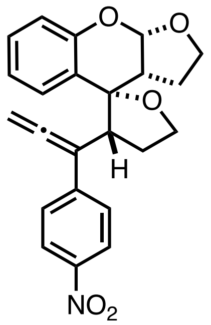

Compound **4k** was prepared following the general procedure using compound **1k** (50 mg, 0.205 mmol, 1 eq.), dihydrofuran (167 mg, 2.05 mmol, 10 eq.) and catalyst **A** (9 mg, 0,012 mmol, 0.05 eq.) in 1,4-dioxane (1 mL). Purification by chromatography on silica gel (8/2 pentane/ Et_2O) afforded compound **4k** (60 %, 48 mg, 0.12 mmol) as an orange oil.

^{1}H NMR (400 MHz, $CDCl_3$) δ 7.98 (d, *J* = 9.0 Hz, 2H), 7.26 (dd, *J* = 7.5, 1.7 Hz, 1H), 7.20 (d, *J* = 8.9 Hz, 2H), 7.06 – 6.97 (m, 1H), 6.86 (td, *J* = 7.5, 1.2 Hz, 1H), 6.49 (dd, *J* = 8.0, 1.2 Hz, 1H), 5.52 (d, *J* = 6.1 Hz, 1H), 5.04 (dd, *J* = 12.7, 1.6 Hz, 1H), 4.79 (dd, *J* = 12.7, 1.2 Hz, 1H), 4.31 (td, *J* = 8.5, 5.3 Hz, 1H), 4.09 (td, *J* = 8.4, 6.9 Hz, 1H), 3.77 (ddd, *J* = 8.5, 6.2, 3.4 Hz, 2H), 3.63 – 3.45 (m, 1H), 2.88 (td, *J* = 9.3, 6.1 Hz, 1H), 2.59 (dtd, *J* = 13.1, 8.0, 5.3 Hz, 1H), 2.40 – 2.28 (m, 1H), 2.04 (dddd, *J* = 12.6, 9.4, 7.2, 5.2 Hz, 1H), 1.69 (dq, *J* = 13.1, 8.6 Hz, 1H). **^{13}C NMR** (101 MHz, $CDCl_3$) δ 210.0, 152.7, 146.2, 143.7, 129.2, 127.2, 126.9, 125.6, 123.3, 121.3, 116.3, 105.8, 101.8, 84.8, 79.6, 67.9, 66.4, 49.1, 47.9, 32.7, 26.9. **HRMS** ESI: Calculated for $C_{23}H_{21}NNaO_5$ $[M+Na]^+$ 414.1317, found 414.1309 (Diff.: 0.35 ppm).

3.12 Synthesis of **4l**

(2*S*,3*R*,3a'*R*,9a'*S*)-3-(1-(3-nitrophenyl)propa-1,2-dien-1-yl)-2',3',3a',4,5,9a'-hexahydro-3*H*-spiro[furan-2,4'-furo[2,3-*b*]chromene]

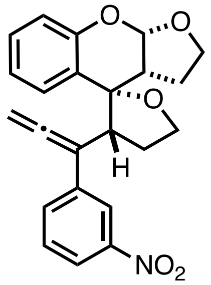

Compound **4l** was prepared following the general procedure using compound **1l** (51 mg, 0.159 mmol, 1 eq.), dihydrofuran (111 mg, 1.59 mmol, 10 eq.) and catalyst **A** (8 mg, 0,010 mmol, 0.05 eq.) in 1,4-dioxane (1 mL). Purification by chromatography on silica gel (8/2 pentane/ Et_2O) afforded compound **4l** (34 %, 21 mg, 0.054 mmol) as an orange oil.

^{1}H NMR (400 MHz, $CDCl_3$) δ 7.90 (ddd, *J* = 8.0, 2.3, 1.2 Hz, 1H), 7.81 (t, *J* = 2.0 Hz, 1H), 7.34 (dt, *J* = 7.8, 1.5 Hz, 1H), 7.31 – 7.23 (m, 2H), 7.04 – 6.92 (m, 1H), 6.87 (td, *J* = 7.4, 1.3 Hz, 1H), 6.42 (dd, *J* = 8.0, 1.3 Hz, 1H), 5.53 (d, *J* = 6.0 Hz, 1H), 5.03 (dd, *J* = 12.2, 1.7 Hz, 1H), 4.83 (dd, *J* = 12.3, 1.4 Hz, 1H), 4.30 (td, *J* = 8.4, 5.5 Hz, 1H), 4.10 (td, *J* = 8.4, 6.8 Hz, 1H), 3.84 – 3.70 (m, 2H), 3.51 (ddt, *J* = 7.2, 5.4, 1.6 Hz, 1H), 2.88 (td, *J* = 9.3, 6.0 Hz, 1H), 2.68 – 2.50 (m, 1H), 2.43 – 2.28 (m, 1H), 2.04 (dddd, *J* = 12.7, 9.4, 7.3, 5.0 Hz, 1H), 1.76 – 1.60 (m, 1H). **^{13}C NMR** (101 MHz, $CDCl_3$) δ 209.2, 152.8, 148.1, 138.7, 132.3, 129.1, 128.8, 127.1, 125.6, 125.5, 121.4, 121.3, 116.2, 105.5, 101.8, 84.9, 79.7, 68.0, 66.5, 49.1, 48.3, 32.6, 26.9. **HRMS** ESI: Calculated for $C_{23}H_{21}NNaO_5$ $[M+Na]^+$ 414.1317, found 414.1296 (Diff.: 4.40 ppm).

2.13 Synthesis of **4m**

(2*S*,3*R*,3a'*R*,9a'*S*)-3-(1-(thiophen-2-yl)propa-1,2-dien-1-yl)-2',3',3a',4,5,9a'-hexahydro-3*H*-spiro[furan-2,4'-furo[2,3-*b*]chromene]

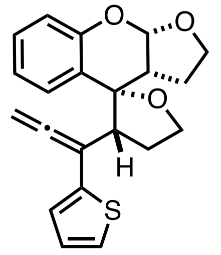

Compound **4m** was prepared following the general procedure using compound **1m** (50 mg, 0.177 mmol, 1 eq.), dihydrofuran (124 mg, 1.77 mmol, 10 eq.) and catalyst **A** (7 mg, 0,09 mmol, 0.05 eq.) in 1,4-dioxane (1 mL). Purification by chromatography on silica gel (8/2 pentane/ Et_2O) afforded compound **4m** (65 %, 41 mg, 0.117 mmol) as an amorphous yellow solid.

^{1}H NMR (400 MHz, $CDCl_3$) δ 7.24 (dd, *J* = 7.6, 1.7 Hz, 1H), 7.15 – 7.05 (m, 2H), 6.93 – 6.79 (m, 3H), 6.66 (dd, *J* = 8.1, 1.2 Hz, 1H), 5.54 (d, *J* = 5.7 Hz, 1H), 4.89 (d, *J* = 12.3 Hz, 1H), 4.48 (dd, *J* = 12.3, 0.9 Hz, 1H), 4.30 (td, *J* = 8.5, 3.9 Hz, 1H), 4.14 – 4.00 (m, 1H), 3.92 – 3.75 (m, 2H), 3.42 – 3.25 (m, 1H), 2.83 (td, *J* = 9.5, 5.7 Hz, 1H), 2.47 (dtd, *J* = 12.8, 7.4, 3.9 Hz, 1H), 2.25 (dq, *J* = 12.8, 8.4 Hz, 1H), 2.08 (dddd, *J* = 12.8, 9.2, 7.2, 4.9 Hz, 1H), 1.72 (ddt, *J* = 12.9, 9.8, 8.5 Hz, 1H).**^{13}C NMR** (101 MHz, $CDCl_3$) δ 208.4, 153.0, 141.3, 129.1, 127.5, 127.3, 124.9, 124.5, 123.0, 120.8, 116.1, 101.7, 100.8, 84.9, 79.5, 67.8, 66.8, 51.1, 49.6, 32.9, 27.1. **HRMS** ESI: Calculated for $C_{21}H_{21}SO_3$ $[M+H]^+$ 353.1211, found 353.1196 (Diff.: 2.77 ppm).

2.14 Synthesis of **4n**

(2*S*,3*R*,3a'*R*,9a'*S*)-3-(1-(benzo[*b*]thiophen-2-yl)propa-1,2-dien-1-yl)-2',3',3a',4,5,9a'-hexahydro-3*H*-spiro[furan-2,4'-furo[2,3-*b*]chromene]

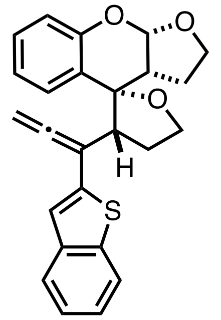

Compound **4i** was prepared following the general procedure using compound **1i** (50 mg, 0.150 mmol, 1 eq.), dihydrofuran (105 mg, 1.50 mmol, 10 eq.) and catalyst **A** (6 mg, 0,008 mmol, 0.05 eq.) in 1,4-dioxane (1 mL). Purification by chromatography on silica gel (8/2 pentane/ Et_2O) afforded compound **4i** (68 %, 41 mg, 0.102 mmol) as an orange oil.

^{1}H NMR (400 MHz, $CDCl_3$) δ 7.7 – 7.6 (m, 2H), 7.3 – 7.2 (m, 3H), 7.2 – 7.0 (m, 2H), 6.9 (td, *J* = 7.5, 1.2 Hz, 1H), 6.7 (dd, *J* = 8.0, 1.2 Hz, 1H), 5.6 (d, *J* = 5.8 Hz, 1H), 5.0 (dt, *J* = 12.8, 1.1 Hz, 1H), 4.5 (dt, *J* = 12.8, 1.0 Hz, 1H), 4.3 (td, *J* = 8.5, 3.6 Hz, 1H), 4.1 (td, *J* = 8.6, 7.5 Hz, 1H), 4.0 – 3.7 (m, 2H), 3.4 (dd, *J* = 8.4, 7.1 Hz, 1H), 2.9 (td, *J* = 9.4, 5.8 Hz, 1H), 2.5 (dtd, *J* = 12.8, 7.3, 3.6 Hz, 1H), 2.4 – 2.2 (m, 1H), 2.2 – 2.0 (m, 1H), 1.7 (ddt, *J* = 12.9, 9.7, 8.4 Hz, 1H). **^{13}C NMR** (101 MHz, $CDCl_3$) δ 209.3, 153.0, 141.9, 140.4, 139.4, 129.2, 127.5, 125.2, 124.3, 124.2, 123.3, 122.0,

121.0, 119.2, 116.3, 101.9, 101.4, 85.0, 80.0, 67.8, 66.8, 50.1, 49.7, 33.0, 27.2. **HRMS** ESI: Calculated for $C_{25}H_{23}SO_3$ $[M+H]^+$ 403.1368, found 403.1354 (Diff.: 1.64 ppm).

4. 1H and 13C NMR spectra

1f (400 MHz, $CDCl_3$)

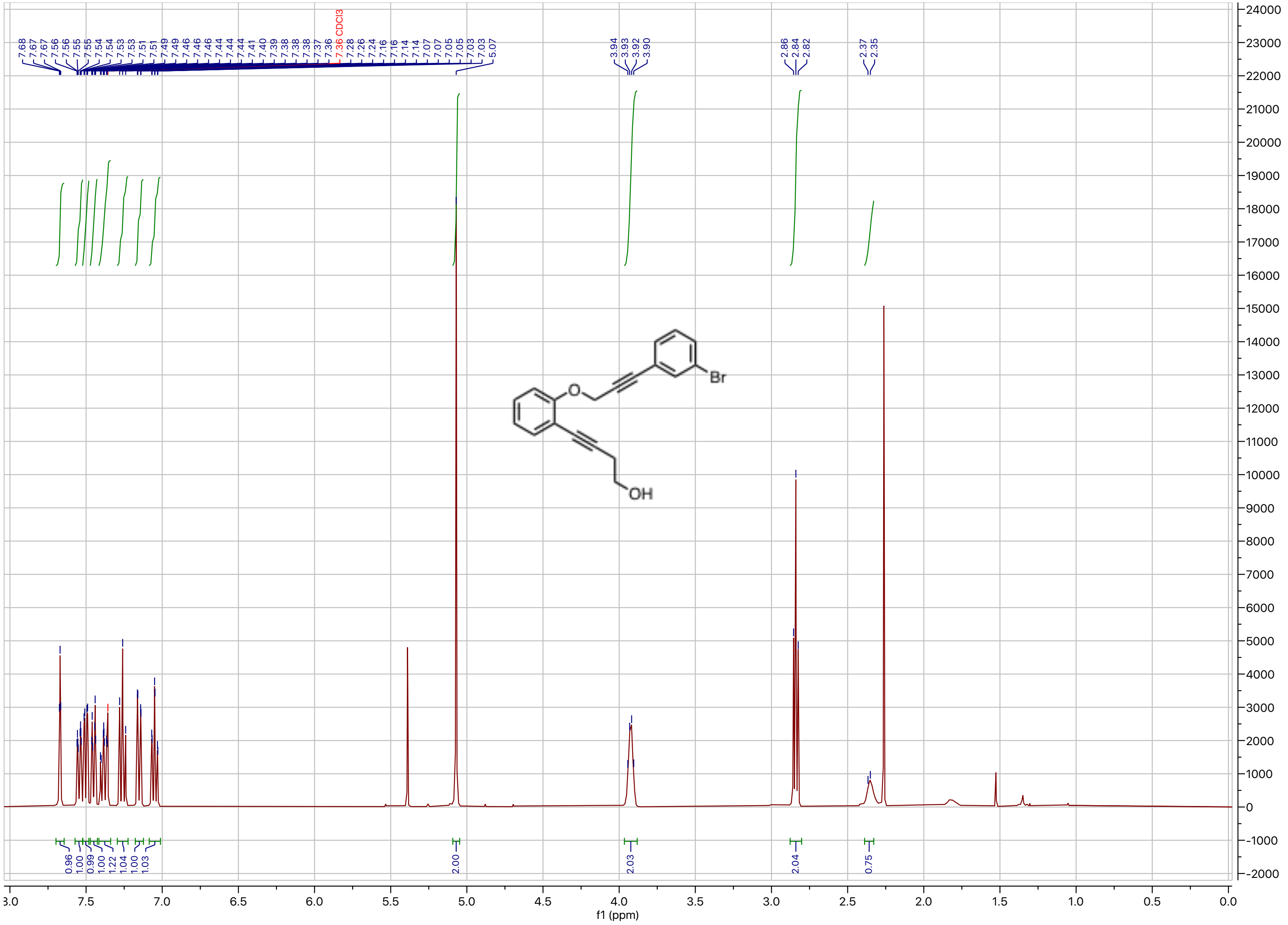

1f (101 MHz, $CDCl_3$)

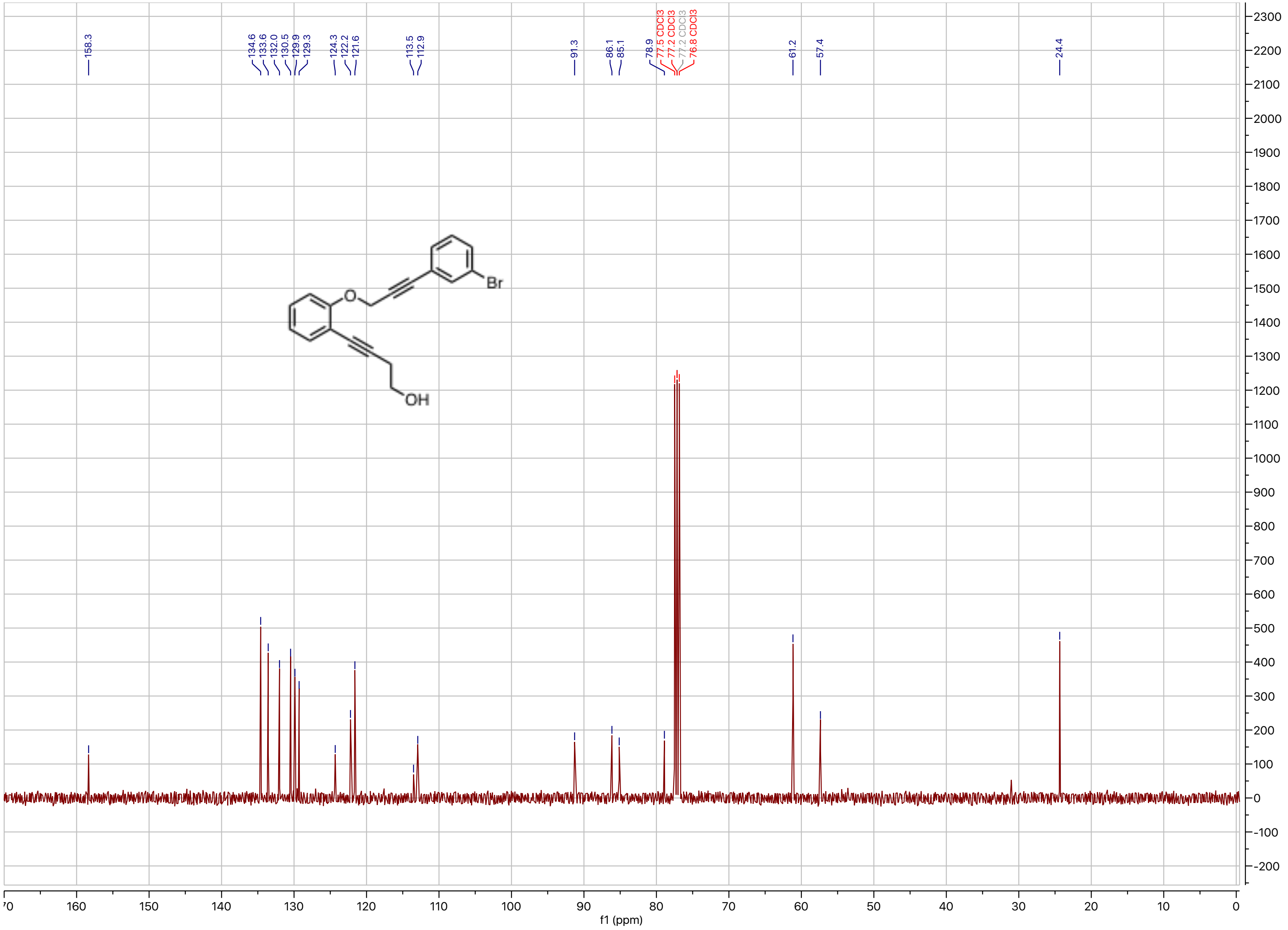

1g (400 MHz, $CDCl_3$)

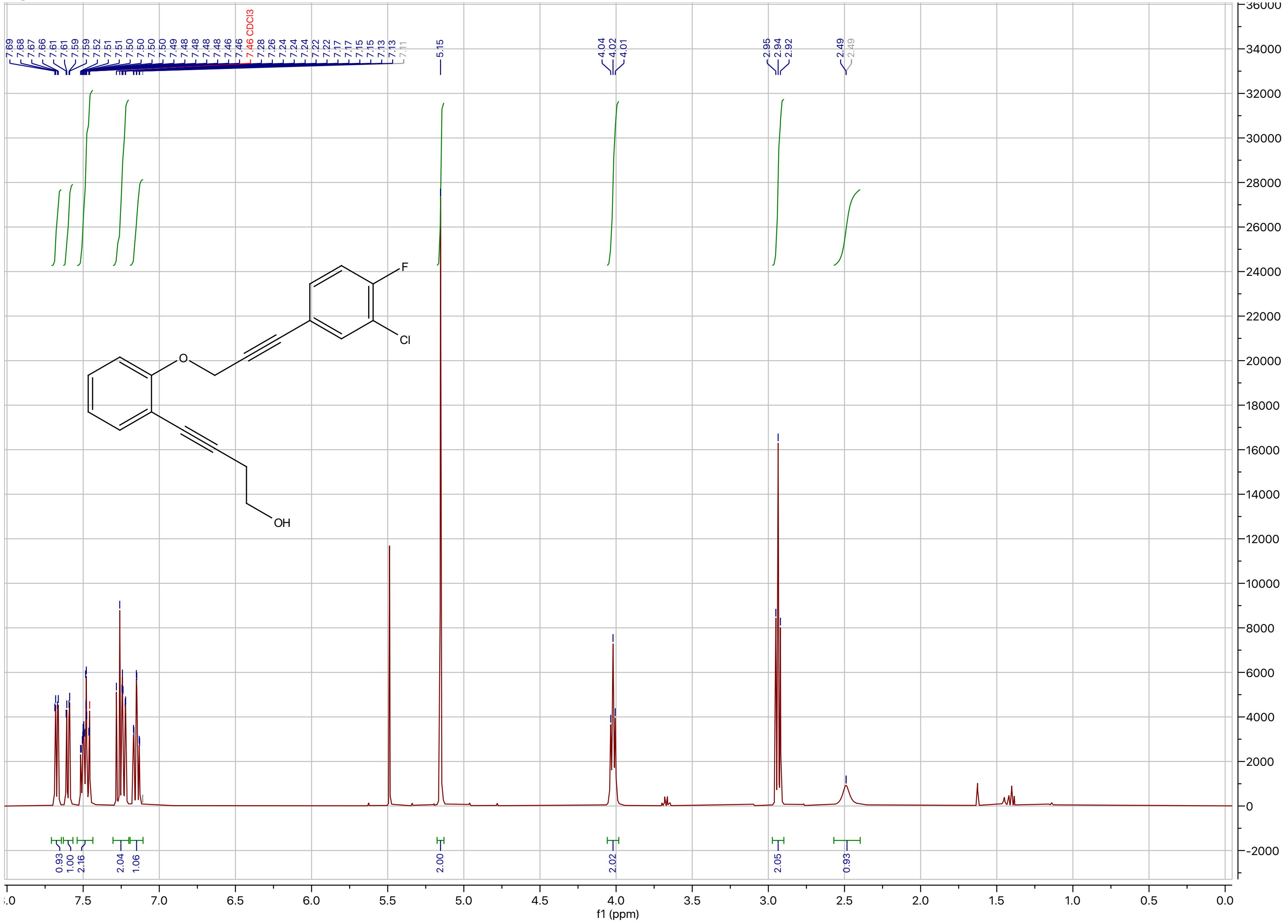

4a (400 MHz, $CDCl_3$)

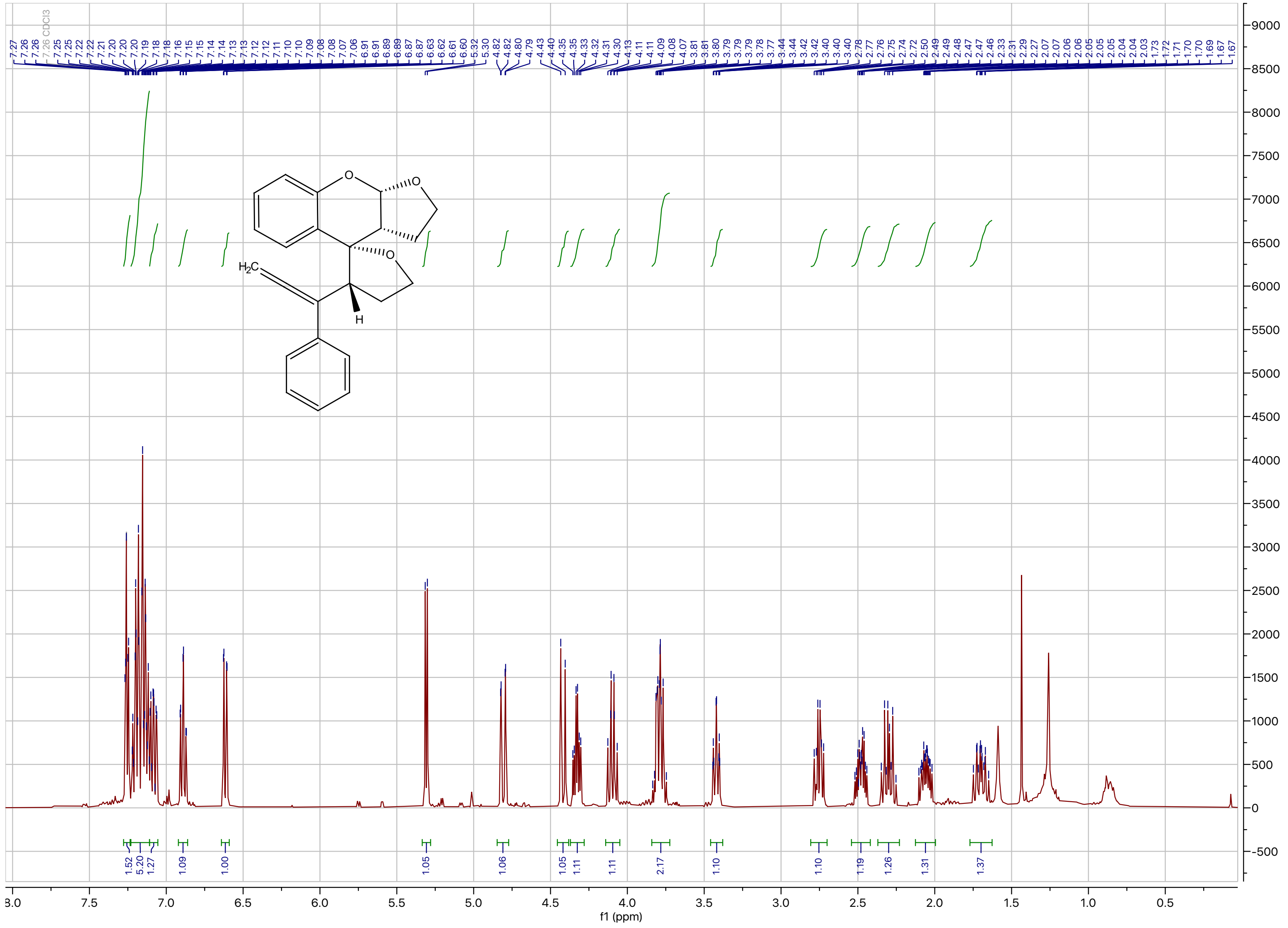

4a (101 MHz, $CDCl_3$)

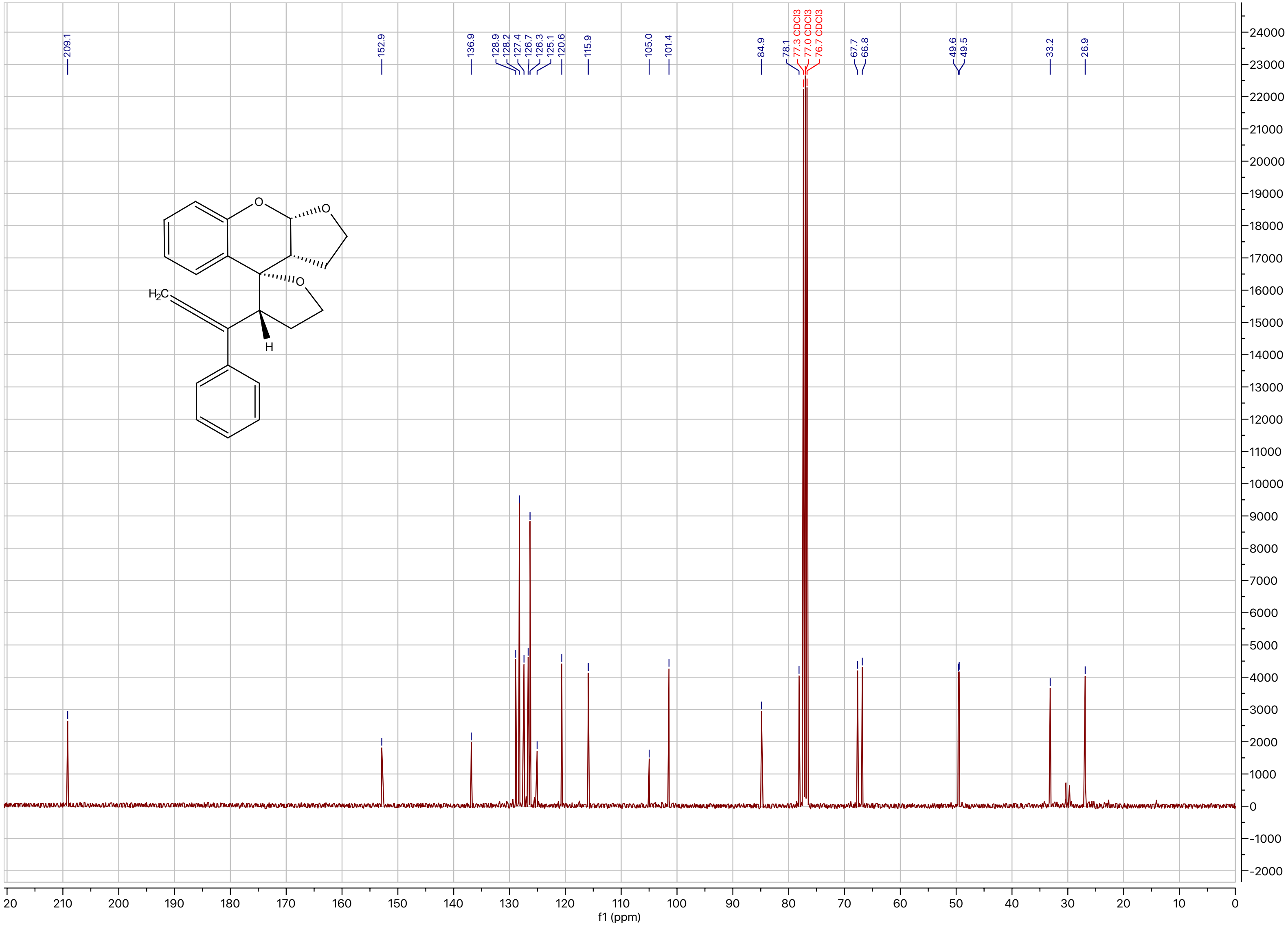

4b (400 MHz, CDCl3)

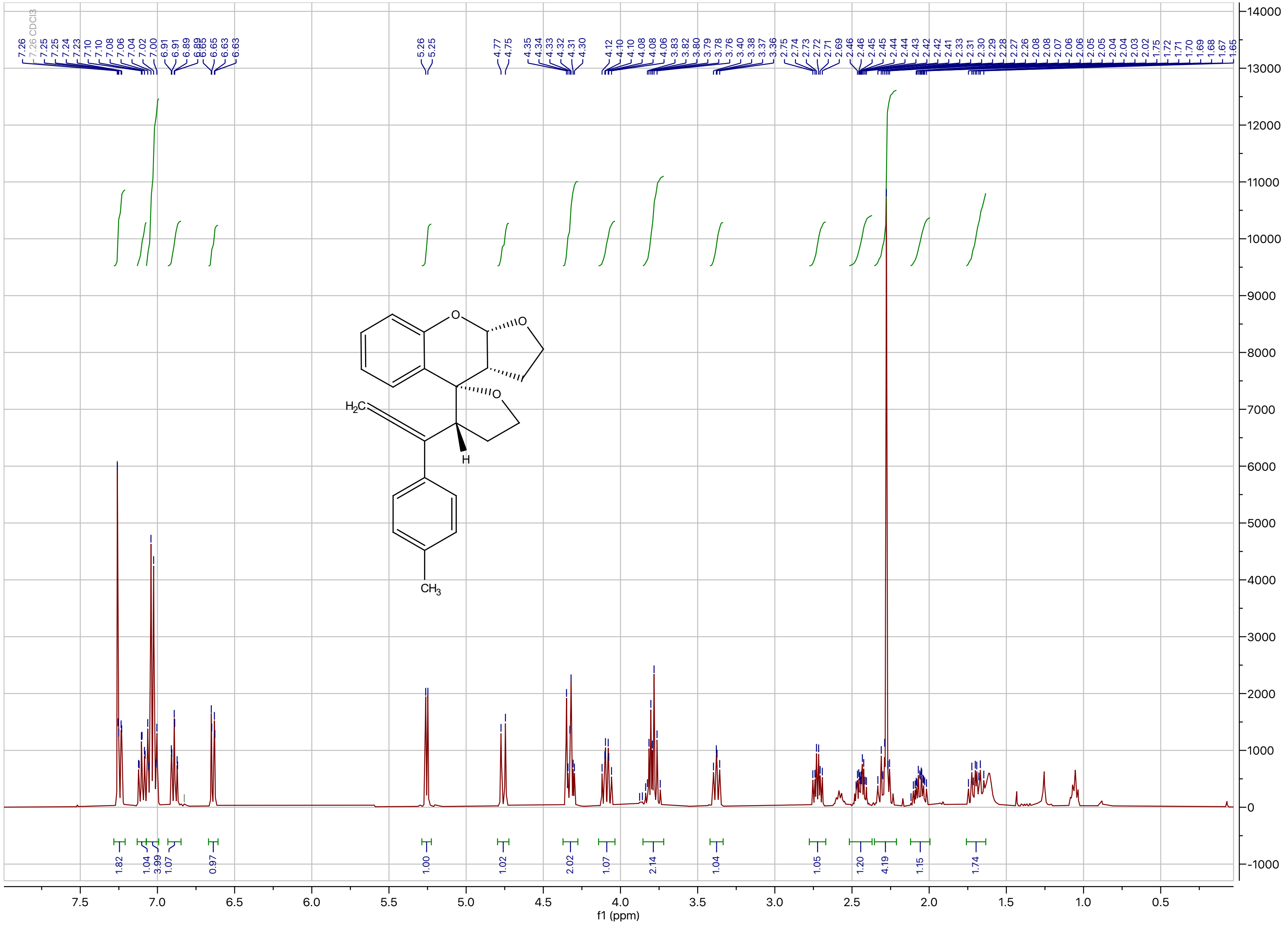

4b (101 MHz, $CDCl_3$)

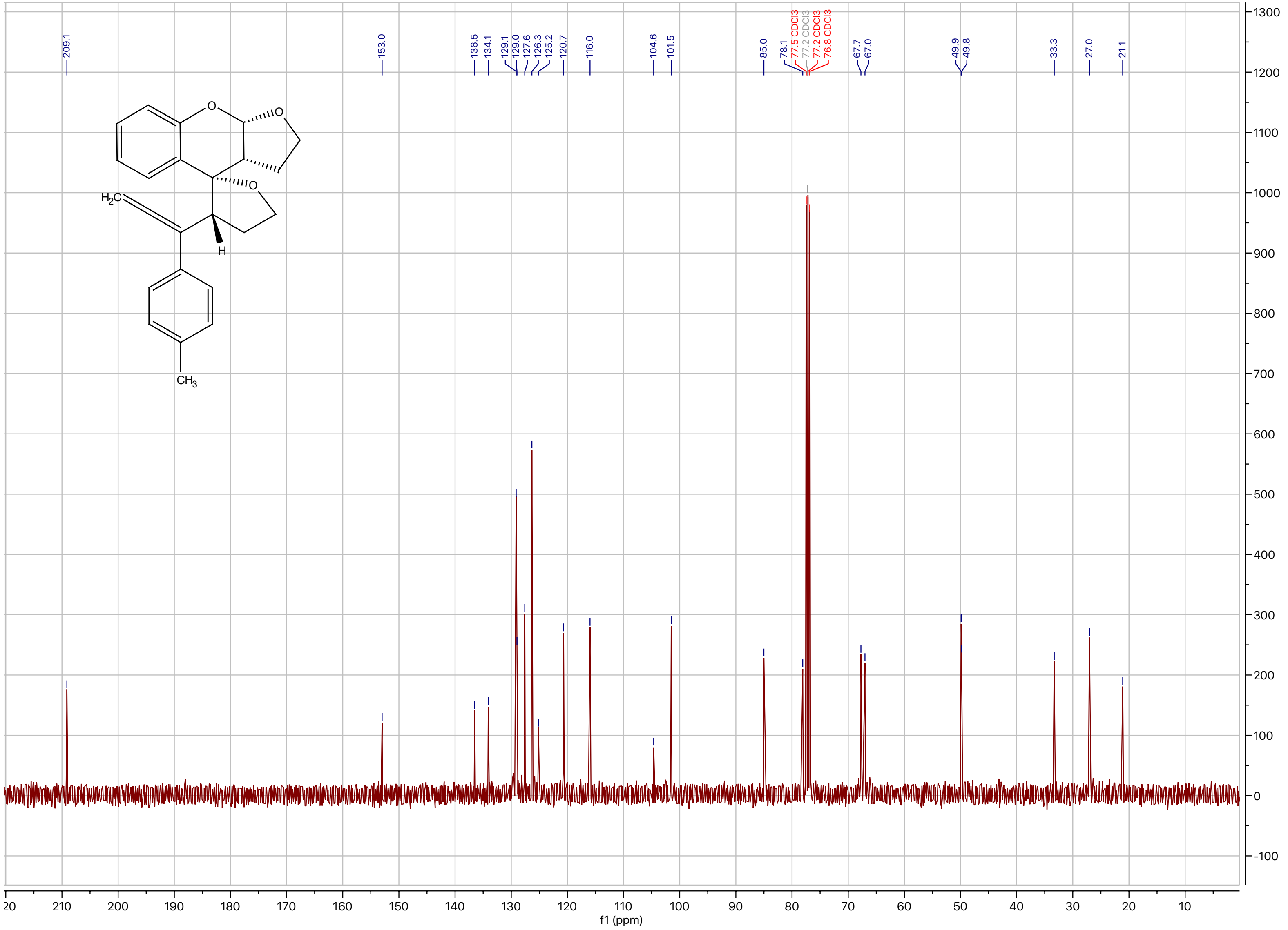

4c (400 MHz, $CDCl_3$)

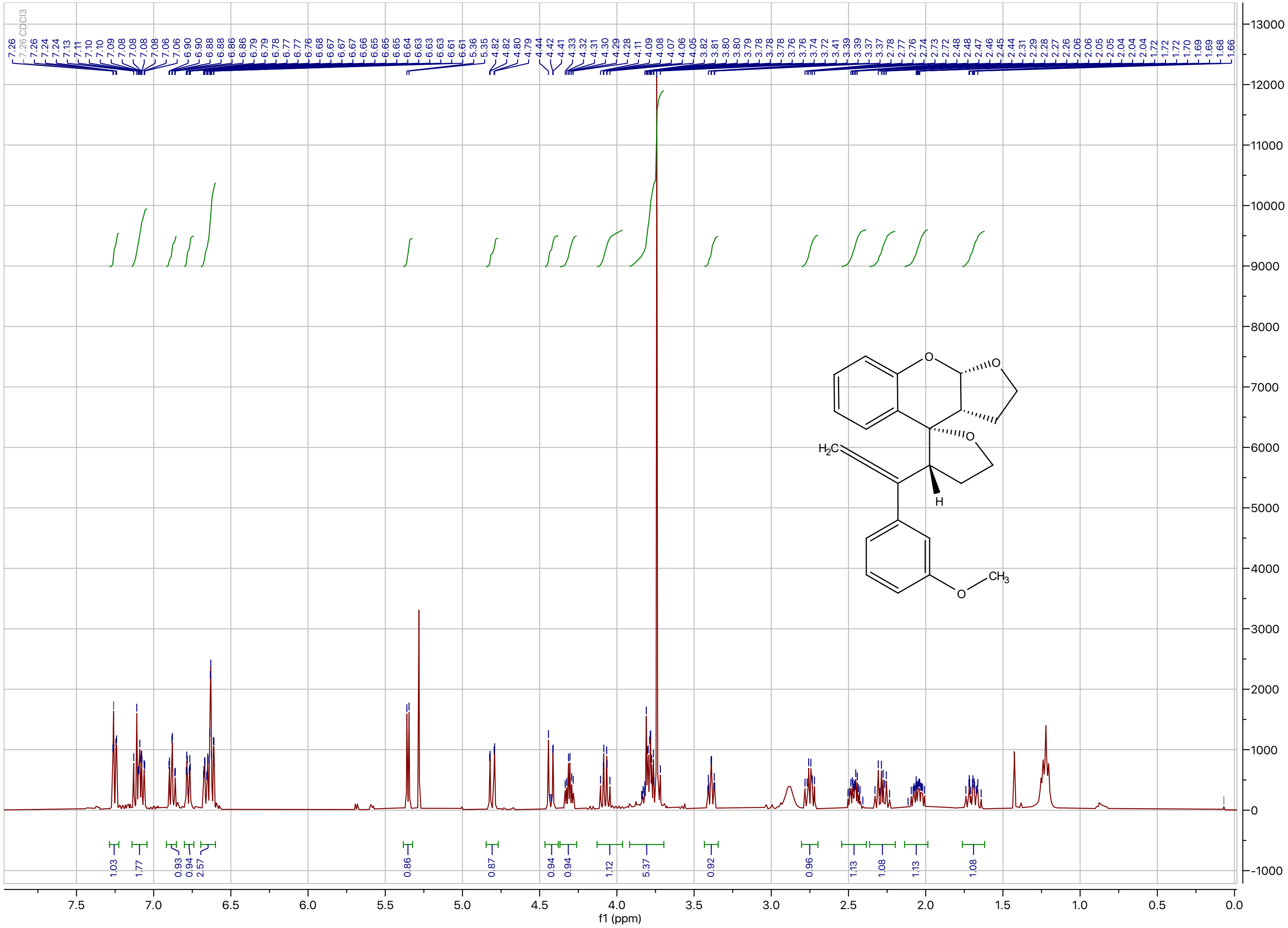

4c (101 MHz, $CDCl_3$)

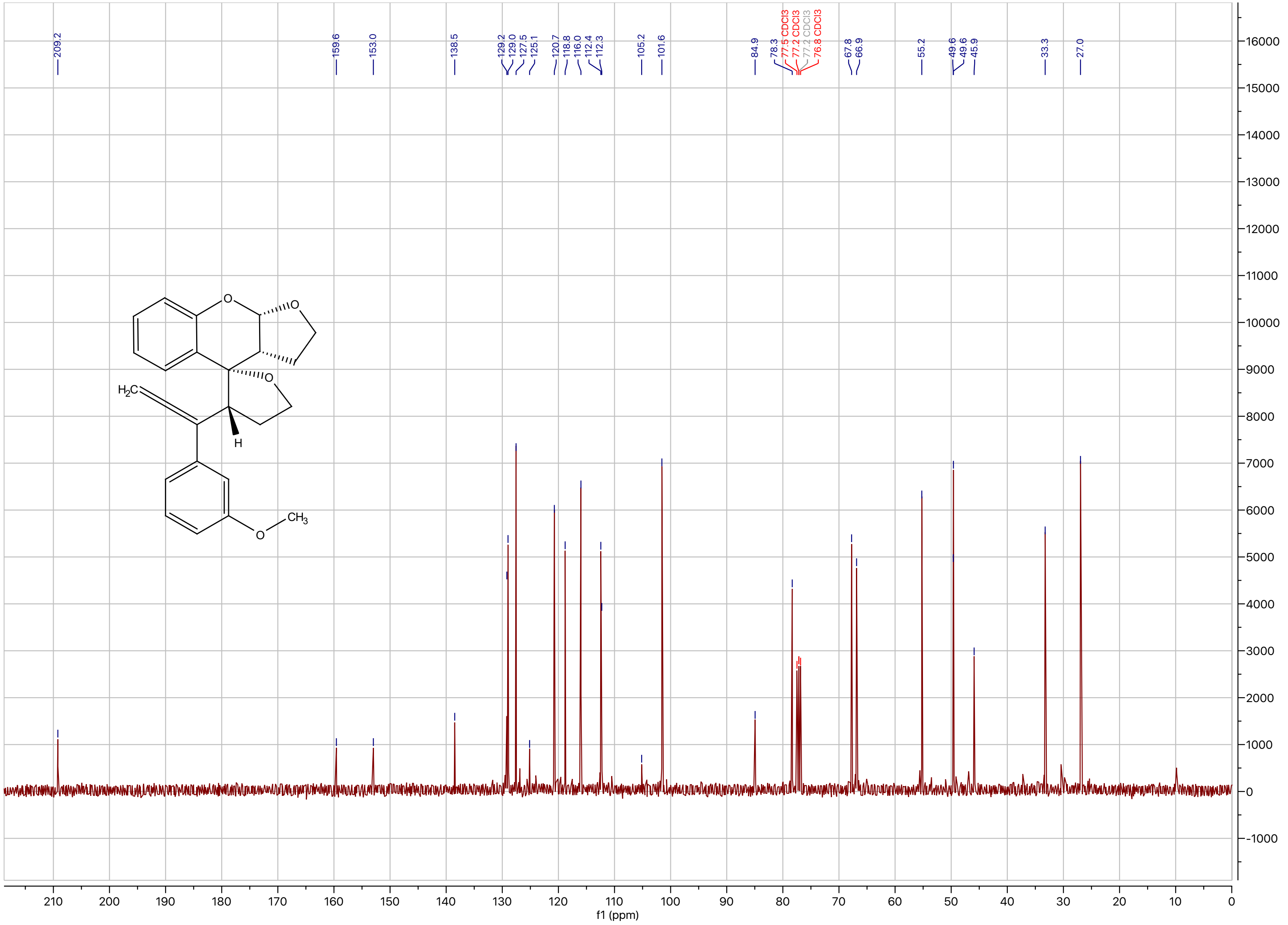

4d (400 MHz, $CDCl_3$)

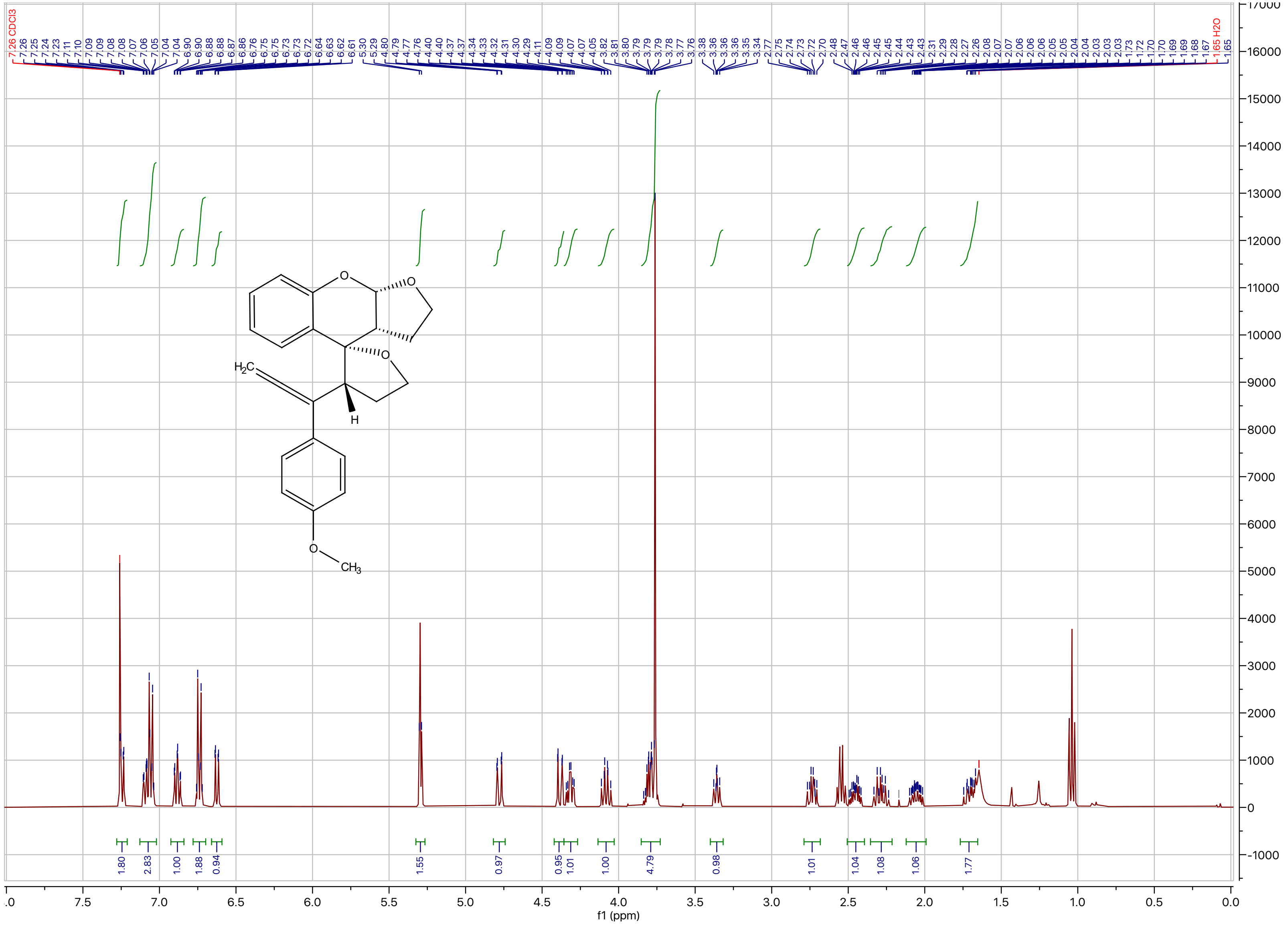

4d (101 MHz, $CDCl_3$)

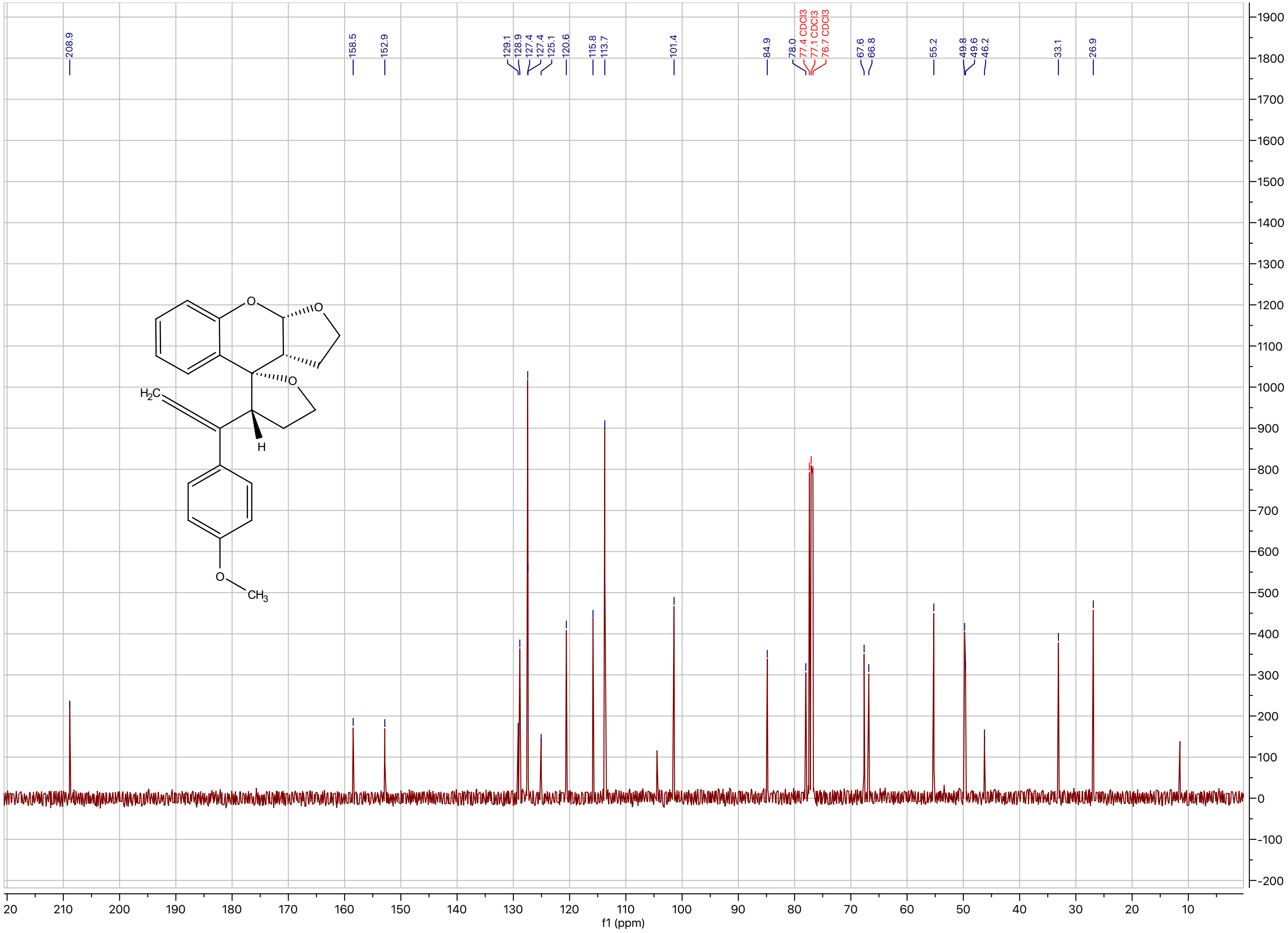

4e (400 MHz, $CDCl_3$)

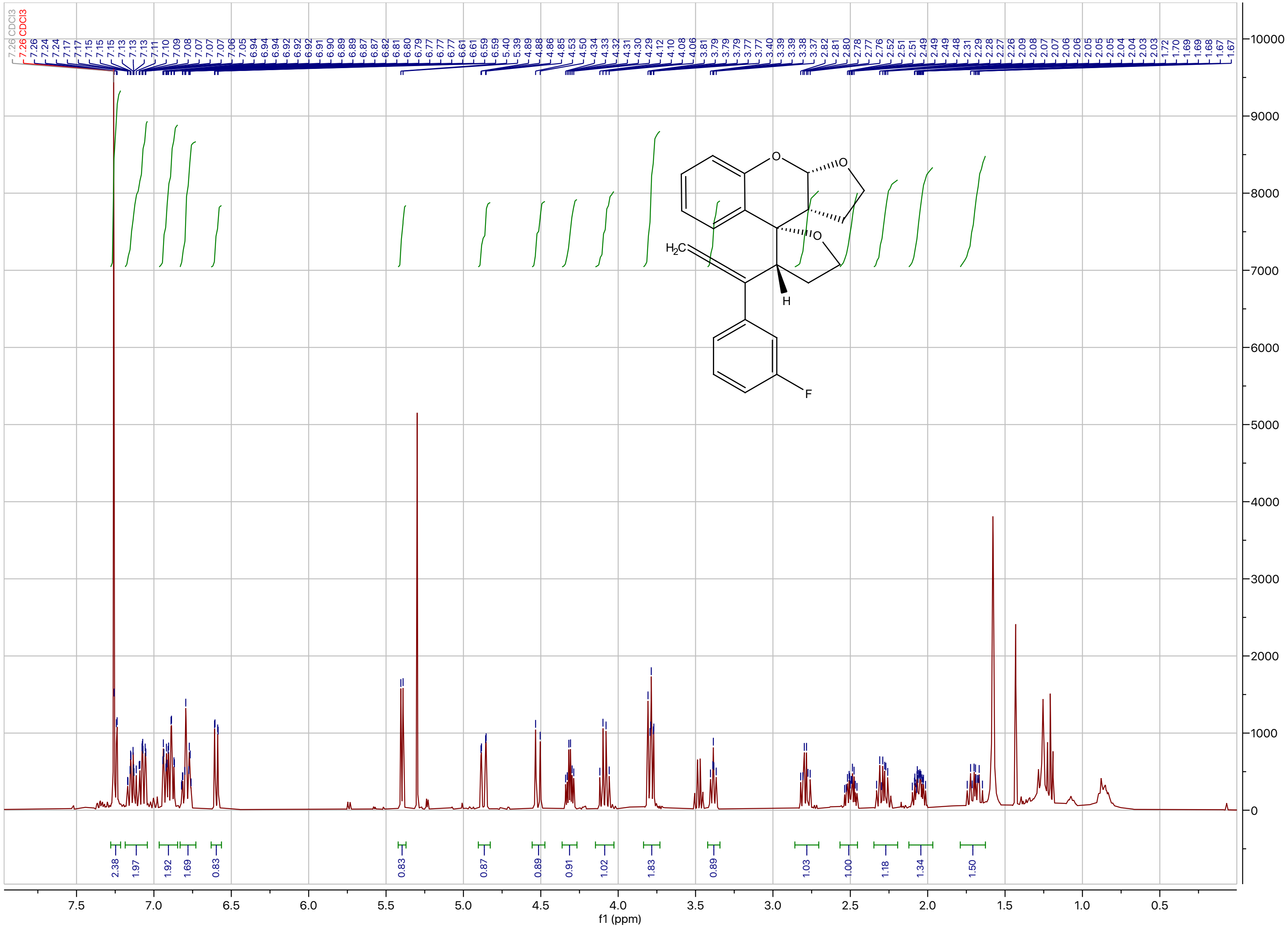

4e (101 MHz, $CDCl_3$)

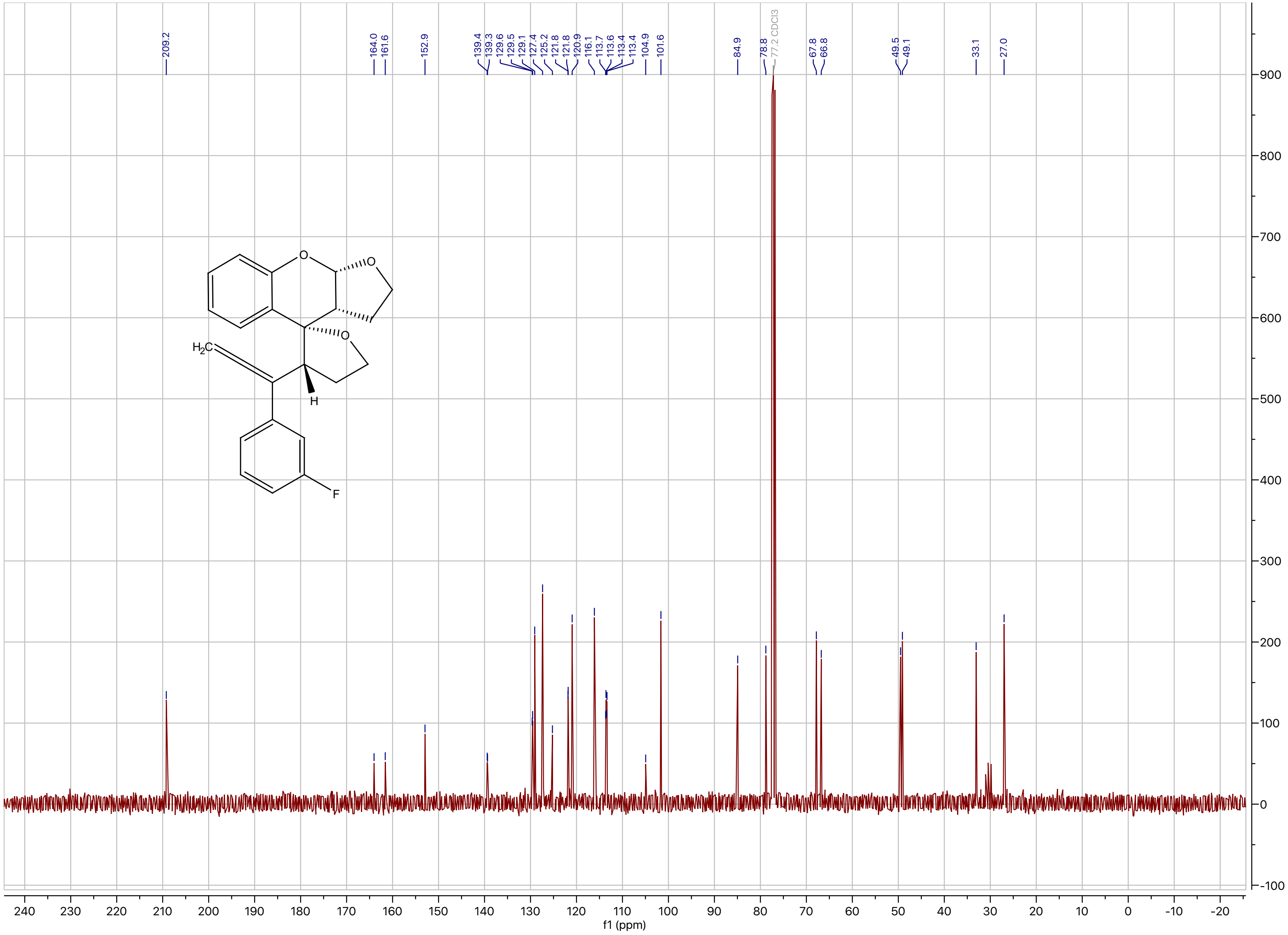

4f (400 MHz, $CDCl_3$)

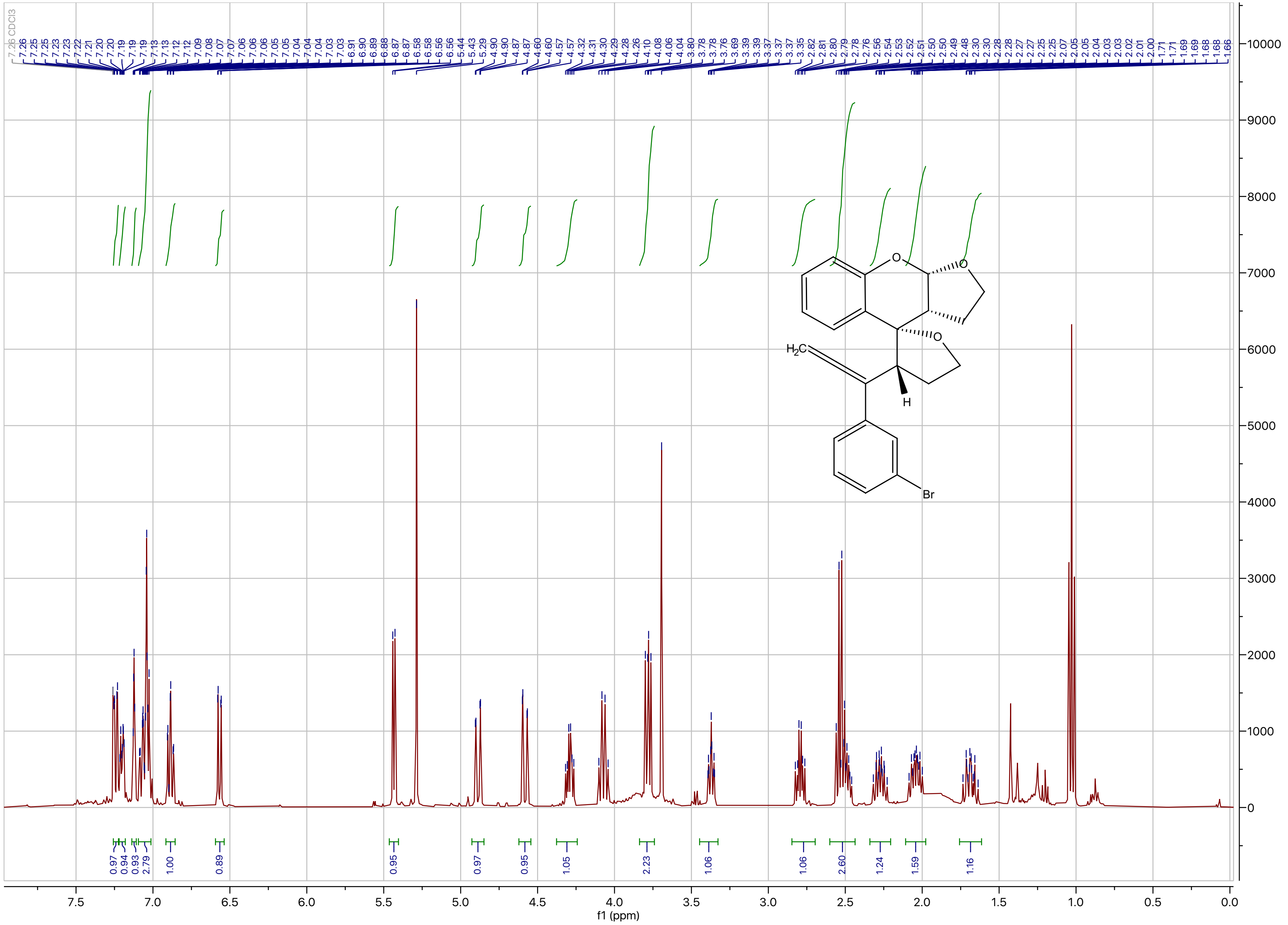

4f (101 MHz, $CDCl_3$)

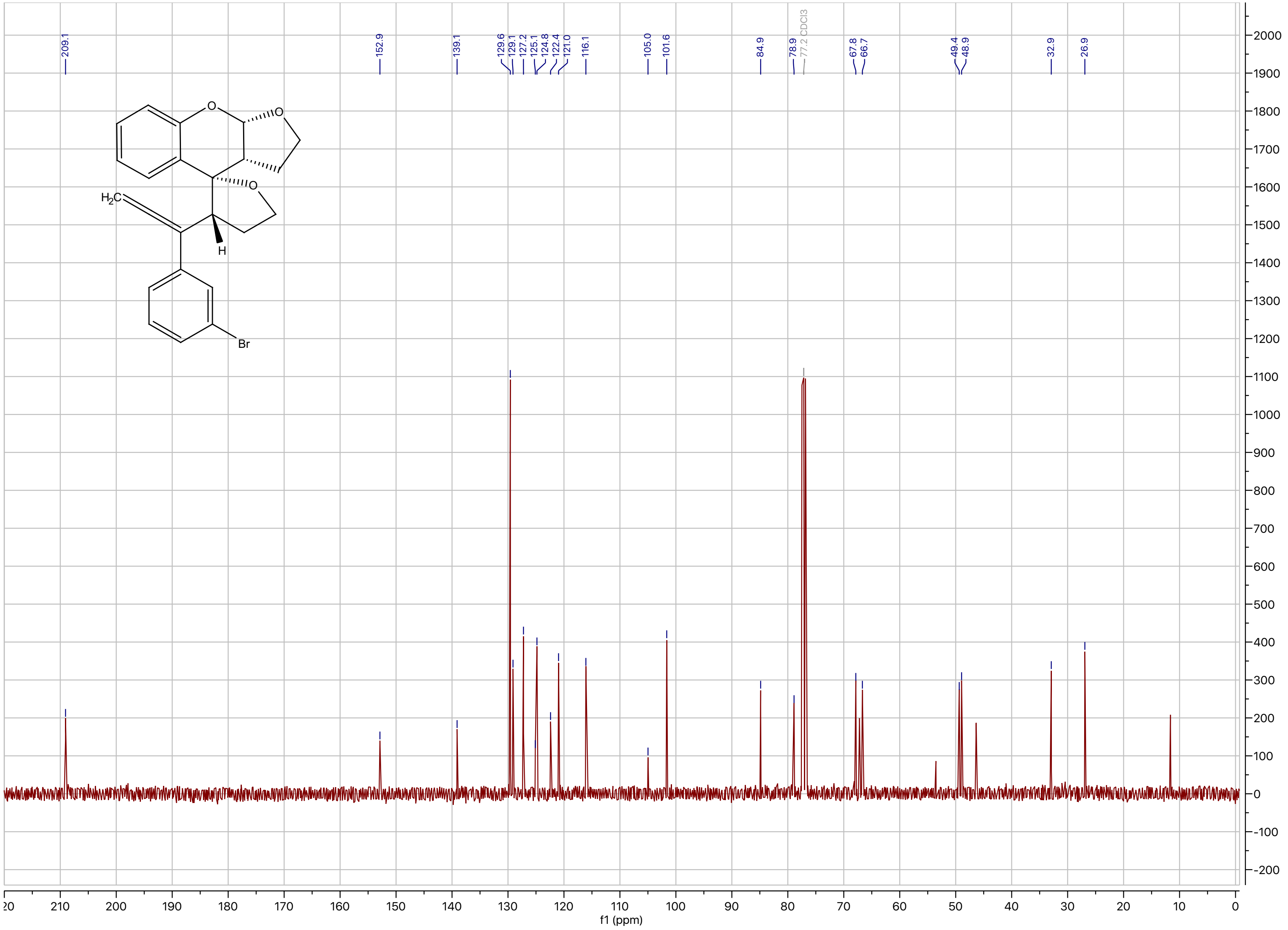

4g (400 MHz, $CDCl_3$)

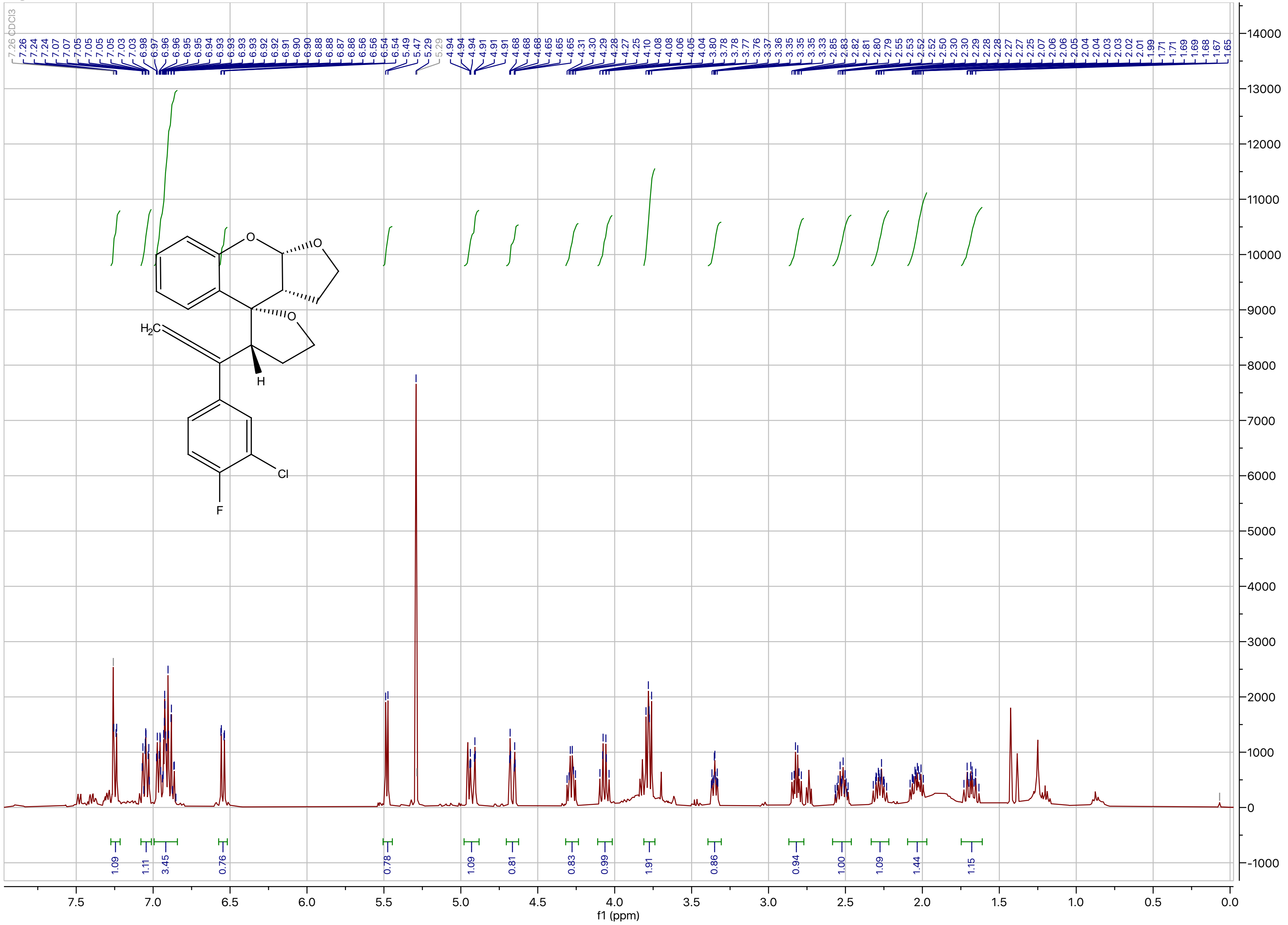

4g (101 MHz, $CDCl_3$)

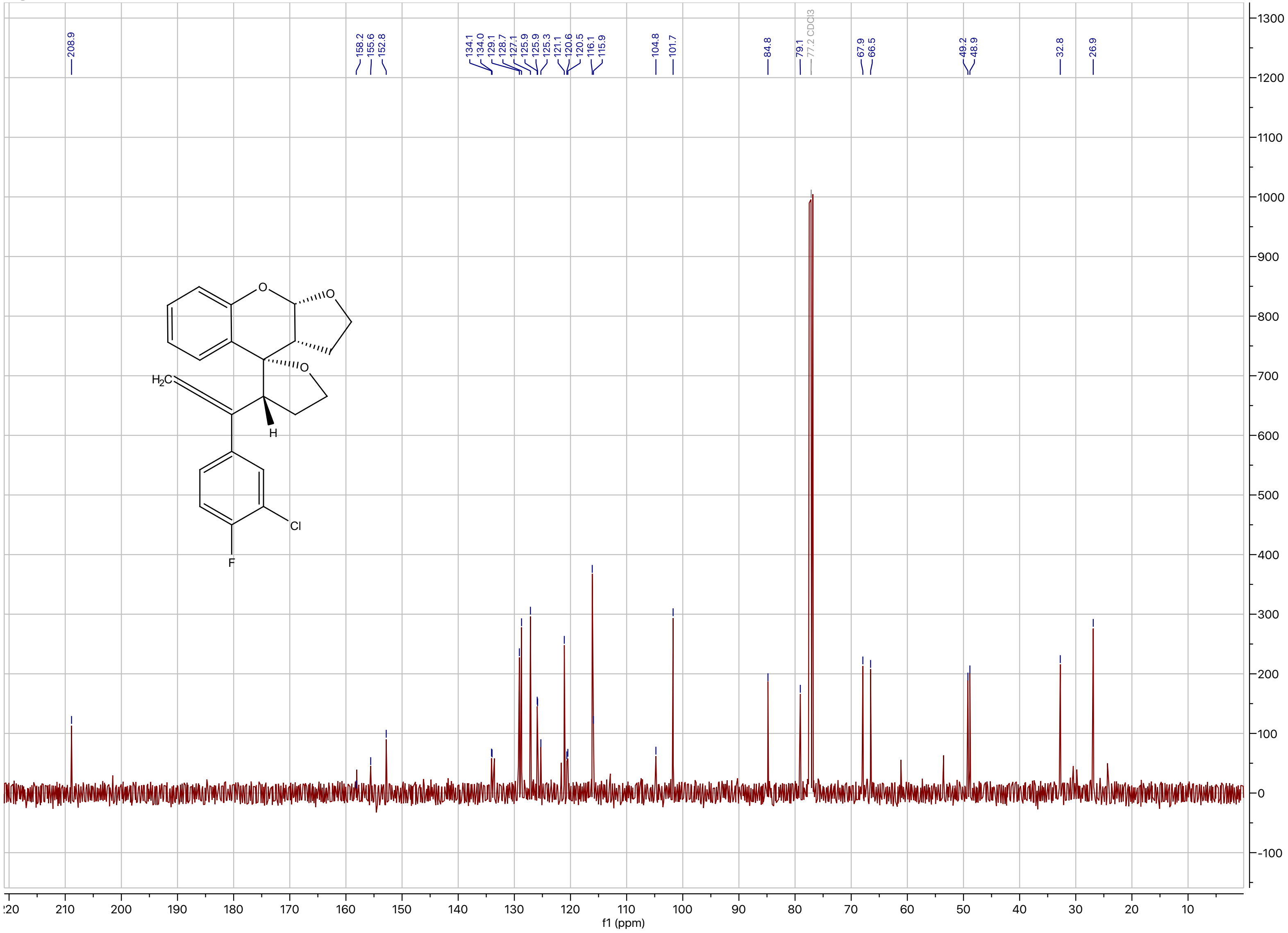

4h (400 MHz, $CDCl_3$)

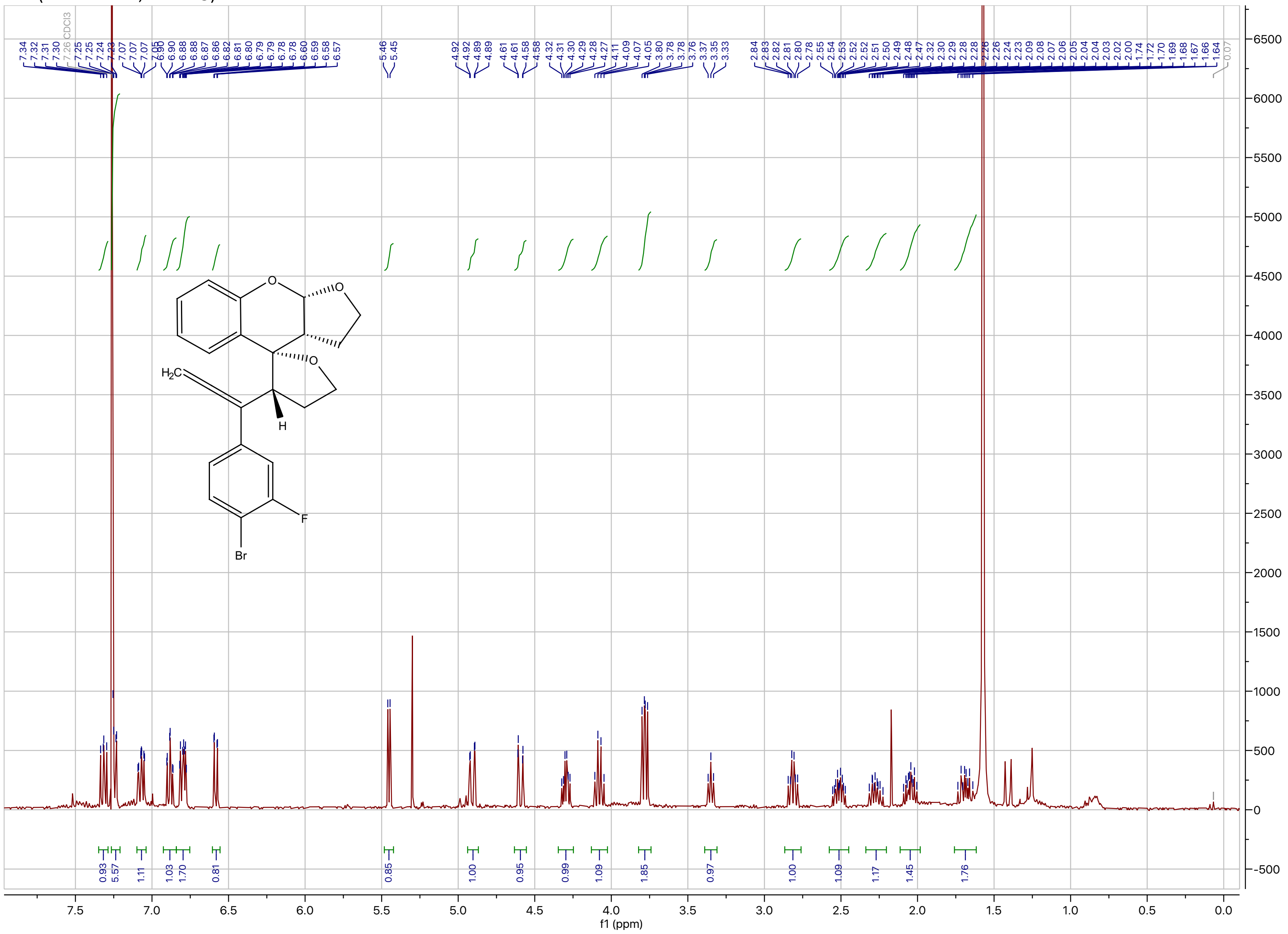

4h (101 MHz, $CDCl_3$)

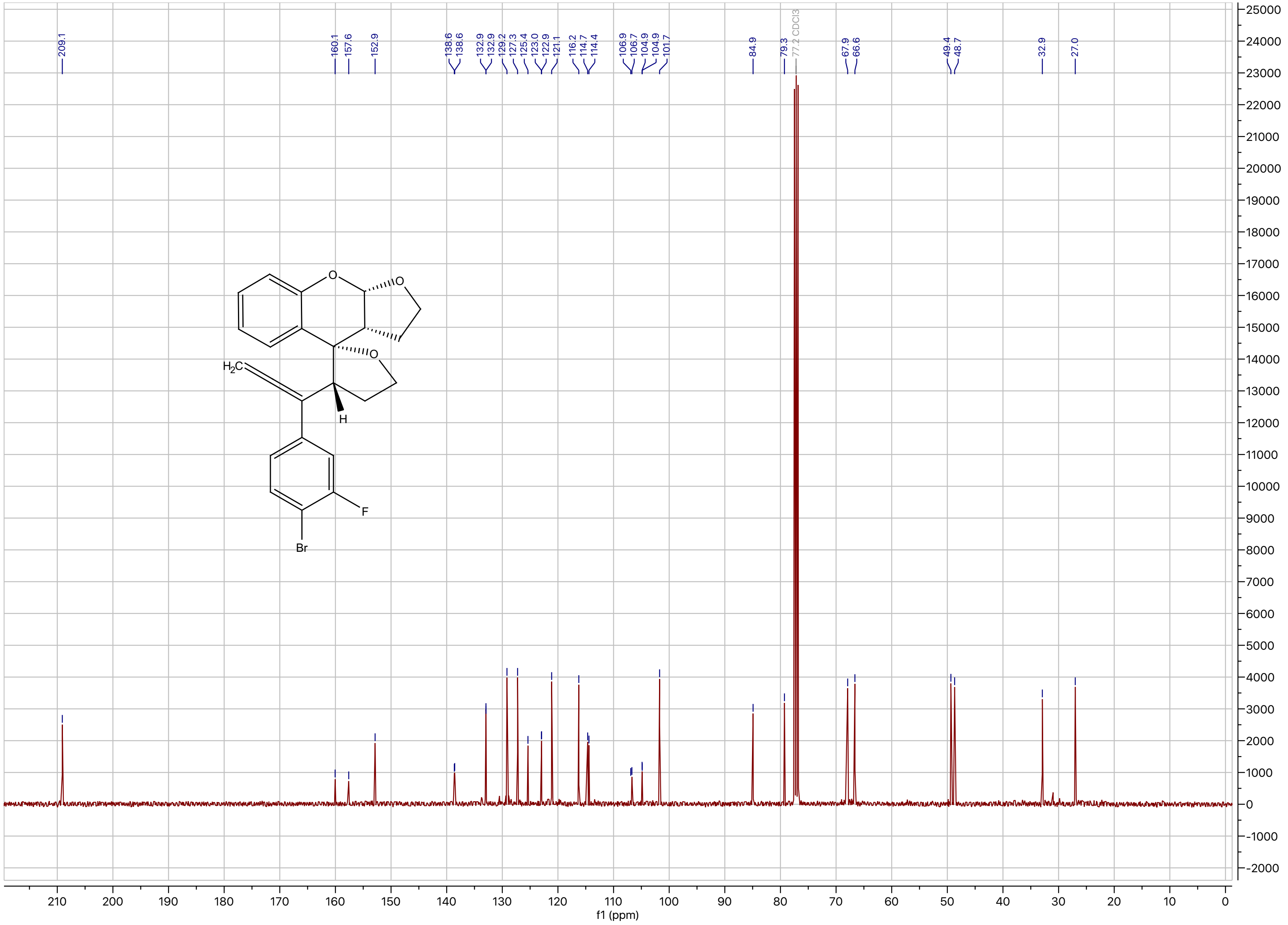

4i (400 MHz, $CDCl_3$)

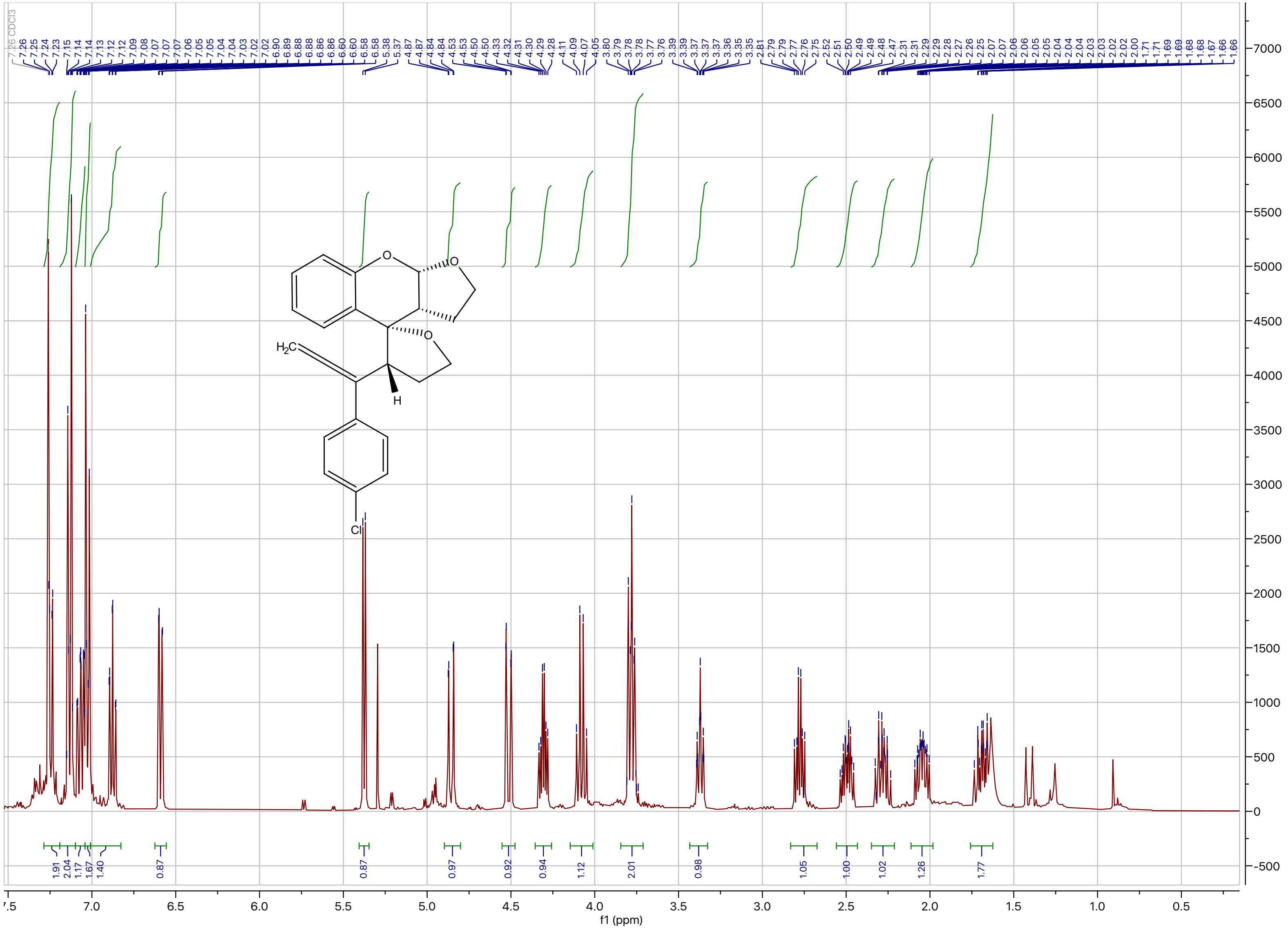

4i (101 MHz, $CDCl_3$)

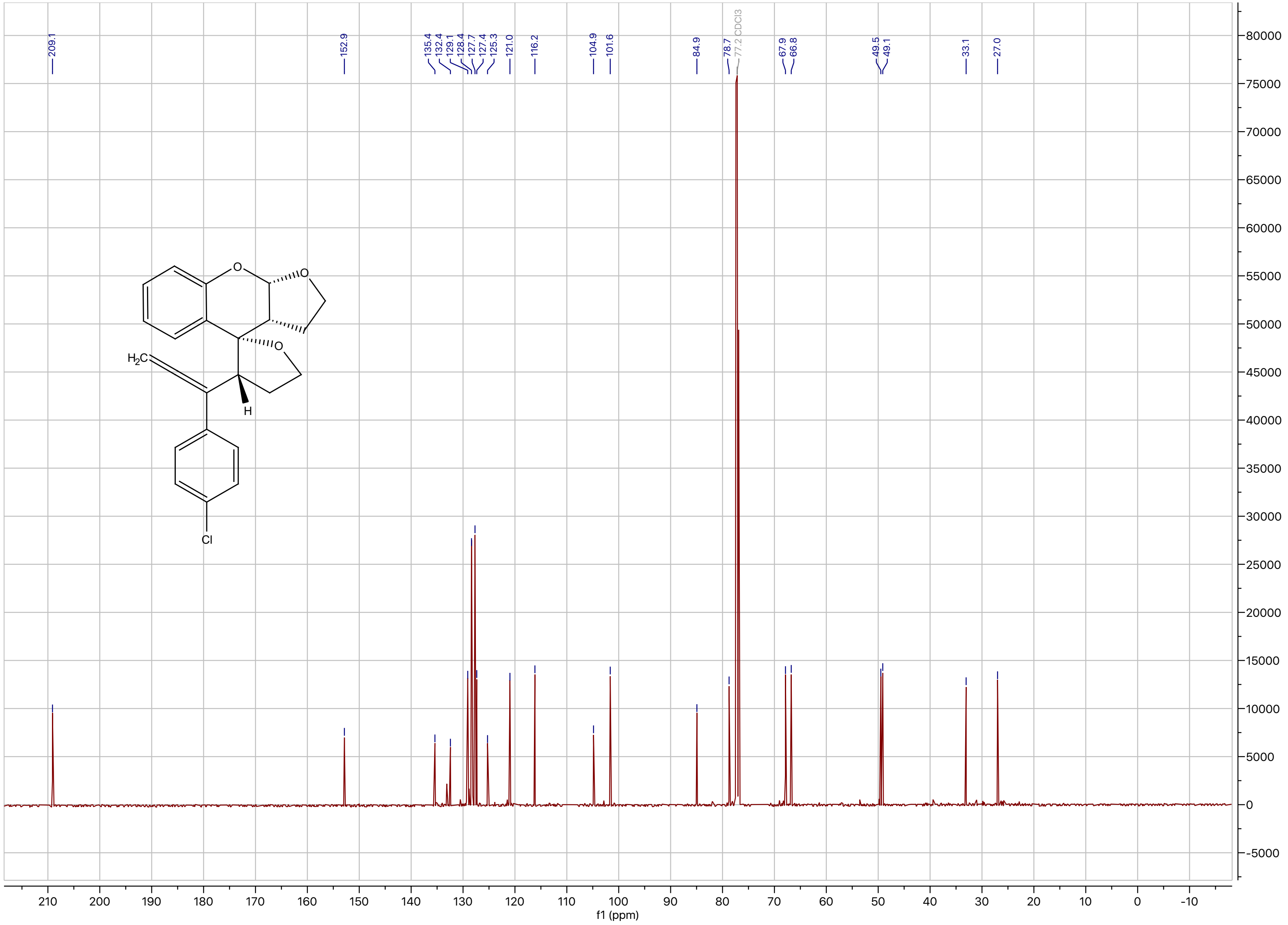

4j (400 MHz, $CDCl_3$)

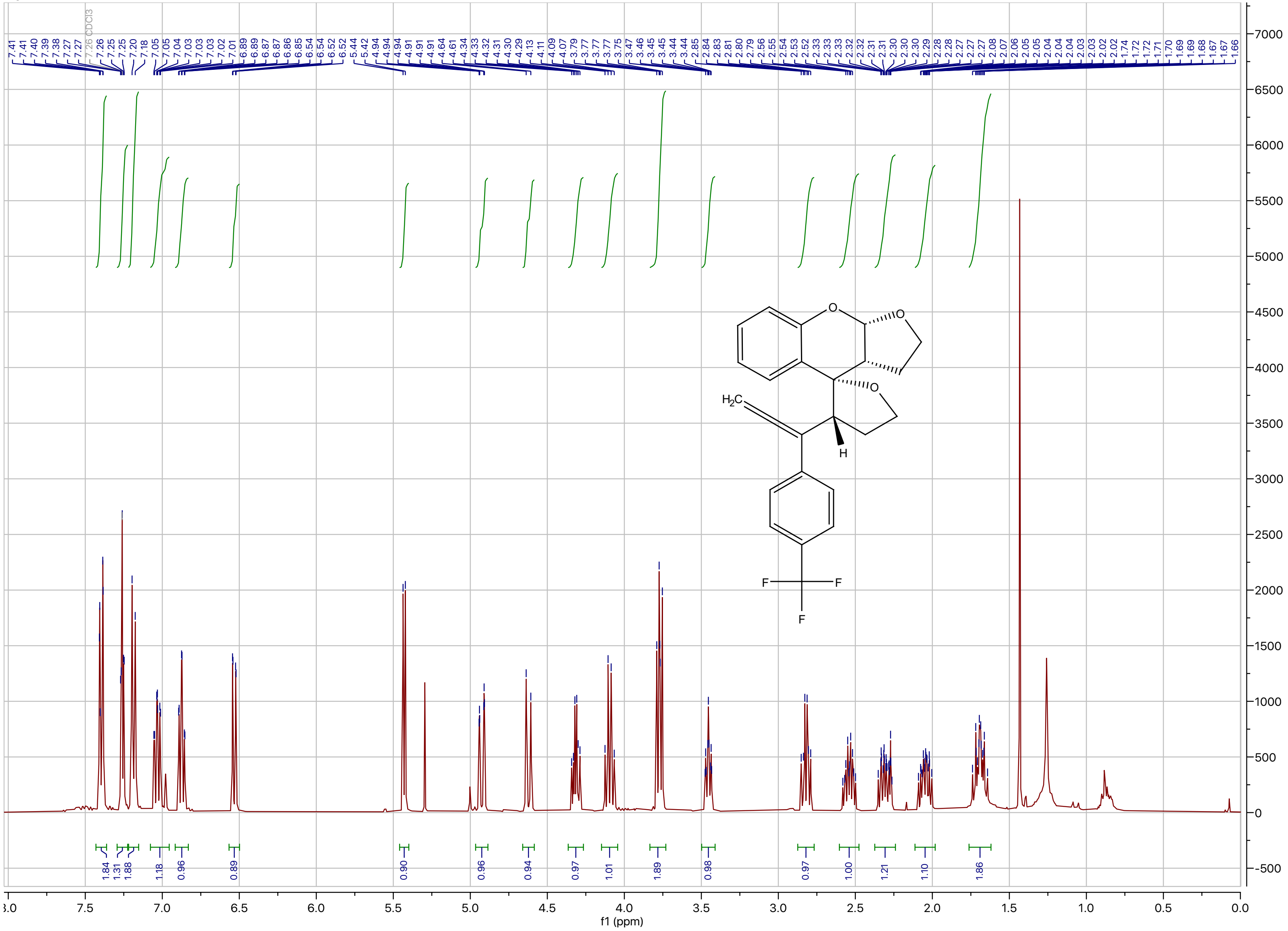

4j (101 MHz, $CDCl_3$)

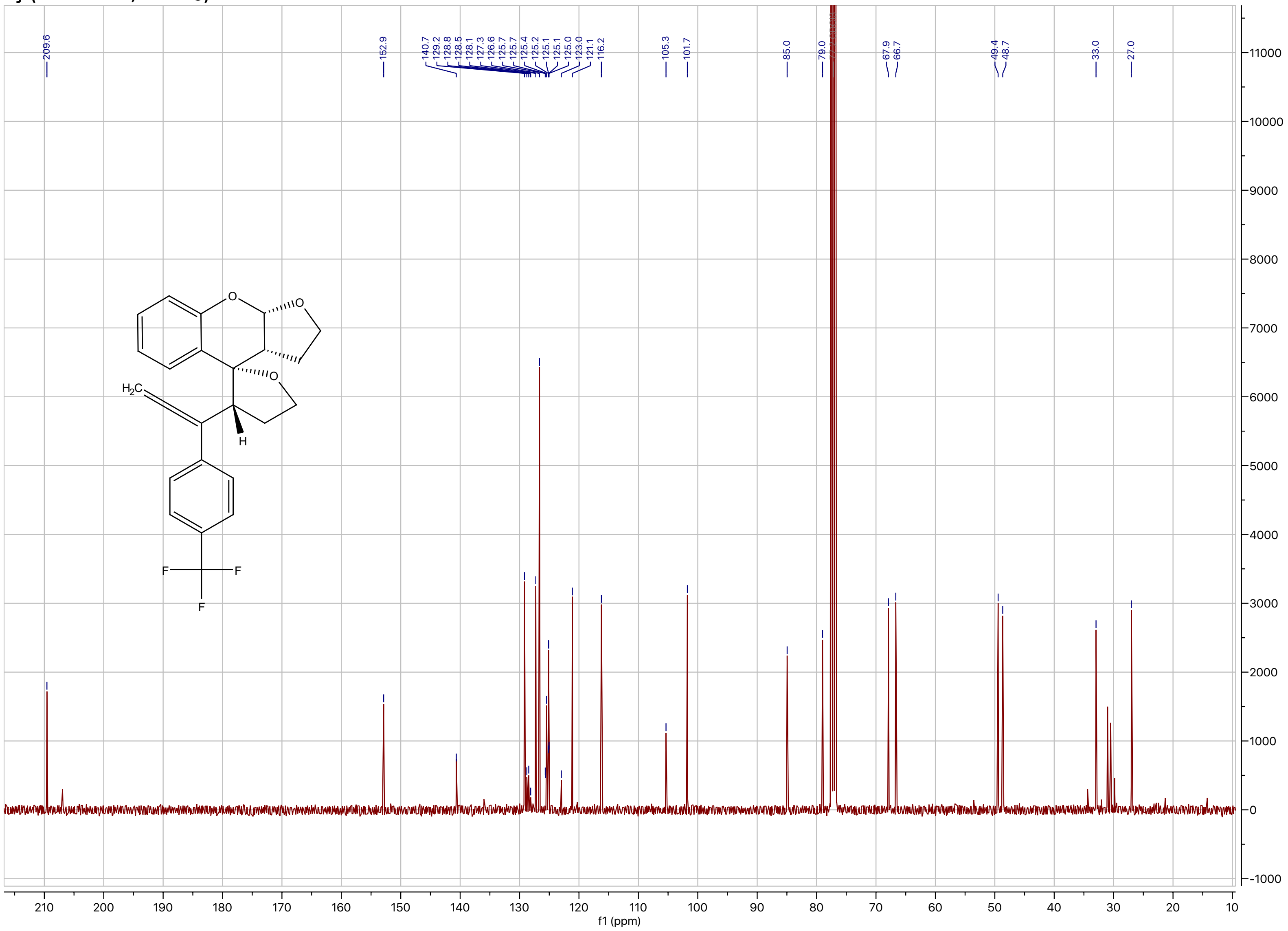

4k (400 MHz, CDCl$_3$)

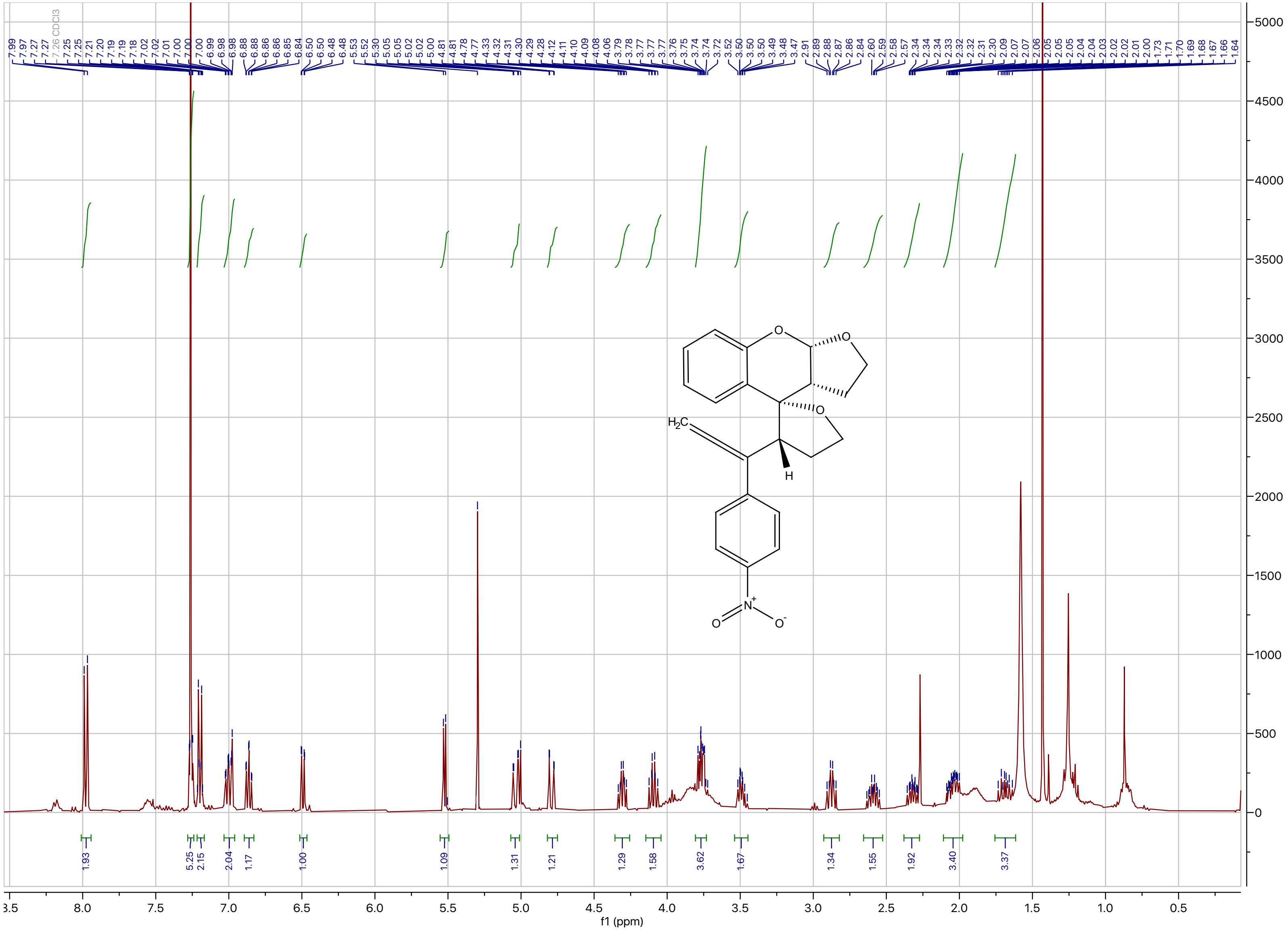

4k (101 MHz, $CDCl_3$)

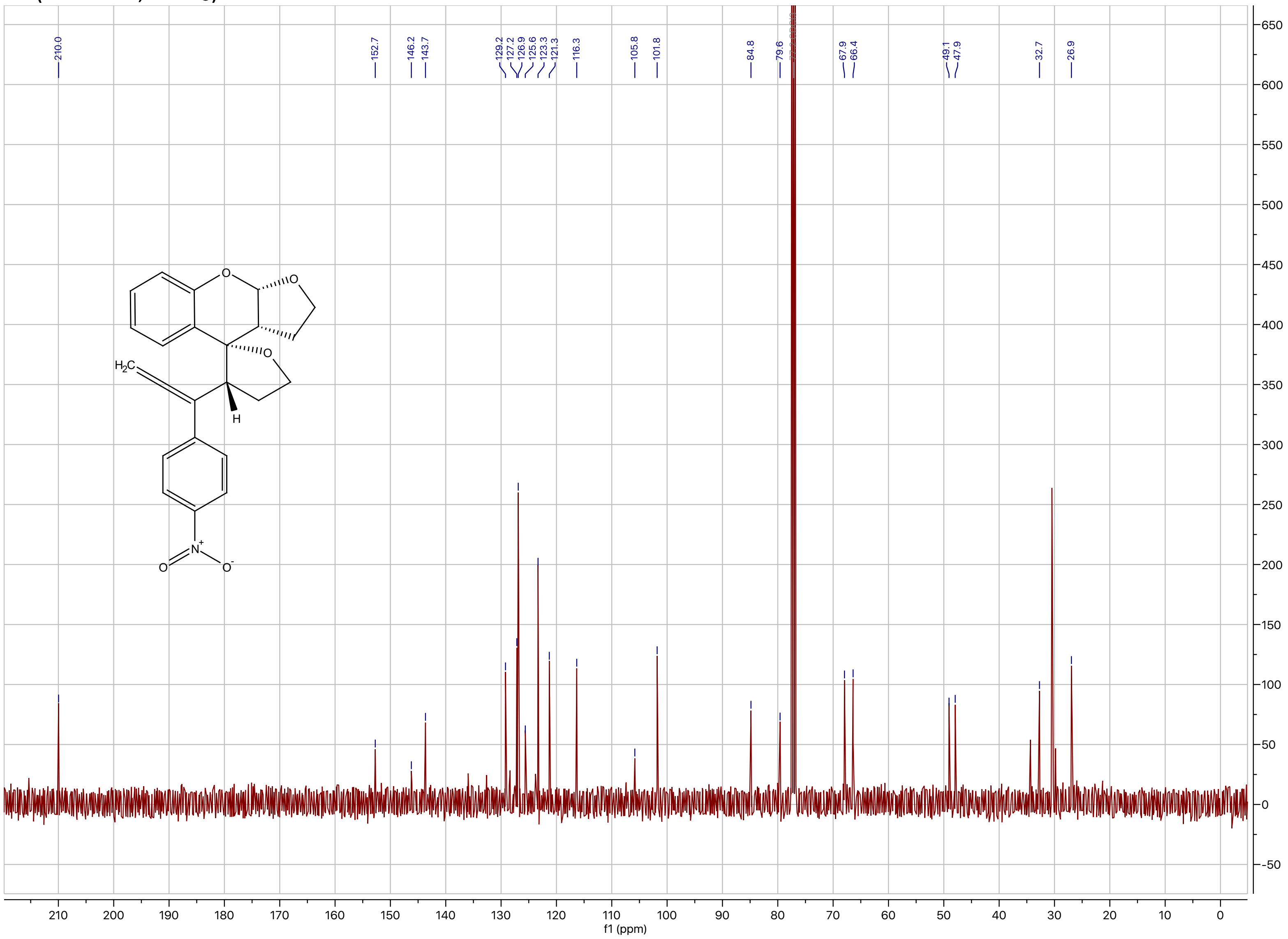

4l (400 MHz, $CDCl_3$)

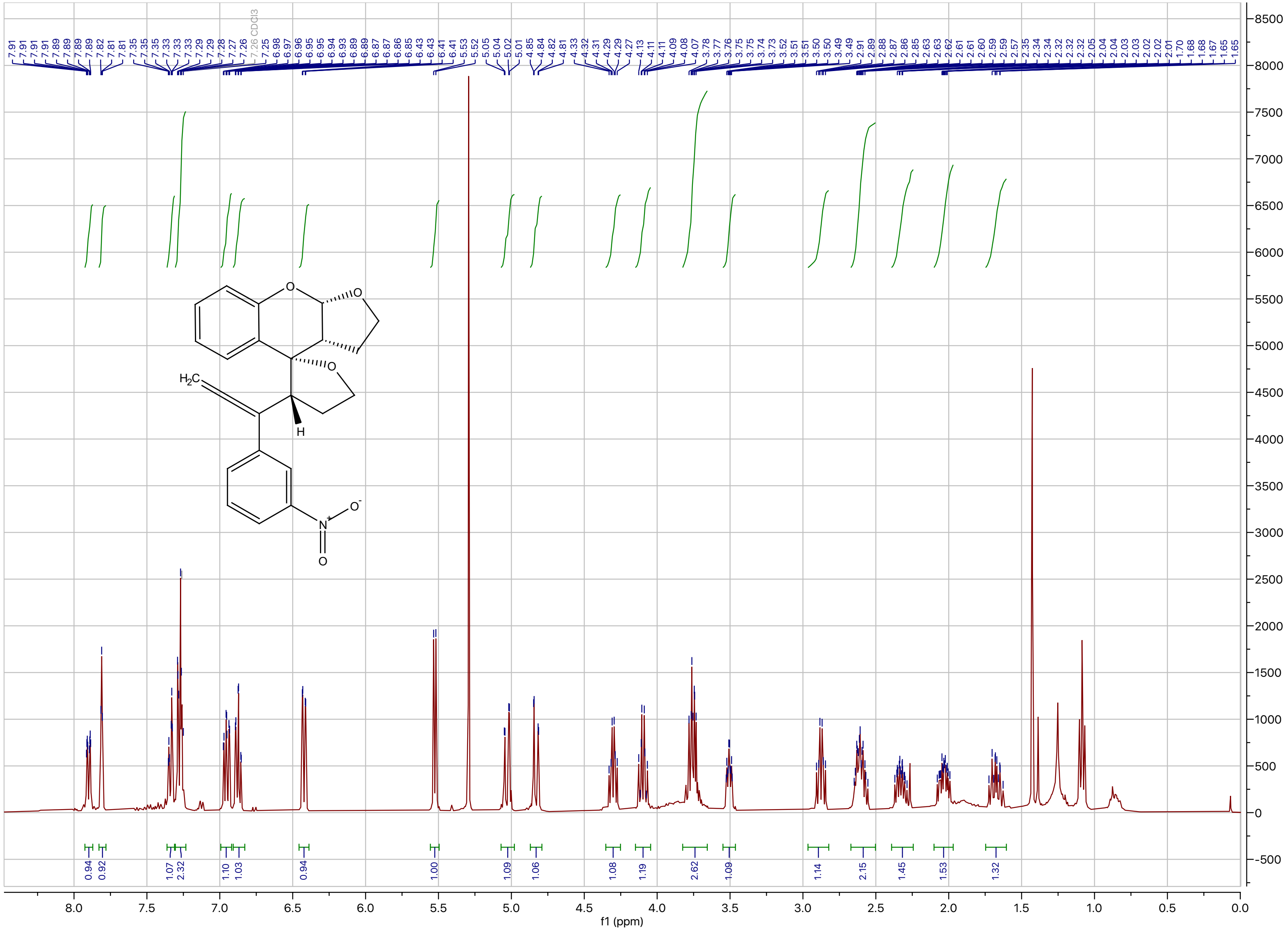

4l (101 MHz, $CDCl_3$)

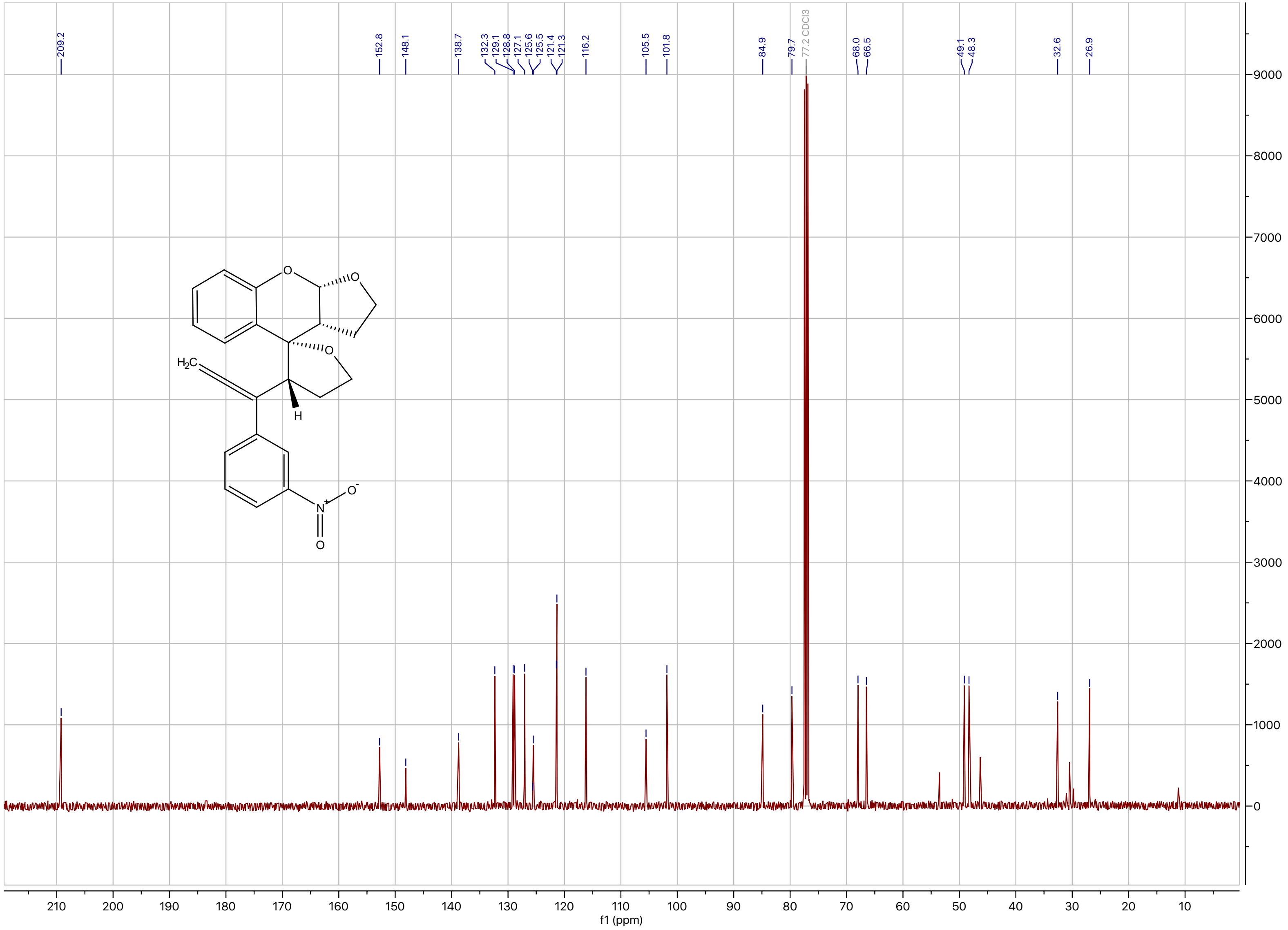

4m (400 MHz, $CDCl_3$)

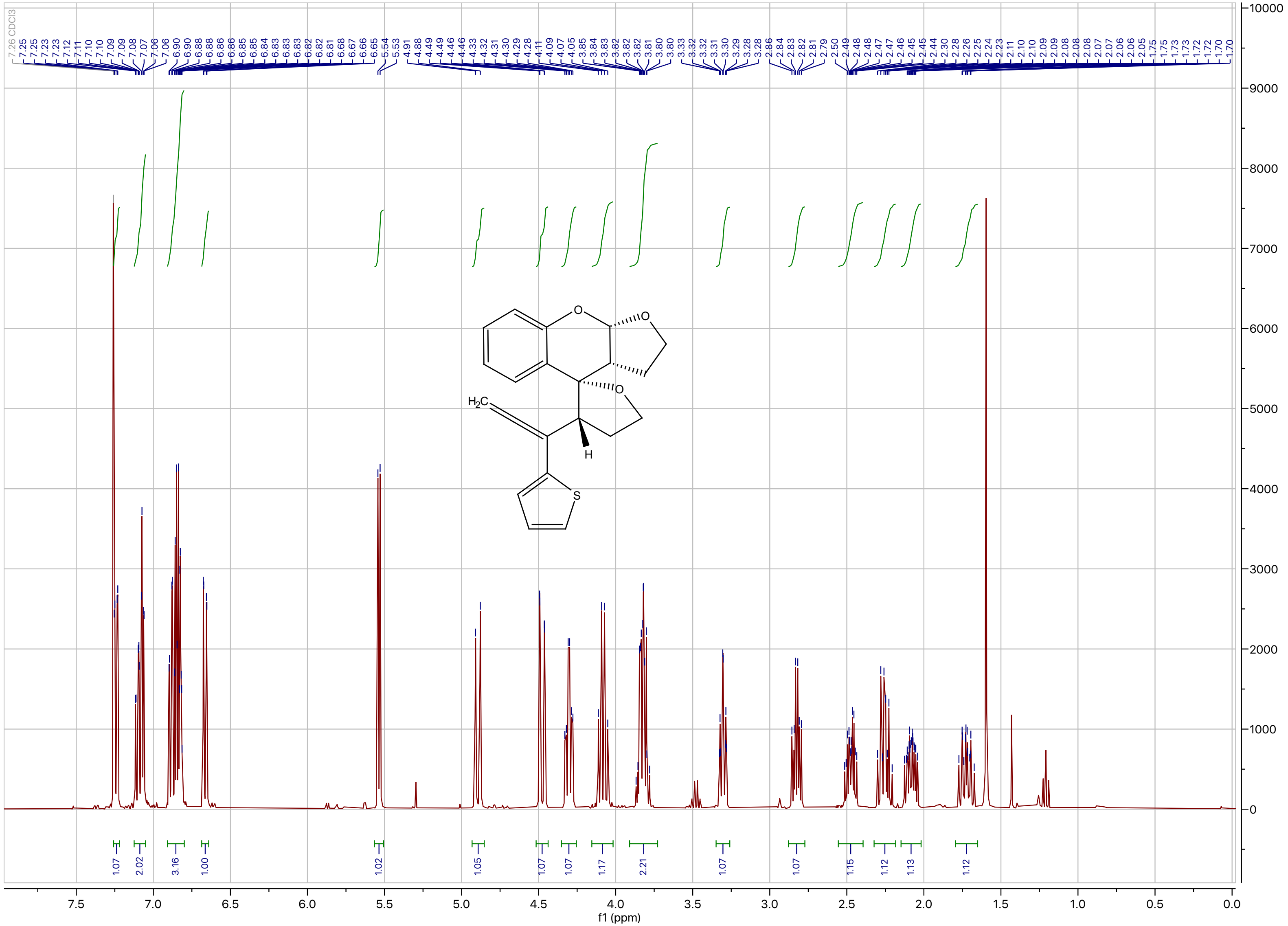

4m (101 MHz, $CDCl_3$)

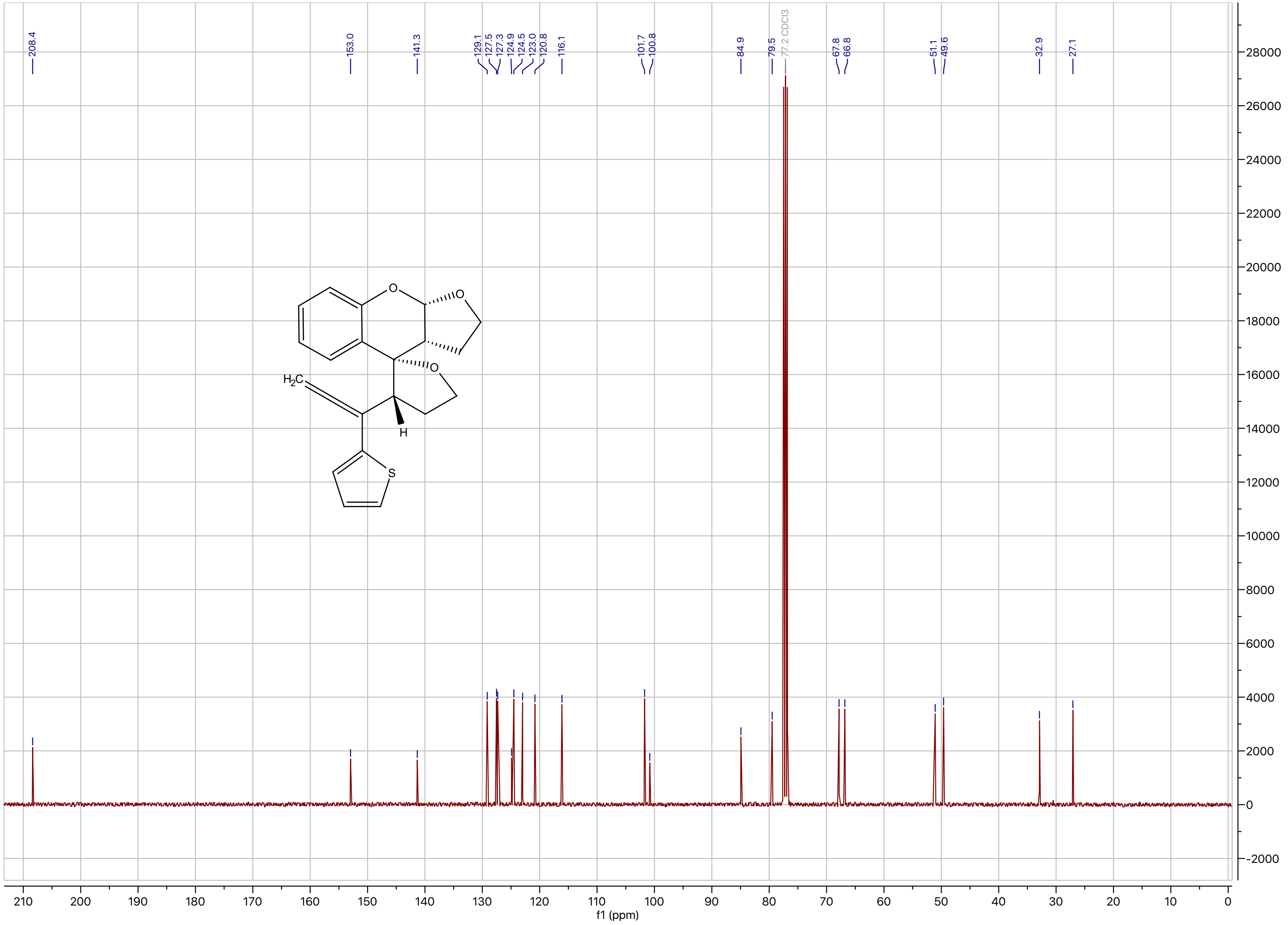

4n (400 MHz, $CDCl_3$)

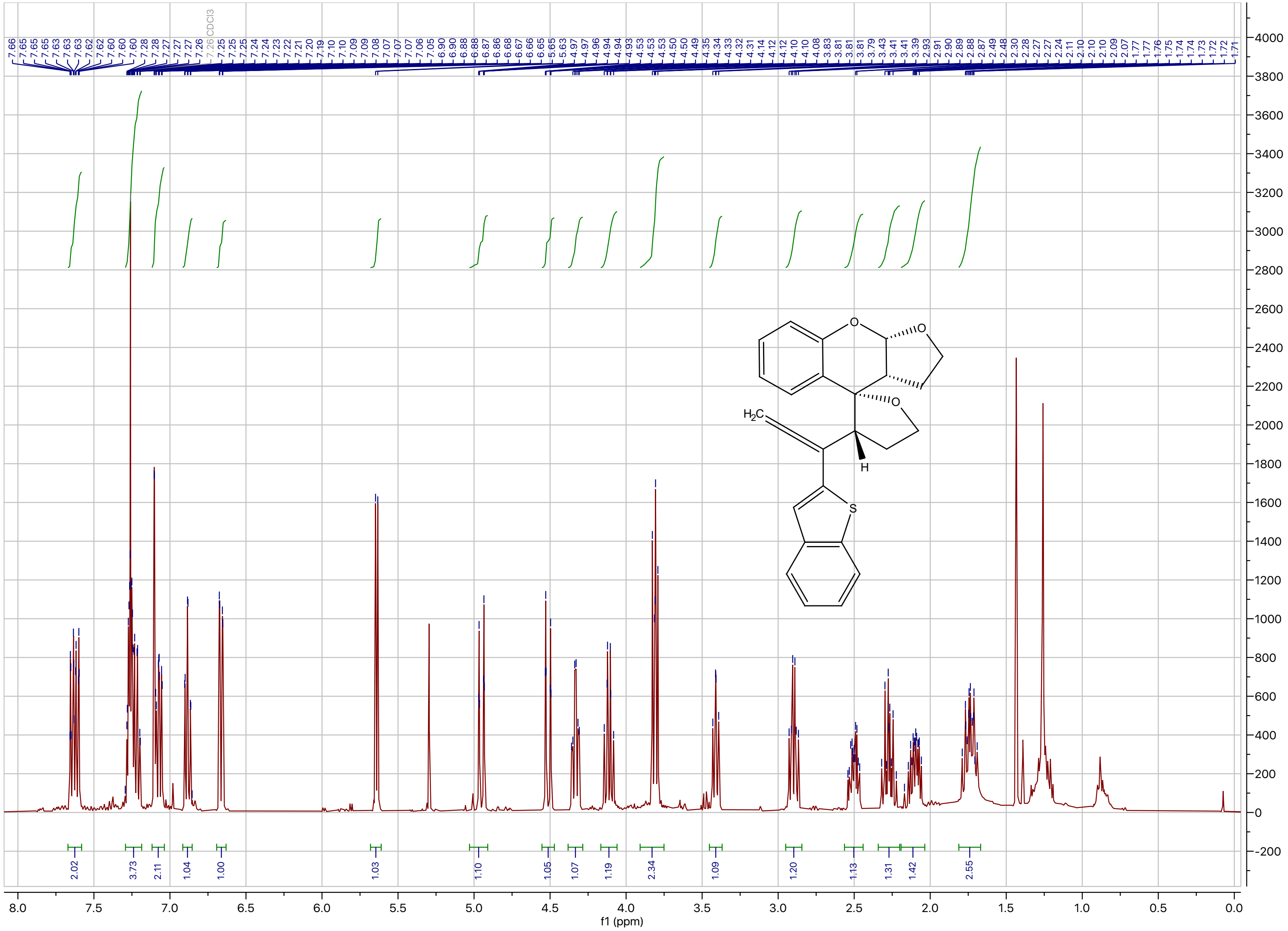

4n (101 MHz, $CDCl_3$)

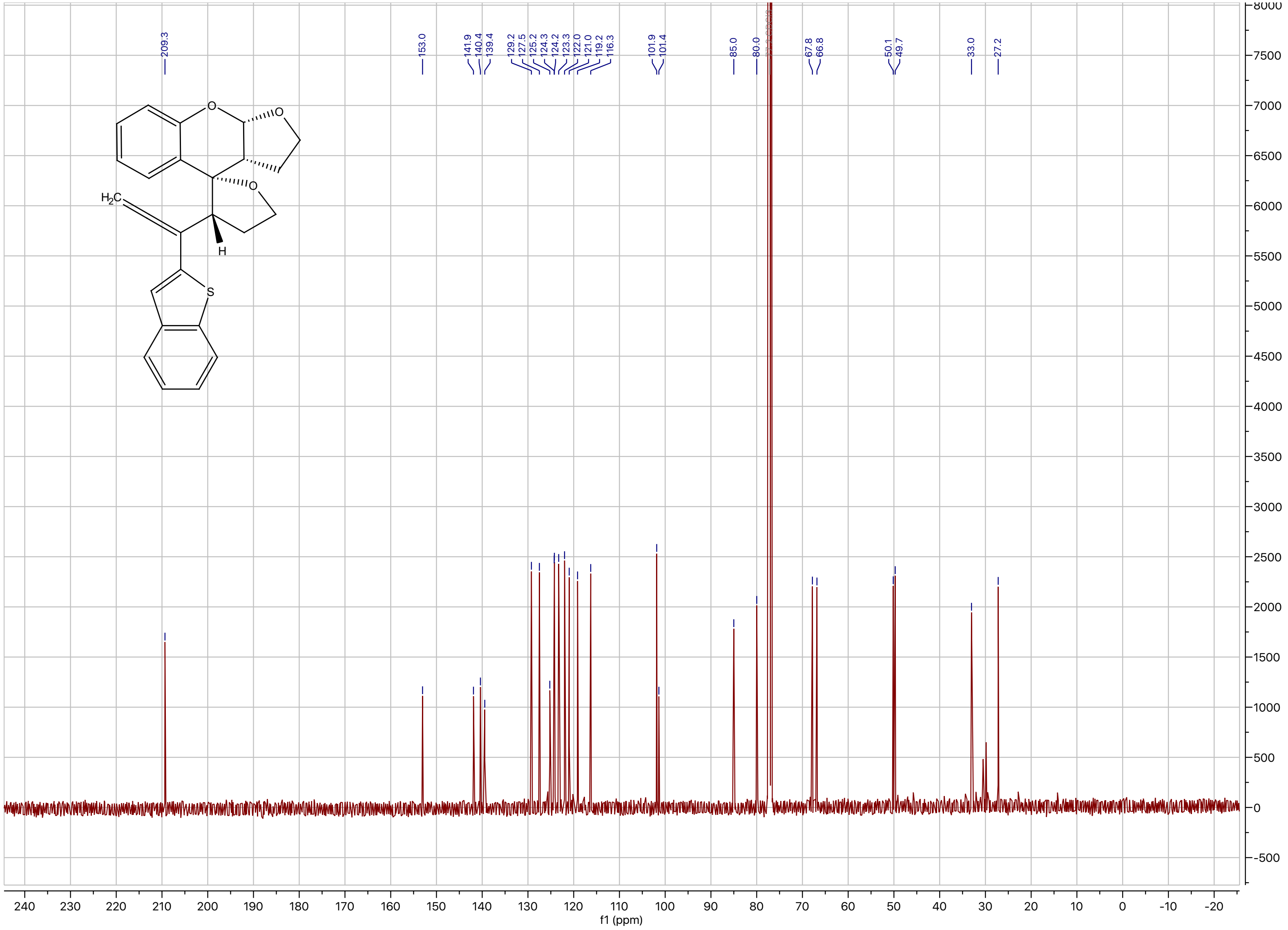

Supplement: Supplementary file 1 [file molecules-25-04976-s001.pdf]
